# Modification of a Common β‐diketiminate NacNac Framework via Sequential Lithiation and Small Molecule Insertion

**DOI:** 10.1002/chem.202303373

**Published:** 2024-01-26

**Authors:** Jennifer R. Lynch, Alan R. Kennedy, Jim Barker, Robert E. Mulvey

**Affiliations:** ^1^ Department of Pure and Applied Chemistry University of Strathclyde Glasgow G1 1XL UK; ^2^ Innospec Ltd. Oil Sites Road Ellesmere Port Cheshire CH65 4EY UK

**Keywords:** β-Diketiminates, Coordination Modes, Inorganic Chemistry, Ligand Synthesis, Lithium

## Abstract

A widely utilised class of ligands in synthesis and catalysis, β‐diketiminate (BDI) or NacNac compounds were initially considered innocent in the sense that they remained intact in all their applications. That changed when the γ‐C−H unit of their NCCCN backbone was found to engage in reactions with electrophiles. Here, we show that this special reactivity can be used advantageously to prepare tripodal modifications of the common NacNac ligand derived from 2,6‐diisopropylphenyl‐β‐methyldiketimine [NacNacH (Me, Dipp)]. Lithiation to give NacNacLi, followed by reactions with isocyanates, isothiocyanates and a carbodiimide, have afforded a series of tripodal NacNac variants having N,N,N,O; N,N,N,S; or N,N,N,N potential dentation sites, many of which have been crystallographically characterised. Distinct ligating modes of these new ligands have been elucidated through the crystal structures of their lithiated derivatives.

## Graphical abstract

Though non‐innocence of the popular NacNac (BDI) ligand derived from 2,6 diisopropylphenyl‐β‐methyldiketimine has been observed in a few studies, rarely has this non‐innocence been deliberately exploited to generate novel derivatives. Here, we show that tripodal modifications are accessible by a three‐step lithiation‐small molecule insertion‐hydrolysis protocol involving insertion of isocyanates, isothiocyanates, or a carbodiimide.

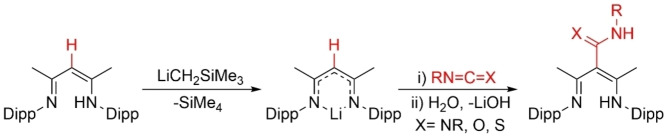

## Introduction

The β‐diketiminate (BDI) or NacNac ligand family has risen to prominence in many areas of research over the past decades, particularly in areas such as homogeneous catalysis, main group, FLP (frustrated Lewis pair) and low valent chemistry.[^1^, ^2^, ^3^, ^4^, ^5^] Their relatively simple and convenient syntheses, combined with their steric and electronic tunability by variation of their substituents make them an attractive ligand system for many researchers. The high tunability of the NacNac framework has naturally led to a diversity of structural variations, each intended to influence the steric and/or electronic properties of the ligand and its corresponding metal complexes. Increasing the steric characteristics associated with substituents at the N‐positions in NacNac has been a successful avenue of research in recent years, producing a wide range of novel, low oxidation state main group complexes, including formally those with Mg^I^,[^6^, ^7^, ^8^] Mg^0^,^[9]^ Al^I^,[^10^, ^11^, ^12^, ^13^] Ga^I[12,14]^ or Ge^I[15]^ centres. The relative stability of these species can largely be credited to the stabilisation provided by these adaptable bulky ligands.

Despite the variety of roles played by NacNac molecules across several areas of research, they are still mostly utilised as bidentate, monoanionic ligands. Several investigations have taken place into the inclusion of additional heteroatoms into the ligand framework of NacNac,[^16^, ^17^] primarily with the goal of increasing both denticity and the degree of steric protection offered to a metal centre. Introducing different heteroatoms into a single NacNac framework can also present any coordinating metal with a choice of coordination sites varying from ‘hard’ to ‘soft’, in accord with Hard‐Soft‐Acid‐Base (HSAB) theory.[^18^, ^19^, ^20^] Such ligands, containing a range of heteroatoms, can increase the potential for coordination to metals in heterobimetallic complexes, which can exhibit enhanced reactivity as has been documented in the literature.[^21^, ^22^, ^23^, ^24^, ^25^, ^26^, ^27^]

**Figure 1:**
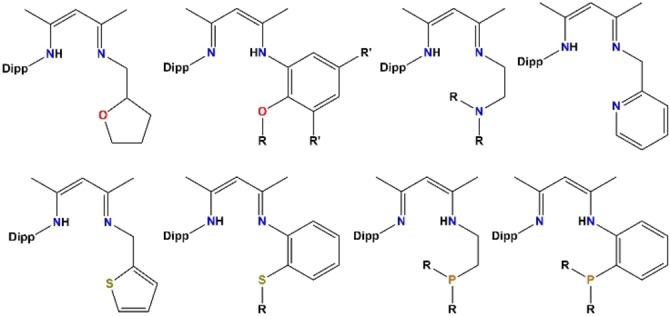
Representative literature examples of NNO, NNN, NNS and NNP NacNac‐based proligands.[^28^, ^29^, ^30^, ^31^, ^32^, ^33^]

**Figure 2:**
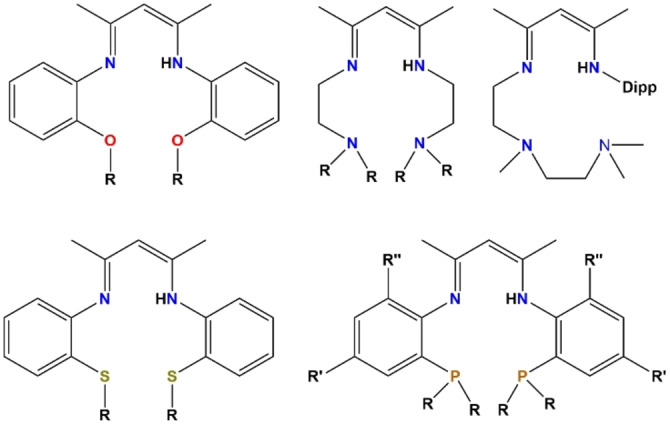
Representative literature examples of O,N,N,O; N,N,N,N; S,N,N,S; and P,N,N,P NacNac‐based proligands.[^34^, ^35^, ^36^, ^37^, ^38^, ^39^, ^40^]

Of particular relevance to our present research is the introduction of an additional heteroatom to the rudimentary NacNac framework, which could augment and/or vary the donor coordination properties of the ligand in having, for example O,^[28]^ N,[^29^, ^30^, ^31^] S,[^28^, ^32^] or P^[33]^ Lewis basic donor atoms in addition to the two N atoms of the parent NacNac molecule (Figure 1). Examples also exist where two additional donor atoms have been introduced, one on each pendant arm forming, for example, O,N,N,O[^34^, ^35^], N,N,N,N[^36^, ^37^], S,N,N,S^[38]^, or P,N,N,P[^39^, ^40^] coordination sets (Figure 2).

A common thread links the tri‐ and tetra‐dentate NacNac frameworks discussed, namely the additional heteroatoms have been installed within the substituents attached to the original NacNac N atoms. However, an alternative approach available for the addition of an arm to the original NacNac frameworks, is one which at the same time could also disable a potential site of unwanted reactivity. To explain, the HOMO‐1 of NacNac, where here NacNac refers specifically to the N‐deprotonated aminoimine, 2,6‐diisopropylphenyl‐β‐methyldiketimine [NacNacH (Me, Dipp)] labelled for brevity throughout the text as NacNac, has been computed to sit at the γ‐carbon position of the ligand backbone, instilling this position with significant nucleophilic character.^[2]^ The reactivity at this site with a range of small molecules has been well documented, with O_2_,^[41]^ CO_2_,[^42^, ^43^], cyanides,[^44^, ^45^] isocyanates,[^43^, ^46^, ^47^] isothiocyanates,^[48]^ alkenes and alkynes,[^49^, ^50^, ^51^] aldehydes,[^52^, ^53^] ketones,[^54^, ^55^, ^56^] ketenes,[^57^, ^58^] pyridines,^[59]^ and more.[^60^, ^61^] However, rarely do these studies specifically focus on the use of these new small‐molecule‐inserted NacNac species as potentially useful proligands in their own right, instead concentrating mainly on the intermediate metal complexes formed during their synthesis. Deliberate insertion of different heteroatom‐containing units into the γ‐carbon position would provide additional sites of denticity and perhaps stability on some metal centres that might turn unwanted non‐innocent reactivity of a classical NacNac ligand into a useful new class of tripodal NacNac ligand, similar to that seen for β‐triketiminates, which have three identical arms.[^62^, ^63^, ^64^] With this reasoning in mind here we exploit similar reactivity to generate tripodal ligands made up of different ligating arms.

## Results and Discussion

**Scheme 1:**
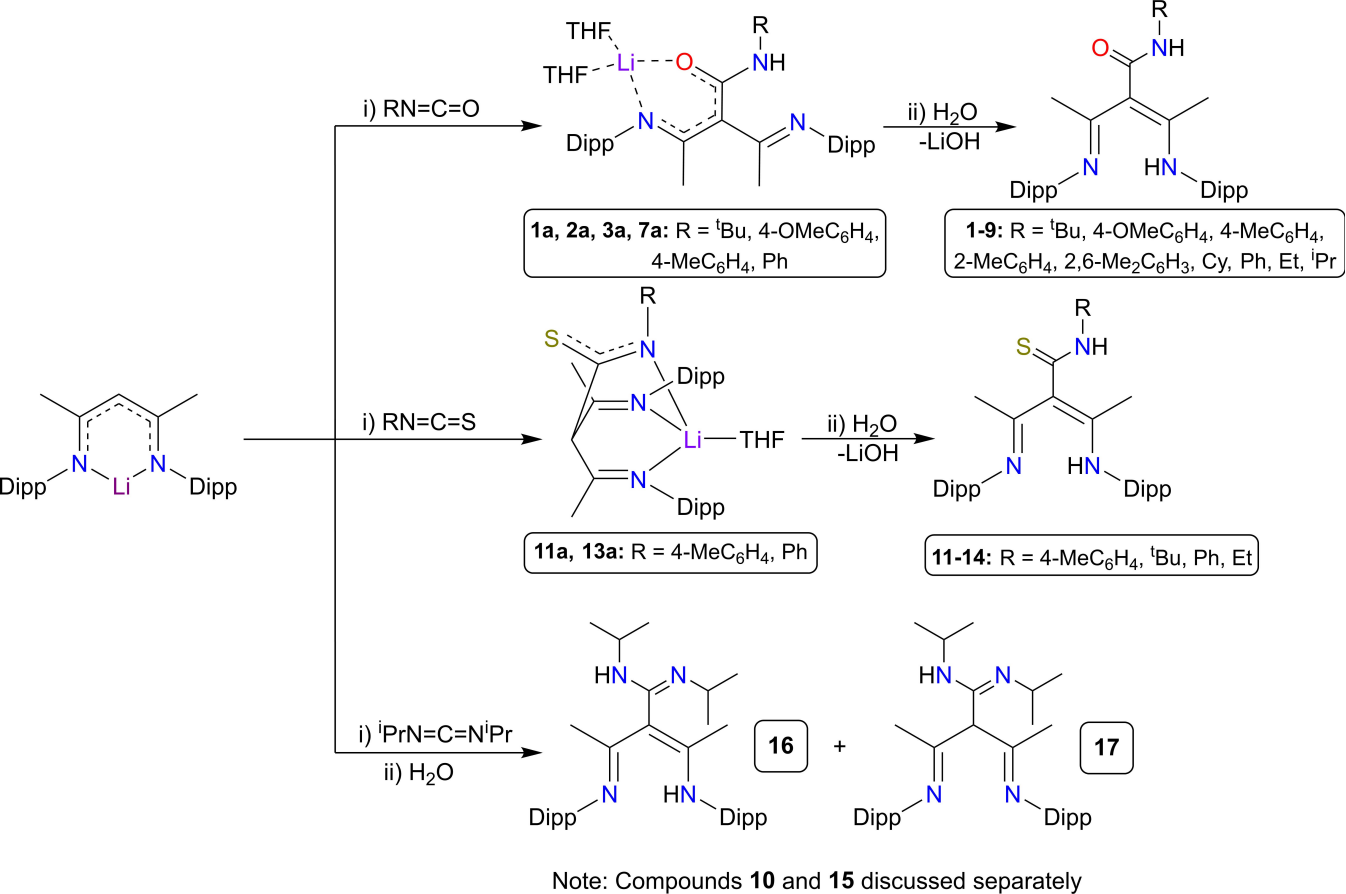
Synthesis of the series of proligands **1**–**17**, and their respective lithium intermediates, established in this work.

**Figure 3:**
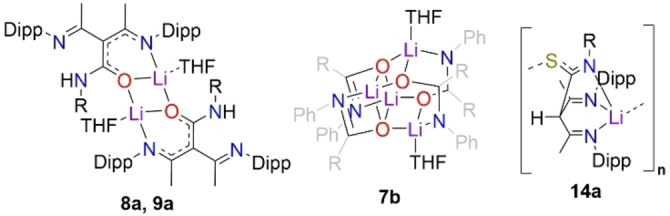
Other structural motifs of lithium complexes highlighting bonding modes of the NacNac‐based proligands made in this work.

Scheme 1 presents a new family of tripodal NacNac proligands, whereby the insertion of a heteroatom‐containing unit into the γ‐carbon position provides the ligand with additional sites of coordination to metal centres and utilises a site, the γ‐carbon position, that is generally seen as the source of problematic and unwanted reactivity. In addition to the synthesis and characterisation of these new proligands, this study showcases some representative examples of the lithium precursors to these species, which give exemplars of the ligating characteristics possessed by these ligands. The most common structural arrangements for these lithium intermediates is shown in Scheme 1, with the more unusual structural motifs shown in Figure 3, where lithium complexes are identified by the numeral of the proligand, followed by a letter.

These lithium precursors were found to be isolable as intermediates from the synthetic reactions of the ligands (Scheme 1), usually upon addition of the Lewis donor solvent THF to a hexane suspension to aid solubility and crystallisation. Alternatively, once the proligands have been synthesised, they can subsequently be reacted with a suitable organolithium base [^n^BuLi and LiCH_2_Si(CH_3_)_3_ were used here], in the presence of a donor solvent. Each proligand system will now be discussed in turn.

### N,N,N,O ligands

**Figure 4:**
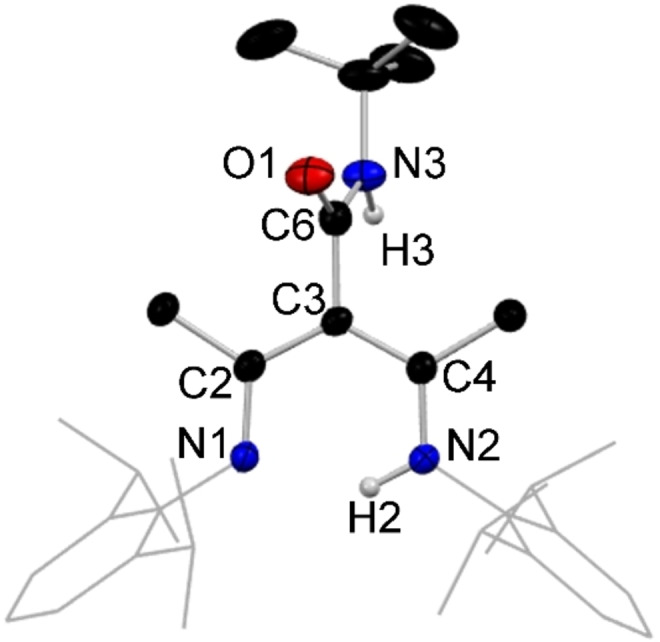
Molecular structure of **1**, representative of structures **1**–**9**, with H atoms omitted except for N−H, and Dipp groups shown as wire frame for clarity. Thermal ellipsoids are displayed at 40 % probability level.

Tripodal NacNac derivatives possessing potential N,N,N,O coordination sites, formed via a nucleophilic reaction of NacNacLi and an isocyanate, make up most of the new species prepared in this work. Scheme 1 gives the general reaction for this type of species. Following the preparation of NacNacH, via a published condensation reaction of acetylacetone with two stoichiometric equivalents of 2,6‐diisopropylaniline and its subsequent lithiation to NacNacLi,^[65]^ a range of isocyanates were inserted into the γ‐CH bond of the NacNacLi species (for more details, see the supporting information). Structurally characterised lithium intermediates from these isocyanate reactions and the proligands formed via subsequent hydrolysis follow the reaction in Scheme 1. Analogous structural cores were observed for the proligands in several variations of the organic group R [i. e., ^t^Bu (**1**), 4‐OMeC_6_H_4_ (**2**), 4‐MeC_6_H_4_ (**3**), 2‐MeC_6_H_4_ (**4**), 2,6‐Me_2_C_6_H_3_ (**5**), Cy (**6**), Ph (**7**), ^i^Pr (**8**), Et (**9**)]. Figure 4 shows an X‐ray crystallographically determined structure of one of the proligand products [(MeCNH‐Dipp)(MeCN‐Dipp)C(^t^BuNHCO)], **1**, obtained using tert‐butyl isocyanate, ^t^BuNCO.

**Figure 5:**
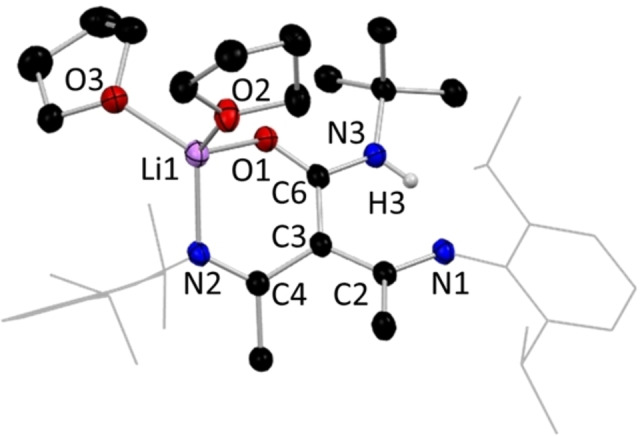
Molecular structure of **1 a**, representative of structures **2 a**, **3 a**, and **7 a**, with H atoms omitted except the N−H, and Dipp groups shown as wire frame for clarity. Thermal ellipsoids are displayed at 40 % probability level.

The introduction of the third arm at C3 in **1** has increased the number of heteroatoms from two to four. From its metric data it is discerned that the bonding in the new tripodal arm is highly localised [C6=O1, 1.225(2) Å, C6−N3, 1.352(2) Å]. The structure contains two distinct N−H bonds available for deprotonation, with the diketiminate N(2)−H(2) bond being cleaved preferentially in the corresponding lithium complex (**1 a**) (view the SI for details). This is evidenced by the observation that the amide N−H bond of the new proligands remains intact in all the characterised examples of the lithium complexes of these species, as confirmed by both X‐ray crystallographic and spectroscopic data, while the di‐deprotonated, di‐lithiated species remain elusive despite several attempts to synthesise them. Figure 5 shows a representative structure of the ligated lithium species characterised from these reactions in the monomeric bis‐THF solvate [{(MeCN‐Dipp)_2_C(^t^BuNHCO)}Li ⋅ 2THF], **1 a**.

**Figure 6:**
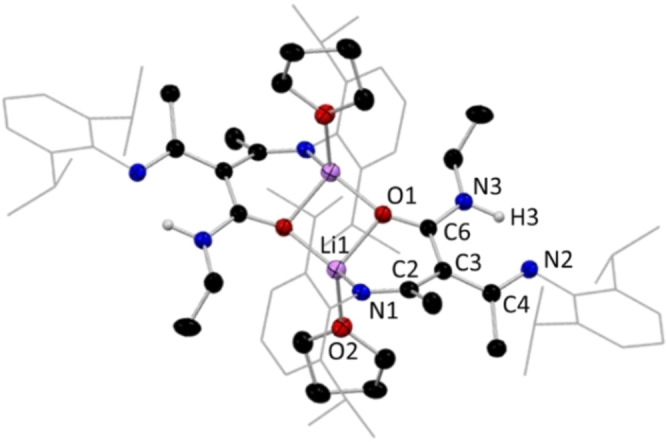
Molecular structure of **9 a**, with H atoms omitted except for N−H, and Dipp groups shown as wire frame for clarity. Thermal ellipsoids are displayed at 40 % probability level.

**Figure 7:**
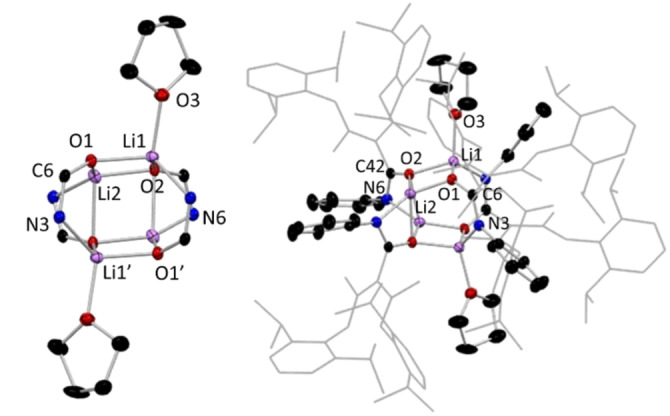
Molecular structure of **7 b**, with NacNac and aryl groups omitted for clarity, view through cubic core (LHS) and structure with NacNac and aryl groups shown as wireframe (RHS). Thermal ellipsoids are displayed at 40 % probability level. Co‐crystallised solvent molecules omitted.

While it was considered possible that the lithium derivative of isocyanate inserted ligands might adopt an NNO style bonding mode in the absence of ethereal solvent, no success was found in isolating a crystalline sample of an N,N,N,O type tripodal NacNac intermediate without the aid of coordinating solvents to break up the likely polymeric species. As a result, in **1 a** and the other monomers of this type, the lithium centre is solvated by two THF molecules, in addition to the isocyanate‐derived O atom and one NacNac N atom, leaving the other N atoms of these two units uncoordinated to Li. These atoms surround Li in a distorted tetrahedral geometry, with τ_4_=0.82, where τ_4_=1 represents a perfect tetrahedral geometry and τ_4_=0 represents a perfect square planar geometry.^[66]^ The central carbon atom of the NacNac backbone shows trigonal planar coordination, denoting the lack of a hydrogen atom at this position. Inspecting the comparative data in Table 1, it is clear that those structures with an inserted isocyanate‐derived arm have notable differences in the bond length pattern in the NacNac N−C−C−C−N linkages. For example, in **1** the two N−C and two C−C bond lengths show differences of only 0.005 Å and 0.015 Å, respectively, while replacing H^+^ by Li^+^ in **1 a** produces little effect with corresponding values of 0.0032 Å and 0.0134 Å, respectively. This deviates from Power's comparison of NacNacH and NacNacLi(THF or Et_2_O), where localisation is far advanced in the former, but the π‐bonding becomes significantly more delocalised in the latter. Therefore, since **1 a** and Power's lithium structures are all monomers, it appears that it is the introduction of the isocyanate‐derived third arm into the NacNac backbone that is a factor in keeping the delocalisation found in the N−C−C−C−N linkage in **1**. In contrast, localisation is pronounced in the isothiocyanate‐derived lithium compound **14 a**, with much shorter N−C bonds and much longer C−C bonds [1.280(4) Å and 1.524(3) Å, respectively] within the symmetrically equivalent moieties of its N−C−C−C−N bridge (see below). Here, replacing Li^+^ by H^+^ does cause a substantial change as in **14**, namely delocalisation is more evident with N−C bonds lengthening to a mean value of 1.3275 Å and C−C bonds shortening to a mean value of 1.4257 Å. It was established that the ratio of donor solvent incorporated into the crystal structures of the lithium intermediates of this N,N,N,O class was variable, with dimeric and tetrameric variants also being observed, with only single or hemi THF solvation respectively, instead of the double THF solvation preferred in the monomeric case. Hydrolysis to the parent ligand proceeds analogously in these higher aggregates akin to that in the monomeric proligand types such as **1**. Intramolecular hydrogen bonding of the N−H ⋅⋅⋅ N type, between the arms of the original NacNac backbone, is ubiquitous in all structures that retain a NH group. In the hydrolysed ligand structures **1**, **7.1**(note this is a THF solvate of **7**, see S.I.), **10**, **12**, **15**, **16**, and **17** there does not appear to be any significant classical intermolecular hydrogen bonding between units of the target molecules, despite the presence of good donor and acceptor groups such as amide functionalities. Instead, these unused donor and acceptor units make a variety of long range and thus presumably weak interactions as exemplified by the N−H ⋅⋅⋅ π interaction between the amide group and a benzene ring in **1**. In structures **3** and **4** (whose R groups are 4‐OMeC_6_H_4_ and 4‐MeC_6_H_4_ respectively) there is dimerisation via H bonding between neighbouring amide units, giving a centrosymmetric ring with R2/2(8) graph set notation.^[67]^ The thioamide **11** displays similar hydrogen bonded dimers, albeit with S substituted for O. In contrast to these dimeric motifs, where the R group is ^i^Pr (**8**) or Et (**9** and **14**, for NNO and NNS ligands respectively) the amide or thioamide groups link into polymeric chain motifs with C1/1(4) notation. In **5**, **6** and **7** (2,6‐Me_2_C_6_H_3_, Cy and Ph groups) ligand units also hydrogen bond into chains but this is via interactions with alcohol solvent molecules and no intermolecular hydrogen bonds are seen directly between ligand units Lithium centres in dimeric **9 a**, [{(MeCN‐Dipp)_2_C(EtNHCO)}Li⋅THF]_2_ (Figure 6), are coordinated to three oxygens and one nitrogen atom in a distorted tetrahedral geometry, τ_4_=0.75, similar to that displayed in monomeric **1 a**.^[66]^ In **9 a** both O atoms in the central 4‐membered (LiO)_2_ ring belong to inserted isocyanate units with the third O atom coming from terminal THF solvation. Again, the central C atom of the NacNac backbone shows trigonal planar coordination, emphasising its sp^2^ nature. Viewing the metrical data in Table 1, we see a similar pattern for **9/9 a** to that for **1/1 a**, with only minute differences in the delocalisation on replacing H^+^ by Li^+^. Isolation of a dimeric lithium intermediate, as **9 a**, was unsurprising, as previous work by our group had reported the crystallographic characterisation of an analogue of this structure, **8 a**, wherein isopropylisocyanate, ^i^PrNCO, was used instead of ethylisocyanate, EtNCO.^[68]^ In addition to the isolation of the monomeric and dimeric lithium aggregates, a tetrameric lithium intermediate of our N,N,N,O tripodal NacNac family was isolated and characterised. This example, [{(MeCN‐Dipp)(MeCNH‐Dipp)C(PhNCO)}Li]_4_ ⋅ 2THF, **7 b** (Figure 7), was made using phenylisocyanate, PhNCO. In contrast to monomeric **7 a**, **7 b** is a tetramer with partial THF solvation. In the tetramer, each metal centre coordinates to three oxygen atoms, forming an open cube arrangement, whereby two symmetrically equivalent edges of the Li−O cube (O1‐Li1’ and O1’‐Li1) have been opened via C(R)N(R’) bridges. The highly oxophilic nature of the two distinct lithium centres encourages arrangements such as that seen in **7 b**, wherein each metal centre is able to maximise the number of oxygen atoms it connects to, a structural feature often documented in the literature.[^69^, ^70^, ^71^, ^72^] For **7 b**, we see that each metal centre is bonded to three oxygen atoms, with Li1 connecting to an oxygen each from two ligand units, with the remaining Li1−O bond to a THF unit, while the coordination sphere of Li2 contains three oxygen atoms from a different ligand unit. Both Li1 and Li2 also bind to an isocyanate‐derived N atom.

**Table 1:**
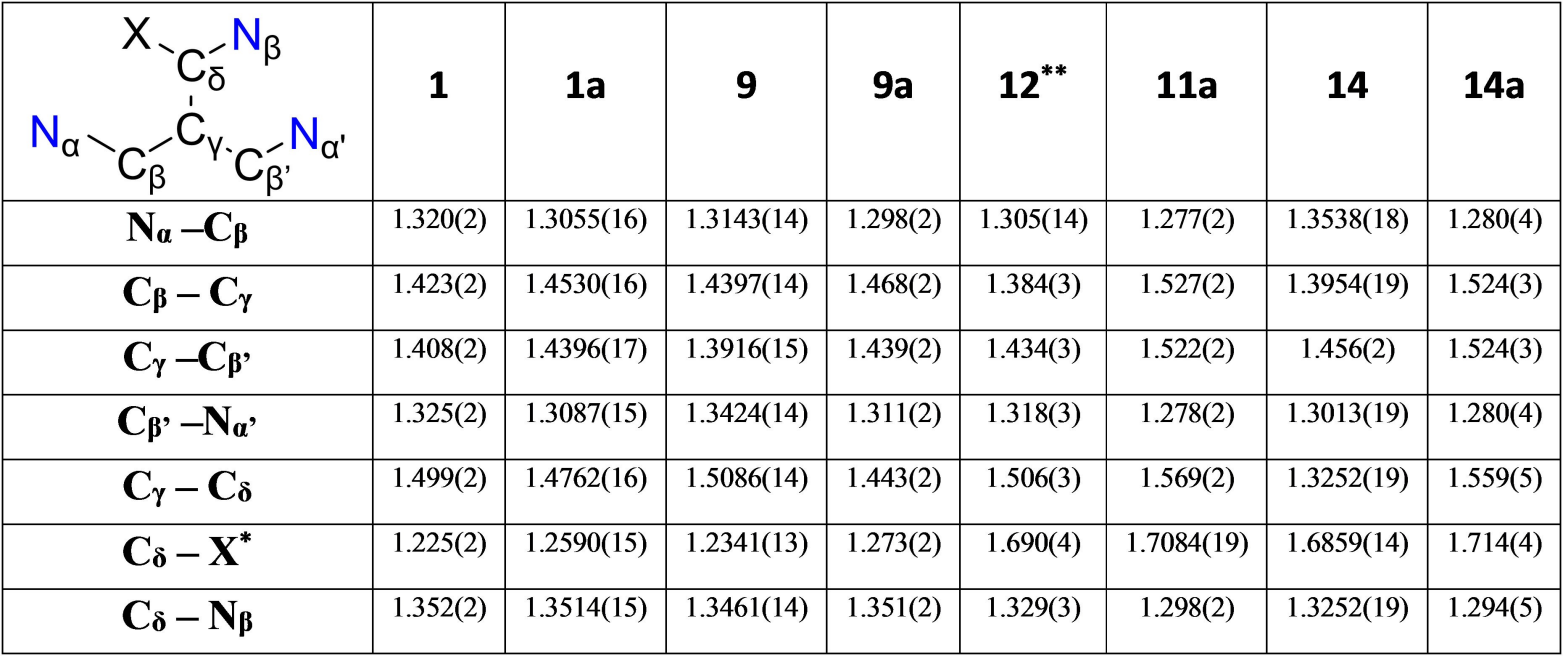
Selected comparative bond lengths (Å) in proligands and lithium derivatives.

*1/**1 a**/**9**/**9 a** X=O, **12**/**11 a**/**14**/**14 a** X=S ** as data for proligand **11** are poor, close relative **12** was used as a comparison for **11 a**

The cube‐like structure of **7 b** displays close to single C−O bond lengths [O1 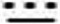
C6, 1.296(3) Å; O2C42, 1.304(3) Å] and two double C=N bond lengths equivalent within experimental error [C6N3, 1.306(4) Å; C42N6 1.299 (4) Å], in the PhNCO inserted arm in the two symmetrically distinct units making up the tetramer. THF‐free Li2 coordinates to two oxygen atoms from separate isocyanate units and is chelated by the oxygen and nitrogen of a third “isocyanate‐derived” ligand unit. Li1 has a mean bond angle of 109.1° over a range of 94.1(2)°–120.7(2)° (τ_4_=0.87) with corresponding values for Li2 of 105.5° in the range of 65.91(17)°–135.4(2)° (τ4=0.69). However, these crystallographically distinct Li atoms are indistinguishable in C_6_D_6_ solution as only one resonance is seen in the ^7^Li NMR spectrum of **7 b** under the conditions studied.

**Figure 8:**
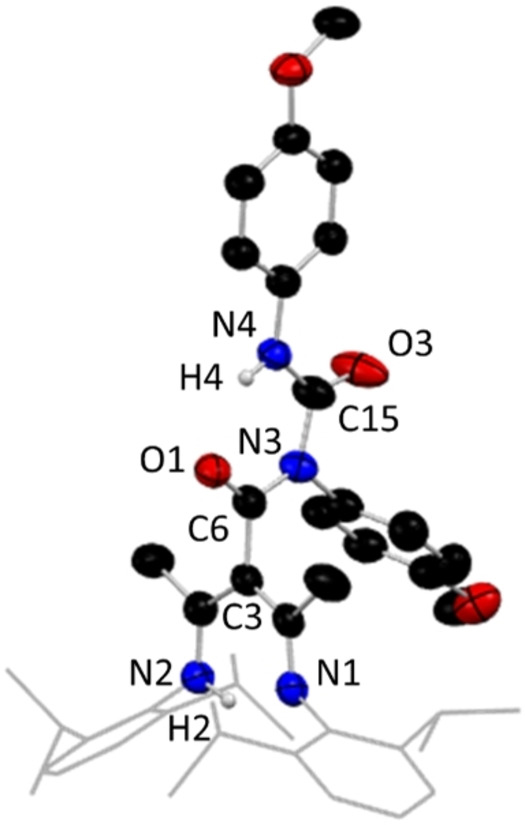
Molecular structure of **10**, with H atoms omitted except for N−H, and Dipp groups shown as wire frame for clarity. Thermal ellipsoids are displayed at 40 % probability level.

**Scheme 2:**
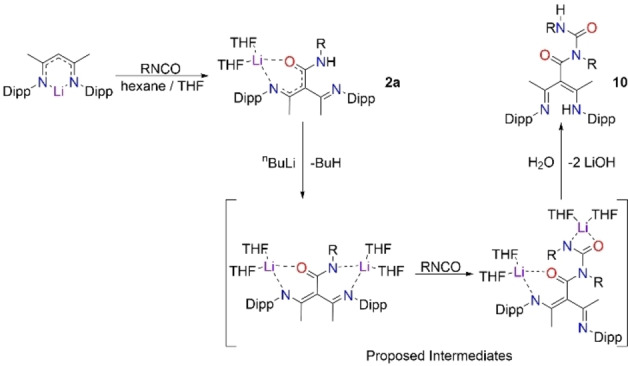
Proposed route for formation of metal‐free **10** showing double isocyanate insertion.

In these experiments, the source of lithium metal used in the synthesis of NacNacLi, was typically ^*n*^BuLi, as a solution in hexanes, which requires periodic titration to determine the concentration of active reagent within it.^[73]^ The importance of titrating this reagent before each use became clear after the discovery of a small number of crystals of lithium‐free [(MeCNH‐Dipp)(MeCN‐Dipp)C{C(O)N(C_6_H_4_OMe)C(O)N(H)C_6_H_4_OMe}], **10** (Figure 8), within a crystalline sample of [(MeCNH‐Dipp)(MeCN‐Dipp)C(4‐OMeC_6_H_4_NHCO)], **2**. The crystals of **10** were of a distinctively different scale and shape to that of the desired product ligand and so were easily identified as distinct under a microscope. Changing the lithium source used from a solution of ^*n*^BuLi to solid LiCH_2_Si(CH_3_)_3_ to give better stoichiometric control prevented any further instances of the over lithiation suspected to be behind the formation of **10** (see Scheme 2), which contains two inserted isocyanate units.

The crystallographic data for **10** show highly localised bonding in the central‐C(=O)N(R)C(=O)NH(R) unit [C15=O3, 1.218(3) Å; C15‐N4, 1.346(3) Å; C15‐N3, 1.437(3) Å; C6‐N3, 1.398(3) Å; C6=O1, 1.223(3) Å]. Similar to the structures of **1** and **1 a**, the γ‐carbon C3 of its NacNac backbone shows trigonal planar coordination. The presence of trace amounts of **10** could mean that a slight excess of both lithium reagent and isocyanate were present in the reaction on this occasion, resulting in a species containing two inserted isocyanate units. Attempts at a rational synthesis of **10** produced a mixture of crystalline products, with **2** being the major product, and trace amounts of **10** having to be isolated by hand from the product mixture. One possible route to product **10** could be by further deprotonation of the intermediate lithium species, **2 a**, by an excess of lithium reagent allowing a second insertion of isocyanate at the remaining ‐NH unit, which, when hydrolysed, produces **10** in small quantities alongside **2** (Scheme 2). While our attempts at isolating the di‐lithiated intermediate species in the proposed formation of **10**, have so far been unsuccessful, the reproducibility of **10**, even in small amounts, suggests that it may be possible to construct dianionic tripodal ligands.

The discussed compounds **1**, **1 a**, **9 a**, and **7 b** are typical examples of the ligands and associated lithium species obtainable when an isocyanate unit inserts into the γ‐CH of NacNacLi to produce a three‐armed species. Meanwhile, compound **10** highlights the need for careful stoichiometric control of the reagents in such syntheses, to prevent formation of side products possibly by over‐lithiation. Due to their analogous nature to compounds **1** and **1 a** respectively, inserted ligand species **2**–**9**, as well as intermediates **2 a**, **3 a** and **7 a** have not been discussed here for brevity (check the SI for further details of them).

### N,N,N,S ligands

Multidentate NNS coordinating NacNac ligands have already been established in the literature. In general, access is via the introduction of a linked donor arm at the nitrogen position(s) containing a thiophene heterocycle. However, in some instances, the resulting tridentate ligands were found to adopt NNC coordination modes, instead of NNS coordination, due to the activation of one C−H bond within the thiophene ring.^[28]^ In an analogous manner to the generation of tripodal NacNac ligands with N,N,N,O coordination sites using isocyanate reagents, we have been able to generate tripodal NacNac ligands containing tetra‐heteroatomic N,N,N,S coordination sites using isothiocyanate insertion at the γ‐carbon position of NacNacLi.

**Figure 9:**
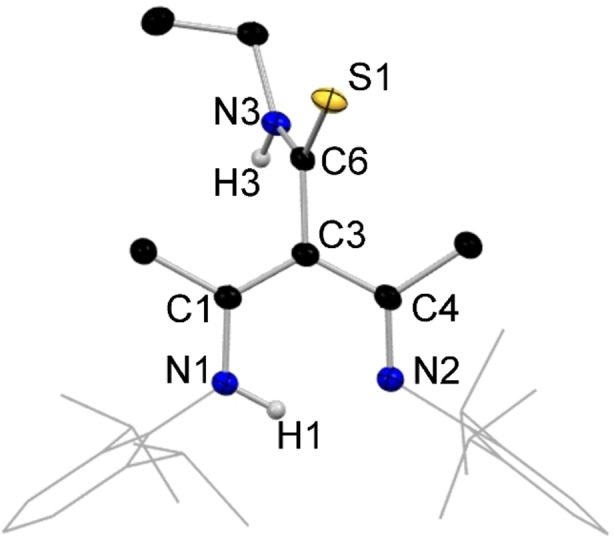
Molecular structure of **14**, representative of structures **11–14**, with H atoms omitted except for N−H, and Dipp groups shown as wire frame for clarity. Thermal ellipsoids are displayed at 40 % probability level.

The products of these isothiocyanate reactions follow the reaction shown in Scheme 1. Crystallographic determination of their structures reveals similar structural motifs for several variations of the organic group R [4‐MePh (**11**), ^t^Bu (**12**), Ph (**13**), Et (**14**)]. Representative of the proligand products characterised from these reactions is [(MeCNH‐Dipp)(MeCN‐Dipp)C(EtNHCS)], **14** (Figure 9). Using ethyl isothiocyanate, EtNCS, for the insertion, has yielded a tripodal molecule containing four heteroatoms, potentially capable of effecting NNN or NNS type coordination to a metal. Crystallographic data for **14** reveals two ‐NH sites available for deprotonation, with relatively localised bonding shown at the inserted −CSN− unit, [C6S1, 1.6859(14) Å, C6−N3, 1.3252(19) Å] compared to a typical −N=C=S unit [C=S, 1.587(3) Å].^[74]^

**Figure 10:**
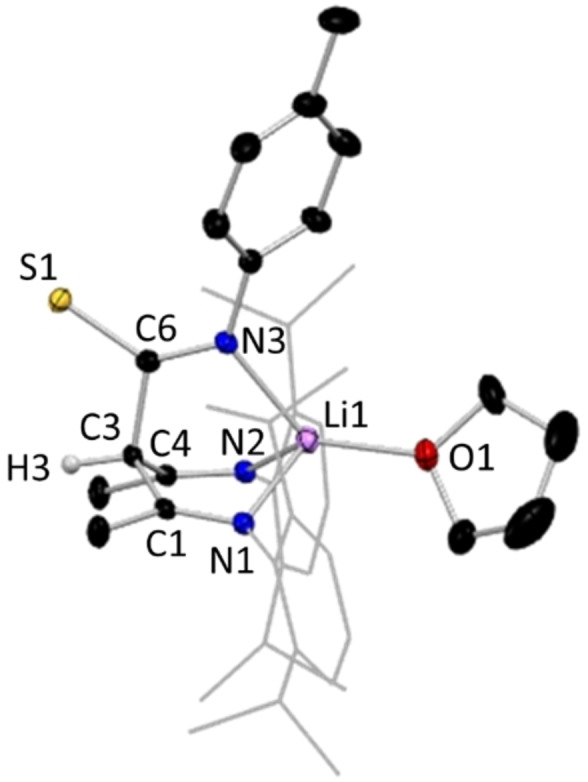
Molecular structure of **11 a**, with H atoms (except for C−H) and co‐crystallised solvent molecule omitted. Dipp groups shown as wire frame for clarity. Thermal ellipsoids are displayed at 40 % probability level.

**Scheme 3:**
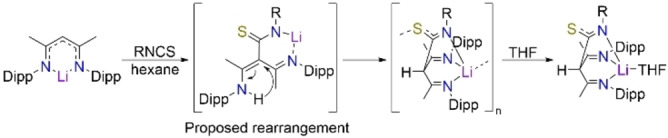
Proposed tautomerisation of ‐NH to ‐CH observed in lithium compounds **11 a**, **13 a**, and **14 a**.

It was possible that insertion of an isothiocyanate unit into the γ‐carbon position of NacNac, would generate a tripodal NacNac ligand with the potential to adopt an NNS coordination mode towards metals. However, in the structures of the monomeric lithium intermediates [{(MeCN‐Dipp)_2_CH(4‐MeC_6_H_4_NCS)}Li ⋅ THF], **11 a** (Figure 10) and [{(MeCN‐Dipp)_2_CH(PhNCS)}Li ⋅ 2THF] **13 a**, the isothiocyanate analogues of these new tripodal NacNac's, the NNN coordination mode is preferred over NNS, again aligned with HSAB theory, where ‘hard’ lithium bonds preferentially to hard nitrogen rather than to softer sulfur.[^18^, ^19^, ^20^] In **11 a**, the Li centre carries a single THF ligand, in contrast to the di‐solvates encountered in the monomeric N,N,N,O analogues (**1 a**, **2 a**, **3 a** and **7 a**), reflecting the higher tridentate ligation of lithium by the anion in **11 a**, compared to the bidentate ligation in N,N,N,O monomers. In **11 a**, Li binds to one O and three N atoms with bond angles over a range of 92.96(14)°–94.95(14)° for N−Li−N and 109.86(17)°–133.40(18)° for N−Li−O giving an overall mean bond angle of 107.91° (τ_4_=0.73).^[66]^ This tridentate coordination to Li is accompanied by tautomerisation of the −N*H* atom seen in the NNO lithiated species, and its relocation to the central carbon, C3, of the NacNac backbone forming a CH bond identified at 6.11 ppm in the ^1^H NMR spectrum (Scheme 3). Consequently, charge delocalisation occurs in the N−C−S unit of the isothiocyanate arm of **11 a**, compared to the S=C(−NH)− units of the isothiocyanate proligands **12** [S=C, 1.690(4) Å, C−NH, 1.329(3) Å) and **14** (S=C, 1.6859(14) Å, C−NH, 1.3252(19) Å].

**Figure 11:**
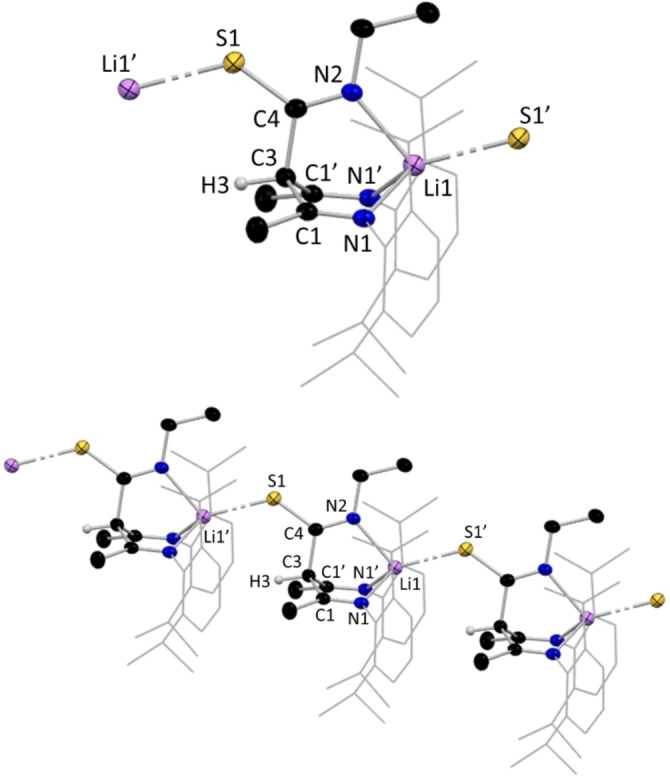
(Top) Molecular section of **14 a**; (bottom) section of polymeric **14 a**, with H atoms omitted except for C−H, and Dipp groups shown as wire frame for clarity. Thermal ellipsoids are displayed at 40 % probability level. Dashed line highlights Li−S propagation contacts.

Our studies have shown that N,N,N,O tripodal NacNacs can have at least three coordination modes for the lithiated species, while two coordination modes have been found for their N,N,N,S counterparts. Compound **14 a**, [{(MeCN‐Dipp)_2_CH(EtNCS)}Li], exemplifies the arrangement adopted in the absence of donor solvation, and an N,N,N,S coordination mode, in which lithium bonds to the sulfur atom of its neighbour to propagate a linear polymeric chain (Figure 11, bottom). Here, Li binds to three N atoms and the S atom of the adjacent repeating unit with a mean bond angle of 107.8° over a range of 91.7(2)°–127.66(19)° (τ_4_=0.74), indicating a similar pseudo trigonal pyramidal arrangement to that in **11 a**.^[66]^ As in **11 a**, the γ‐carbon position carries a hydrogen atom and is thus tetrahedral, with a diimine unit on each arm of the NacNac framework. A mirror plane cuts through C3, H3, C4, S1, N2, Li1 and the ethyl chain of **14 a**, resulting in equivalent bond lengths between each half of the molecule (see table 1).

**Figure 12:**
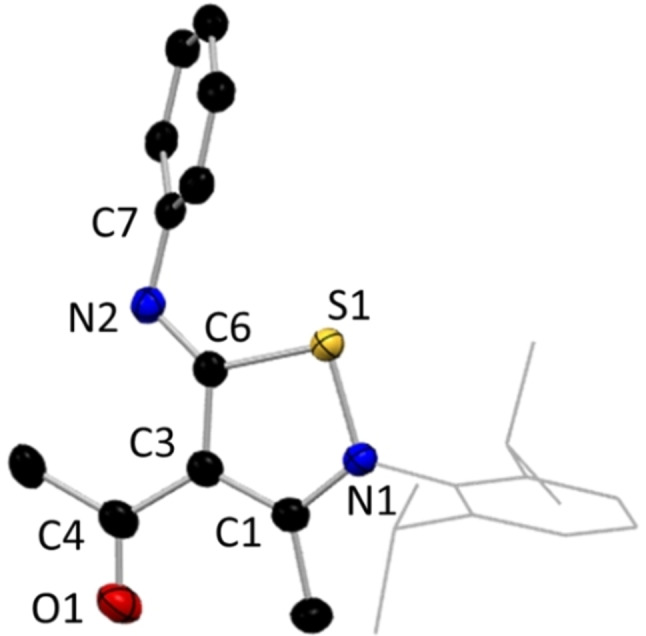
Molecular structure of **15**, with H atoms omitted, and the Dipp group shown as wire frame for clarity. Thermal ellipsoids are displayed at 40 % probability level.

**Scheme 4:**
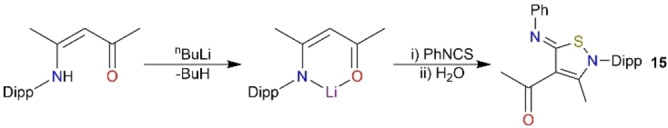
Synthesis of isthiocyanate inserted β‐ketiminate **15** from NacAc contaminant and phenylisothiocyanate.

Compound **15**, [(N‐Dipp)C(CH_3_)C(C(O)CH_3_)C(NPh)S] was isolated from reaction of NacNacLi with phenylisothiocyanate, PhNCS. From the structure of **15** (Figure 12), it can be assumed that the reaction solution was contaminated by trace amounts of “NacAc” present from the initial ligand synthesis (Scheme 4). β‐Ketoiminates, or NacAcs, represent the unsymmetrical hybrid of AcAc and NacNac ligands, where only one C=O site has been converted to an imine. NacAc species have proved useful ligands in their own right, capable of exhibiting N,O and C,N bidentate coordination, via the γ‐carbon position, and thus allowing formation of 6‐ or 4‐membered rings with metal centres.[^75^, ^76^, ^77^, ^78^] The molecular structure of metal‐free **15** exhibits a 5‐membered (SNCCC) heterocycle, created by formation of a bond between the sulfur atom of the inserted isothiocyanate and the nitrogen atom of the NacAc ligand. A consequence of this is the formation of a double bond in N2=C6 [1.261(3) Å], while the C=O bond of the NacAc unit remains untouched [C4=O1, 1.221(3) Å]. A few examples of compounds analogous to **15** have been reported in the literature, but to the best of our knowledge this ‐Dipp variant has not been characterised before now.[^79^, ^80^, ^81^, ^82^, ^83^] Compound **15** may be a fortuitous product in our reaction, but it proves that NacAc is capable of undergoing small molecule insertions at the γ‐carbon position akin to those of NacNac. This may be unsurprising given the ability of NacAc ligands to form 5‐membered rings via the γ‐carbon position, as precedented in the literature.^[75]^

In an analogous manner to the N,N,N,O type ligands discussed previously, the availability of both N and S heteroatoms in these tripodal N,N,N,S ligand species makes them potentially attractive candidates for further study and possibly catalyst development. For brevity, inserted ligand species **11**–**13**, as well as intermediate species **13 a** have not been discussed here, as they are structurally analogous to species already discussed herein (see the SI for more details of them).

### N,N,N,N ligands

**Scheme 5:**
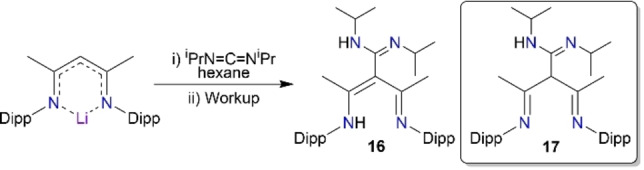
Synthesis of N,N,N,N type tripodal tautomers, **16** and **17**, from NacNacLi and diisopropylcarbodiimide (DIC).

**Figure 13:**
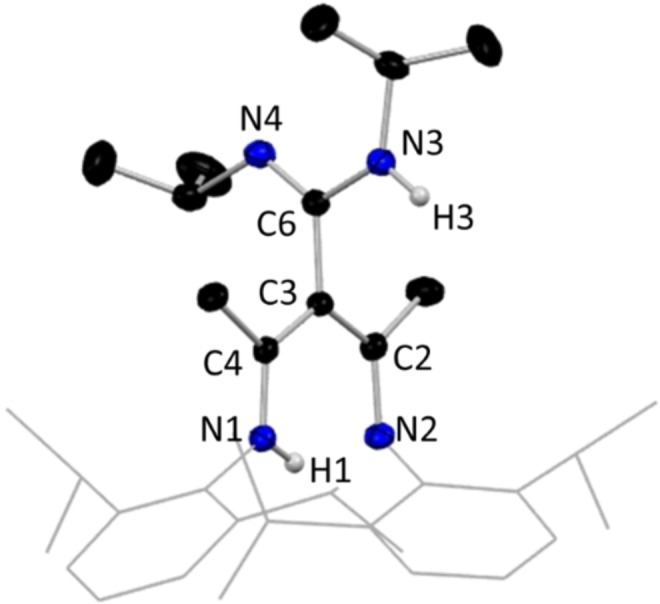
Molecular structure of **16**, with H atoms omitted except for N−H, and Dipp groups shown as wire frame for clarity. Thermal ellipsoids are displayed at 40 % probability level.

Following the isocyanates and isothiocyanates study, we next probed the reactivity of a carbodiimide species, which is isoelectronic to these other small molecules. Thus, reaction of NacNacLi with diisopropylcarbodiimide (DIC, Scheme 5) produced an N,N,N,N type ligand with up to four heteroatom sites for possible metal coordination, as witnessed in the crystal structure of the product [(MeCNH‐Dipp)(MeCN‐Dipp)C(NH‐^i^PrCN‐^i^Pr)], **16** (Figure 13).

**Figure 14:**
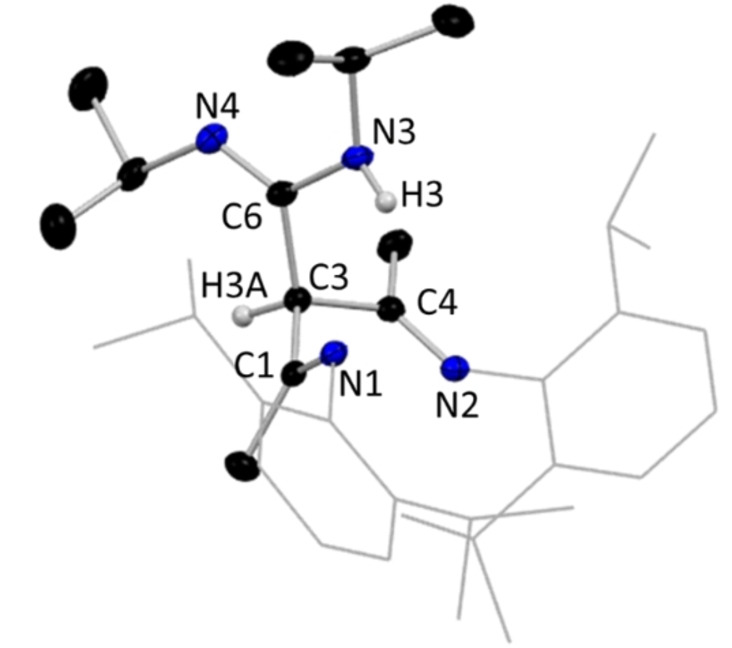
Molecular structure of compound **17**, with H atoms omitted except for N−H and *γ*‐C−H, and Dipp groups shown as wire frame for clarity. Thermal ellipsoids are displayed at 40 % probability level.

In this structure, the NacNac pincers have regained a hydrogen atom, located between the two nitrogen sites, hence, the central NacNac unit remains planar [N1C4 1.3192(17) Å, C4C3 1.4204(17) Å, C3C2 1.4113(17) Å, C2N2 1.3359(17) Å]. Bonding in the inserted carbodiimide‐derived arm is strongly localised with single and double bond character respectively in C6−N3 [1.3721(17) Å] and C6−N4 [1.2821(17) Å]. Like the previous NNO ligands discussed, γ‐carbon C3 is devoid of a hydrogen atom, so it shows distorted trigonal planar coordination. An interesting feature of this reaction is that the resulting ligand was also captured in an alternative tautomeric form, in [(MeCN‐Dipp)_2_CH(NH‐^i^PrCN‐^i^Pr)], **17** (Figure 14) under seemingly identical reaction conditions. In marked contrast to tautomer **16**, the diimine form, **17**, does not have a planar NacNac backbone, instead we see a marked disruption in the NCCCN plane (N1−C1−C4−N2 dihedral angle, 64.7°) with the absence of a shared H atom between the central N pincers, and with the H atom now sited at the now tetrahedral γ‐C centre. From a comparison of the reaction producing compound **2**, this DIC reaction could be the result of a similar excess of organolithium reagent, though this time without an additional insertion into the new tripodal ligand arm. To date only **16** has proved reproducible in a 53 % yield, while a small amount of **17** was only ever isolated from the original reaction. Seemingly identical conditions were used in the synthesis of **16** and **17** and thus the reason behind the isolation of different tautomers as the crystalline product remains an open question.

**Scheme 6:**
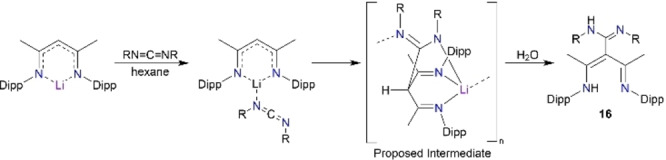
Proposed route for the formation of compound **16**.

In **17**, the NacNac diimine unit is highly localised, [N1=C1, 1.2731(19) Å; C1−C3, 1.524(2) Å; C3−C4, 1.521(2) Å; C4=N2, 1.2703(19) Å; C3−C6, 1.5340(19) Å; C6−N3, 1.3793(19) Å; C6=N4, 1.2767(19) Å], and as usual for this diamine arrangement the hydrogen atom at C3 occupies a distorted tetrahedral geometry. While this NacNacLi DIC reaction gives insertion into the γ‐carbon position, another reaction of NacNacLi and a carbodiimide has previously been explored by the Mulvey group. In that case, insertion did not occur but instead gave the pre‐insertion intermediate, [{(MeCN‐Dipp)_2_CH}Li⋅(N−CyCN−Cy)]. This is a simple donor‐acceptor complex, providing a rare crystallographically verified example of a carbodiimide acting as a Lewis donor (shown in Scheme 6). Such a donor‐acceptor complex could be viewed as a model intermediate in the formation of an amidinate from a carbodiimide and an alkali metal nucleophilic source.^[46]^ The insertion of a diimine species into the γ‐carbon position of NacNac is not unprecedented, as it was crystallographically verified in [(CH{C(Me)NDipp}_2_(*p‐*CH_3_C_6_H_4_N)_2_C)Mg{(*p‐*CH_3_C_6_H_4_N)_2_C(SiMe_2_Ph)}] by the Hill group.^[84]^

## Conclusions

In this exploratory study we endeavoured to turn an unwanted reaction, namely involvement of the γ‐C−H unit in the backbone of the “innocent” NacNac ligand, into a wanted reaction that could yield three‐armed modifications of NacNac. Treating NacNacLi with isocyanate, isothiocyanate, and carbodiimide small molecules to deliberately induce insertion of the unsaturated group into this C−H bond was the approach used. Overall, this proposal was successful through the synthesis of 15 modified NacNac compounds as well as 10 NacNacLi intermediates, albeit some were obtained in poor yields and/or in complicated mixtures. X‐ray crystallographic studies determined both the structures of these modified NacNac compounds and their lithiated precursors, with the latter confirming the ligating nature of these new tripodal NacNac variants. Future studies will focus on taking those tripodal proligands that can be made in high purity and yield to develop metal derivatives of them or alternatively using the lithiated NacNac compounds in transmetallation reactions, with the aim in each case of seeking potential new catalysts containing novel ligands for homogeneous organic transformations.

## Experimental Section

### General Experimental Procedures

All reactions were performed under a protective argon or nitrogen atmosphere using standard glovebox or Schlenk techniques respectively. All solvents used, other than deionised water, were dried prior to synthesis by solvent purification system (Innovative Technologies SPS PS‐Micro) to remove any traces of moisture and dissolved oxygen before transfer to a J Young's ampoule and subsequent storage over 4 Å molecular sieves.

C_6_D_6_ was degassed by freeze‐pump‐thaw methods and stored over activated 4 Å molecular sieves. CDCl_3_ was stored over activated 4 Å molecular sieves. ^n^BuLi (1.6 M in hexane) and LiCH_2_Si(CH_3_)_3_ (1.0 M in pentane) were purchased commercially from Sigma‐Aldrich, ^n^BuLi was used as received, while LiCH_2_Si(CH_3_)_3_ was dried in vacuo and stored at −20 °C. The parent NacNac(H) ligand and lithium derivative, NacNacLi, were made *via* literature procedures.^[1]^ Isocyanate, isothiocyanate and carbodiimide reagents were purchased from Sigma‐Aldrich and stored over activated 4 Å molecular sieves before use. All other reagents were purchased from commercial sources and were also used as received.

NMR spectra were recorded on a Bruker AV400 MHz spectrometer operating 400.13 MHz for ^1^H, 155.47 MHz for ^7^Li and 100.62 MHz for ^13^C. All ^13^C NMR spectra were proton decoupled. ^1^H, ^13^C{^1^H} and ^7^Li chemical shifts are expressed in parts per million (δ, ppm) and where appropriate referenced to residual solvent peaks or external references.

Crystallographic data for complexes **1**–**17** were collected on an Oxford Diffraction Synergy instrument with monochromated Cu Kα (λ 1.54184 Å) radiation. Data collection and processing used CrysalisPro software.^[2]^ All structures were solved and refined to convergence on *F* ^2^ against all independent reflections by the full‐matrix least squares method using SHELXL^[3]^ as implemented within OLEX2.^[4]^

**General Synthesis of Proligands**. NacNacLi (0.850 g, 2.0 mmol) dissolved in hexane (10 ml) produced a yellow solution before RN=C=X (2.0 mmol) was added to give an off‐white/yellow suspension (where R represents the organic group and X represents either NR, O or S). The suspension was stirred (for 3 h) before deionised water (5 ml) was added, then the mixture was exposed to air and stirred overnight. Next, separation was performed using DCM (15 ml), the organic layer dried using MgSO_4_ before the product was filtered, dried, and crystallised from methanol. See SI for further details.

**General Synthesis of Lithium Complexes**. NacNacLi (0.213 g, 0.5 mmol) was dissolved in hexane (5 ml), RN=C=X (0.5 mmol) added to form a suspension (where R represents the organic group and X represents either O or S). THF (2 ml) added to regain solution and a crop of pale‐yellow crystals grew upon slow evaporation of solvent in −20 °C freezer. See SI for further details.

## Supporting Information

Supporting Information contains experimental methods, crystallographic information, NMR spectra, IR data and melting point data (PDF). A Data set containing the raw crystallographic and X‐ray data (cif, fid) can be located at the joint Cambridge Crystallographic Data Centre and Fachinformationszentrum Karlsruhe Access Structures service www.ccdc.cam.ac.uk/structures (deposition numbers 2298370–2298394). The authors have cited additional references within the Experimental Section and Supporting Information.

## Conflict of interests

The authors declare no conflict of interest.

1

## Supporting information

As a service to our authors and readers, this journal provides supporting information supplied by the authors. Such materials are peer reviewed and may be re‐organized for online delivery, but are not copy‐edited or typeset. Technical support issues arising from supporting information (other than missing files) should be addressed to the authors.

Supporting Information

## Data Availability

The data that support the findings of this study are openly available in PUREportal.strath.ac.uk at https://doi.org/10.15129/4e953715‐e990‐4509‐8684‐86cb783d9ef6, reference number 175240789.

## References

[1] L. Bourget-Merle , M. F. Lappert , J. R. Severn , Chem. Rev. 2002, 102, 3031–3066. 12222981 10.1021/cr010424r

[2] C. Camp , J. Arnold , Dalton Trans. 2016, 45, 14462–14498. 27353604 10.1039/c6dt02013e

[3] S. Brand , J. Pahl , H. Elsen , S. Harder , Eur. J. Inorg. Chem. 2017, 4187–4195.

[4] S. K. Mandal , H. W. Roesky , Acc. Chem. Res. 2012, 45, 298–307. 21882810 10.1021/ar2001759

[5] R. L. Webster , Dalton Trans. 2017, 46, 4483–4498. 28262881 10.1039/c7dt00319f

[6] A. Stasch , C. Jones , Dalton Trans. 2011, 40, 5659–5672. 21390353 10.1039/c0dt01831g

[7] C. Jones , Nat. Chem. Rev. 2017, 1, 0059.

[8] T. X. Gentner , B. Rösch , G. Ballmann , J. Langer , H. Elsen , S. Harder , Angew. Chem. Int. Ed. 2019, 58, 607–611. 10.1002/anie.201812051 30422354

[9] B. Rösch , T. X. Gentner , J. Eyselein , J. Langer , H. Elsen , S. Harder , Nature 2021, 592, 717–721. 33911274 10.1038/s41586-021-03401-w

[10] C. Cui , H. W. Roesky , H. G. Schmidt , M. Noltemeyer , H. Hao , F. Cimpoesu , Angew. Chem. Int. Ed. 2000, 39, 4274–4276. 10.1002/1521-3773(20001201)39:23<4274::AID-ANIE4274>3.0.CO;2-K 29711904

[11] S. Grams , J. Mai , J. Langer , S. Harder , Organometallics 2022, 41, 2862–2867.

[12] M. Zhong , S. Sinhababu , H. W. Roesky , Dalton Trans. 2020, 49, 1351–1364. 31942579 10.1039/c9dt04763h

[13] S. Brand , H. Elsen , J. Langer , S. Grams , S. Harder , Angew. Chem. Int. Ed. 2019, 58, 15496–15503. 10.1002/anie.201908978 PMC6856855 31465144

[14] N. J. Hardman , B. E. Eichler , P. P. Power , Chem. Commun. 2000, 1991–1992.

[15] W. D. Woodul , E. Carter , R. Müller , A. F. Richards , A. Stasch , M. Kaupp , D. M. Murphy , M. Driess , C. Jones , J. Am. Chem. Soc. 2011, 133, 10074–10077. 21662245 10.1021/ja204344e

[16] M. H. Chisholm , J. C. Gallucci , K. Phomphrai , Inorg. Chem. 2005, 44, 8004–8010. 16241150 10.1021/ic048363d

[17] S. Ghosh , P. M. Schäfer , D. Dittrich , C. Scheiper , P. Steiniger , G. Fink , A. N. Ksiazkiewicz , A. Tjaberings , C. Wölper , A. H. Gröschel , A. Pich , S. Herres-Pawlis , S. Schulz , ChemistryOpen 2019, 8, 951–960. 31338277 10.1002/open.201900203 PMC6625107

[18] R. G. Pearson , J. Am. Chem. Soc. 1963, 85, 3533–3539.

[19] R. G. Pearson , J. Chem. Educ. 1968, 45, 581–587.

[20] R. G. Pearson , J. Chem. Educ. 1968, 45, 643–648.

[21] P. Buchwalter , J. Rosé , P. Braunstein , Chem. Rev. 2015, 115, 28–126. 25545815 10.1021/cr500208k

[22] J. A. Mata , F. E. Hahn , E. Peris , Chem. Sci. 2014, 5, 1723–1732.

[23] S. D. Robertson , M. Uzelac , R. E. Mulvey , Chem. Rev. 2019, 119, 8332–8405. 30888154 10.1021/acs.chemrev.9b00047

[24] V. A. Pollard , S. A. Orr , R. McLellan , A. R. Kennedy , E. Hevia , R. E. Mulvey , Chem. Commun. 2018, 54, 1233–1236. 10.1039/c7cc08214b 29336450

[25] D. R. Armstrong , A. R. Kennedy , R. E. Mulvey , S. D. Robertson , Chem. Eur. J. 2011, 17, 8820–8831. 21766365 10.1002/chem.201101167 PMC3761191

[26] M. Uzelac , R. E. Mulvey , Chem. Eur. J. 2018, 24, 7786–7793. 29603459 10.1002/chem.201800489

[27] T. X. Gentner , R. E. Mulvey , Angew. Chem. Int. Ed. 2021, 60, 9247–9262. 10.1002/anie.202010963 PMC8247348 33017511

[28] X. Zhu , Z. Wang , L. Zha , Y. Zhang , Y. Qi , Q. Yuan , S. Zhou , S. Wang , Organometallics 2022, 41, 1087–1098.

[29] S. Pahar , V. S. V. S. N. Swamy , T. Das , R. G. Gonnade , K. Vanka , S. S. Sen , Chem. Commun. 2020, 56, 11871–11874. 10.1039/d0cc05202g 33021295

[30] E. Lu , W. Gan , Y. Chen , Organometallics 2009, 28, 2318–2324.

[31] X. Xu , X. Xu , Y. Chen , J. Sun , Organometallics 2008, 27, 758–763.

[32] H. Feng , D. Yang , T. Mei , Y. Zhang , B. Wang , J. Qu , Eur. J. Inorg. Chem. 2022, e202200290.

[33] H. Gao , J. Su , P. Xu , X. Xu , Org. Chem. Front. 2018, 5, 59–63.

[34] R. Olejník , Z. Padělková , A. Fridrichová , M. Horáček , J. Merna , A. Růžička , J. Organomet. Chem. 2014, 759, 1–10.

[35] S. M. Barbon , V. N. Staroverov , P. D. Boyle , J. B. Gilroy , Dalton Trans. 2014, 43, 240–250. 24096386 10.1039/c3dt52188e

[36] G. B. Nikiforov , H. W. Roesky , T. Labahn , D. Vidovic , D. Neculai , Eur. J. Inorg. Chem. 2003, 433–436.

[37] T. Kurogi , J. Chu , Y. Chen , D. J. Mindiola , Chem. Asian J. 2019, 14, 2629–2638. 31233290 10.1002/asia.201900451

[38] S. Pfirrmann , C. Limberg , E. Hoppe , Z. Anorg. Allg. Chem. 2009, 635, 312–316.

[39] C. Zovko , S. Bestgen , C. Schoo , A. Görner , J. M. Goicoechea , P. W. Roesky , Chem. Eur. J. 2020, 26, 13191–13202. 32285968 10.1002/chem.202001357 PMC7693294

[40] E. E. Marlier , C. M. Seong , S. A. Brunclik , M. H. Nevins , E. L. Nolan , A. K. Olson , M. Osnaya , A. Reuter , M. E. Swanson , O. G. H. Wood , D. E. Janzen , Polyhedron 2021, 201, 115150.

[41] M. L. Scheuermann , A. T. Luedtke , S. K. Hanson , U. Fekl , W. Kaminsky , K. I. Goldberg , Organometallics 2013, 32, 4752–4758.

[42] M. D. Anker , M. Arrowsmith , P. Bellham , M. S. Hill , G. Kociok-Köhn , D. J. Liptrot , M. F. Mahon , C. Weetman , Chem. Sci. 2014, 5, 2826–2830. 10.1039/c5sc03114a PMC5952893 29896350

[43] R. M. Gauld , R. McLellan , A. R. Kennedy , J. Barker , J. Reid , R. E. Mulvey , Chem. Eur. J. 2019, 25, 14728–14734. 31574177 10.1002/chem.201904013

[44] A. R. Cabrera , Y. Schneider , M. Valderrama , R. Fröhlich , G. Kehr , G. Erker , R. S. Rojas , Organometallics 2010, 29, 6104–6110.

[45] K. Spannhoff , R. Rojas , R. Fröhlich , G. Kehr , G. Erker , Organometallics 2011, 30, 2377–2384.

[46] R. M. Gauld , J. R. Lynch , A. R. Kennedy , J. Barker , J. Reid , R. E. Mulvey , Inorg. Chem. 2021, 60, 6057–6064. 33830739 10.1021/acs.inorgchem.1c00549 PMC8154426

[47] B. Li , C. Wölper , G. Haberhauer , S. Schulz , Angew. Chem. Int. Ed. 2021, 60, 1986–1991. 10.1002/anie.202012595 PMC7894565 33034935

[48] W. Ren , S. Zhang , Z. Xu , X. Ma , Dalton Trans. 2019, 48, 3109–3115. 30768120 10.1039/c9dt00090a

[49] C. E. Radzewich , M. P. Coles , R. F. Jordan , J. Am. Chem. Soc. 1998, 120, 9384–9385.

[50] T. E. Stennett , H. S. Zijlstra , F. W. Seidel , S. Harder , Organometallics 2016, 35, 207–217.

[51] J. Pahl , T. E. Stennett , M. Volland , D. M. Guldi , S. Harder , Chem. Eur. J. 2019, 25, 2025–2034. 30431191 10.1002/chem.201804802

[52] L. A. M. Harris , E. C. Y. Tam , M. P. Coles , J. R. Fulton , Dalton Trans. 2014, 43, 13803–13814. 25109774 10.1039/c4dt01714e

[53] J. R. Lynch , A. R. Kennedy , J. Barker , J. Reid , R. E. Mulvey , Helv. Chim. Acta 2022, 105, e202200082.

[54] A. Ziółkowska , N. Szynkiewicz , Ł. Ponikiewski , Organometallics 2019, 38, 2873–2877.

[55] S. P. Sarish , P. P. Samuel , H. W. Roesky , C. Schulzke , K. Nijesh , S. De , P. Parameswaran , Chem. Eur. J. 2015, 21, 19041–19047. 26593152 10.1002/chem.201503137

[56] L. J. Ball , A. P. Dickie , F. S. Mair , D. A. Middleton , R. G. Pritchard , Chem. Commun. 2003, 744–745. 10.1039/b211745b 12703802

[57] A. J. Boutland , I. Pernik , A. Stasch , C. Jones , Chem. Eur. J. 2015, 21, 15749–15758. 26358928 10.1002/chem.201502755

[58] F. Basuli , J. C. Huffman , D. J. Mindiola , Inorg. Chem. 2003, 42, 8003–8010. 14632519 10.1021/ic034853e

[59] N. Gorgas , A. J. P. White , M. R. Crimmin , Chem. Commun. 2022, 58, 10849–10852. 10.1039/d2cc04498f PMC9514013 36073319

[60] J. Schoening , C. Wölper , S. Schulz , Eur. J. Inorg. Chem. 2022, 26, e202200638.

[61] T. J. J. Whitehorne , F. Schaper , Inorg. Chem. 2013, 52, 13612–13622. 24245876 10.1021/ic402133c

[62] G. G. Skvortsov , A. V. Cherkasov , A. A. Trifonov , Russ. Chem. Bull. 2017, 66, 1665–1674.

[63] J. Cullinane , A. Jolleys , F. S. Mair , Dalton Trans. 2013, 42, 11971. 23835493 10.1039/c3dt51233a

[64] G. G. Skvortsov , G. K. Fukin , A. V. Cherkasov , T. A. Kovylina , A. A. Trifonov , Inorg. Chim. Acta 2020, 508, 119623.

[65] M. Stender , R. J. Wright , B. E. Eichler , J. Prust , M. M. Olmstead , H. W. Roesky , P. P. Power , J. Chem. Soc. Dalton Trans. 2001, 3465–3469.

[66] L. Yang , D. R. Powell , R. P. Houser , Dalton Trans. 2007, 955–964. 17308676 10.1039/b617136b

[67] M. C. Etter , J. C. MacDonald , J. Bernstein , Acta Crystallogr. Sect. B 1990, 46, 256–262. 2344397 10.1107/s0108768189012929

[68] R. M. Gauld , A. R. Kennedy , R. McLellan , J. Barker , J. Reid , R. E. Mulvey , Chem. Commun. 2019, 55, 1478–1481. 10.1039/c8cc08308h 30644951

[69] T. Maetzke , D. Seebach , Organometallics 1990, 9, 3032–3037.

[70] D. R. Armstrong , J. E. Davies , R. P. Davies , P. R. Raithby , R. Snaith , A. E. H. Wheatley , New J. Chem. 1999, 23, 35–41.

[71] J. E. Davies , P. R. Raithby , R. Snaith , A. E. H. Wheatley , Chem. Commun. 1997, 1721–1722.

[72] W. N. Setzer , P. V. R. Schleyer , in Adv. Organomet. Chem. 1985, pp. 353–451.

[73] S. C. Watson , J. F. Eastham , J. Organomet. Chem. 1967, 9, 165–168.

[74] P. Majewska , M. Rospenk , B. Czarnik-Matusewicz , A. Kochel , L. Sobczyk , R. Dąbrowski , Chem. Phys. 2008, 354, 186–195.

[75] D. C. H. Do , H. V. Huynh , Inorg. Chem. 2022, 61, 20087–20094. 36419368 10.1021/acs.inorgchem.2c03515

[76] T. S. Ouattara , R. J. Butcher , J. S. Matthews , J. Coord. Chem. 2005, 58, 461–465.

[77] H. Zhang , X. Geng , Z. Li , Y. Peng , L. Wang , P. Chen , Inorg. Chim. Acta 2023, 545, 121253.

[78] W. H. Monillas , G. P. A. Yap , K. H. Theopold , J. Chem. Crystallogr. 2009, 39, 380–383.

[79] J. Goerdeler , J. Gnad , Chem. Ber. 1965, 98, 1531–1543.

[80] B. Zaleska , D. Ciez , A. Haas , Synth. Commun. 1996, 26, 4165–4173.

[81] D. R. Dauer , D. Stalke , Dalton Trans. 2014, 43, 14432–14439. 24995935 10.1039/c4dt01008f

[82] J. Kretsch , I. Koehne , M. Lõkov , I. Leito , D. Stalke , Eur. J. Inorg. Chem. 2019, 3258–3264.

[83] J. Kretsch , A. Kreyenschmidt , T. Schillmöller , R. Herbst-Irmer , D. Stalke , Inorg. Chem. 2020, 59, 13690–13699. 32897060 10.1021/acs.inorgchem.0c02066

[84] B. Okokhere-Edeghoghon , M. Dehmel , M. S. Hill , R. Kretschmer , M. F. Mahon , C. L. McMullin , L. J. Morris , N. A. Rajabi , Inorg. Chem. 2020, 59, 13679–13689. 32886501 10.1021/acs.inorgchem.0c02034

[85] M. Stender , R. J. Wright , B. E. Eichler , J. Prust , M. M. Olmstead , H. W. Roesky , P. P. Power , J. Chem. Soc. Dalton Trans. 2001, 3465–3469.

[86] *Crysalis Pro*, Agilent Technologies Ltd., Yarnton, Oxfordshire, UK, **2014**.

[87] G. M. Sheldrick , Acta Crystallogr. Sect. A 2015, 71, 3–8. 10.1107/S2053273314026370 PMC4283466 25537383

[88] O. V. Dolomanov , L. J. Bourhis , R. J. Gildea , J. A. K. Howard , H. Puschmann , J. Appl. Crystallogr. 2009, 42, 339–341. 10.1107/S0021889811041161 PMC3236671 22199401

[89] L. J. Farrougia , J. Appl. Crystallogr. 2012, 45, 849–854.

[90] R. M. Gauld , R. McLellan , A. R. Kennedy , F. J. Carson , J. Barker , J. Reid , C. T. O'Hara , R. E. Mulvey , Eur. J. Inorg. Chem. 2021, 1615–1622.

