# Supplementary material for: Modification of a Common β‐diketiminate NacNac Framework via Sequential Lithiation and Small Molecule Insertion
Source: Chemistry. 2024 Jan 26;30(15):e202303373. doi: 10.1002/chem.202303373 (PMC11497280; doi:10.1002/chem.202303373)
Supplement: Supplementary file 1 — Supporting Information [file CHEM-30-e202303373-s001.pdf]

Chemistry–A European Journal

Supporting Information

# Modification of a Common β-diketiminate NacNac Framework via Sequential Lithiation and Small Molecule Insertion

Jennifer R. Lynch, Alan R. Kennedy, Jim Barker, and Robert E. Mulvey*

# Supporting Information to accompany

# Modification of a Common β-diketiminate NacNac Framework via Sequential Lithiation and Small Molecule Insertion

Jennifer R. Lynch,[a] Dr. Alan R. Kennedy,[a] Dr. Jim Barker,[b] and Prof. Robert E. Mulvey*[a]

a. Department of Pure and Applied Chemistry, University of Strathclyde, Glasgow, G1 1XL, UK.

b. Innospec Ltd., Innospec Manufacturing Park, Oil Sites Road, Ellesmere Port, Cheshire, CH65 4EY, UK.

e-mail: r.e.mulvey@strath.ac.uk

# Contents


General Experimental....5

X-ray crystallography....6

Synthesis of [(MeCNH-Dipp)(MeCN-Dipp)C($^{t}$BuNHCO)] (**1**)....7

Figure S1: ORTEP diagram of [(MeCNH-Dipp)(MeCN-Dipp)C($^{t}$BuNHCO)] (**1**)....8

Table S1: Selected bond lengths (Å) and bond angles (°) of **1**....8

Synthesis of [{(MeCN-Dipp)$_2$C($^{t}$BuNHCO)}Li·2THF] (**1a**)....10

Figure S1a: ORTEP diagram of [{(MeCN-Dipp)$_2$C($^{t}$BuNHCO)}Li·2THF] (**1a**)....11

Table S2: Selected bond lengths (Å) and bond angles (°) of **1a**....12

Synthesis of [(MeCNH-Dipp)(MeCN-Dipp)C(4-OMeC$_6$H$_4$NHCO)] (**2**)....15

Synthesis of [{(MeCN-Dipp)$_2$C(4-OMeC$_6$H$_4$NHCO)}Li·2THF] (**2a**)....17

Figure S2a: ORTEP diagram of [{(MeCN-Dipp)2C(4-OMeC6H4NHCO)}Li·2THF] (**2a**)....18

Table S3: Selected bond lengths (Å) and bond angles (°) of **2a**....19

Synthesis of [(MeCNH-Dipp)(MeCN-Dipp)C(4-MeC$_6$H$_4$NHCO)] (**3**)....22

Figure S3: ORTEP diagram of [(MeCNH-Dipp)(MeCN-Dipp)C(4-MeC$_6$H$_4$NHCO)] (**3**)....23

Table S4: Selected bond lengths (Å) and bond angles (°) of **3**....23

Synthesis of [{(MeCN-Dipp)$_2$C(4-MeC$_6$H$_4$NHCO)}Li·2THF](**3a**)....25

Figure S3a: ORTEP diagram of [{(MeCN-Dipp)$_2$C(4-MeC$_6$H$_4$NHCO)}Li·2THF](**3a**)....26

Table S5: Selected bond lengths (Å) and bond angles (°) of **3a**....27

Synthesis of [(MeCNH-Dipp)(MeCN-Dipp)C(2-MeC$_6$H$_4$NHCO)] (**4**)....30

Figure S4: ORTEP diagram of [(MeCNH-Dipp)(MeCN-Dipp)C(2-MeC$_6$H$_4$NHCO)] (**4**)....31

Table S6: Selected bond lengths (Å) and bond angles (°) of **4**....31

Synthesis of [(MeCNH-Dipp)(MeCN-Dipp)C(2,6-Me$_2$C$_6$H$_3$NHCO)] (**5**)....33

Figure S5: ORTEP diagram of [(MeCNH-Dipp)(MeCN-Dipp)C(2,6-Me$_2$C$_6$H$_3$NHCO)] (**5**)....34

Table S7: Selected bond lengths (Å) and bond angles (°) of **5**....34

Synthesis of [(MeCNH-Dipp)(MeCN-Dipp)C(CyNHCO)] (**6**)....36

Figure S6: ORTEP diagram of [(MeCNH-Dipp)(MeCN-Dipp)C(CyNHCO)] (**6**)....37

Synthesis of [(MeCNH-Dipp)(MeCN-Dipp)C(PhNHCO)] (**7**)....39

Figure S7: ORTEP diagram of [(MeCNH-Dipp)(MeCN-Dipp)C(PhNHCO)] (**7**)....40

Table S8: Selected bond lengths (Å) and bond angles (°) of **7**....40

Synthesis of [{(MeCN-Dipp)$_2$C(4-MeC$_6$H$_4$NHCO)}Li·2THF] (**7a**)....43

Figure S7a: ORTEP diagram of [{(MeCN-Dipp)$_2$C(4-MeC$_6$H$_4$NHCO)}Li·2THF] (**7a**)....44

Table S9: Selected bond lengths (Å) and bond angles (°) of **7a** ........ 45
Synthesis of [{(MeCN-Dipp)(MeCNH-Dipp)C(PhNCO)}Li]$_4$·2THF (**7b**) ........ 48
Figure S7b: ORTEP diagram of [{(MeCN-Dipp)(MeCNH-Dipp)C(PhNCO)}Li]$_4$·2THF (**7b**) ........ 49
Table S10: Selected bond lengths (Å) and bond angles (°) of **7b** ........ 50
Synthesis of [(MeCNH-Dipp)(MeCN-Dipp)C($^i$PrNHCO)] (**8**) ........ 53
Figure S8: ORTEP diagram of [(MeCNH-Dipp)(MeCN-Dipp)C($^i$PrNHCO)] (**8**) ........ 54
Table S11: Selected bond lengths (Å) and bond angles (°) of **8** ........ 54
Synthesis of [{(MeCN-Dipp)$_2$C($^i$PrNHCO)}Li·THF]$_2$ (**8a**) ........ 56
Figure S8a: ORTEP diagram of [{(MeCN-Dipp)$_2$C($^i$PrNHCO)}Li.THF]$_2$ (**8a**) ........ 57
Synthesis of [(MeCNH-Dipp)(MeCN-Dipp)C(EtNHCO)] (**9**) ........ 60
Figure S9: ORTEP diagram of [(MeCNH-Dipp)(MeCN-Dipp)C(EtNHCO)] (**9**) ........ 61
Table S12: Selected bond lengths (Å) and bond angles (°) of **9** ........ 61
Synthesis of [{(MeCN-Dipp)$_2$C(EtNHCO)}Li·THF]$_2$ (**9a**) ........ 63
Figure S9a: ORTEP diagram of [{(MeCN-Dipp)$_2$C(EtNHCO)}Li·THF]$_2$ (**9a**) ........ 64
Table S13: Selected bond lengths (Å) and bond angles (°) of **9a** ........ 65
Synthesis of [(MeCNH-Dipp)(MeCN-Dipp)C{C(O)N($C_6H_4$OMe)C(O)N(H)$C_6H_4$OMe}] (**10**) ........ 68
Figure S10: ORTEP diagram of [(MeCNH-Dipp)(MeCN-Dipp)C{C(O)N($C_6H_4$OMe)C(O)N(H)$C_6H_4$OMe}] (**10**) ........ 69
Table S14: Selected bond lengths (Å) and bond angles (°) of **10** ........ 70
Synthesis of [(MeCNH-Dipp)(MeCN-Dipp)C(4-Me$C_6H_4$NHCS)] (**11**) ........ 72
Figure S11: ORTEP diagram of [(MeCNH-Dipp)(MeCN-Dipp)C(4-Me$C_6H_4$NHCS)] (**11**) ........ 73
Synthesis of [{(MeCN-Dipp)$_2$CH(4-Me$C_6H_4$NHCS)}Li·THF] (**11a**) ........ 75
Table S15: Selected bond lengths (Å) and bond angles (°) of **11a** ........ 76
Synthesis of [(MeCNH-Dipp)(MeCN-Dipp)C($^t$BuNHCS)] (**12**) ........ 79
Figure S12: ORTEP diagram of [(MeCNH-Dipp)(MeCN-Dipp)C($^t$BuNHCS)] (**12**) ........ 80
Table S16: Selected bond lengths (Å) and bond angles (°) of **12** ........ 80
Synthesis of [(MeCNH-Dipp)(MeCN-Dipp)C(PhNHCS)] (**13**) ........ 82
Synthesis of [{(MeCN-Dipp)$_2$CH(PhNHCS)}Li·THF] (**13a**) ........ 84
Figure S13a: ORTEP diagram of [{(MeCN-Dipp)$_2$CH(PhNHCS)}Li·THF] (**13a**) ........ 85
Table S17: Selected bond lengths (Å) and bond angles (°) of **13a** ........ 85
Synthesis of [(MeCNH-Dipp)(MeCN-Dipp)C(EtNHCS)] (**14**) ........ 88
Figure S14: ORTEP diagram of [(MeCNH-Dipp)(MeCN-Dipp)C(EtNHCS)] (**14**) ........ 89
Table S18: Selected bond lengths (Å) and bond angles (°) of **14** ........ 89
Synthesis of [{(MeCN-Dipp)$_2$CH(EtNCS)}Li] (**14a**) ........ 91

Figure S14a: ORTEP diagram of [{(MeCN-Dipp)$_2$CH(EtNCS)}Li] (**14a**) ........ 92
Table S19: Selected bond lengths (Å) and bond angles (°) of **14a** ........ 92
Synthesis of [(N-Dipp)C(CH$_3$)C(C(O)CH$_3$)C(NPh)S] (**15**) ........ 95
Figure S15: ORTEP diagram of [(N-Dipp)C(CH$_3$)C(C(O)CH$_3$)C(NPh)S] (**15**) ........ 95
Table S20: Selected bond lengths (Å) and bond angles (°) of **15** ........ 95
Synthesis of [(MeCNH-Dipp)(MeCN-Dipp)C(NH-$^i$PrCN-$^i$Pr)] (**16**) ........ 96
Figure S16: ORTEP diagram of [(MeCNH-Dipp)(MeCN-Dipp)C(NH-$^i$PrCN-$^i$Pr)] (**16**) ........ 97
Table S21: Selected bond lengths (Å) and bond angles (°) of **16** ........ 97
Synthesis of [(MeCN-Dipp)$_2$CH(NH-$^i$PrCN-$^i$Pr)] (**17**) ........ 99
Figure S17: ORTEP diagram of [(MeCN-Dipp)$_2$CH(NH-$^i$PrCN-$^i$Pr)] (**17**) ........ 100
Table S22: Selected bond lengths (Å) and bond angles (°) of **17** ........ 100
Table S23: Selected X-ray crystal structural data and refinement details for compounds **1**-**17** ........ 102
References ........ 106

# General Experimental

All reactions were performed under a protective argon or nitrogen atmosphere using standard glovebox or Schlenk techniques. All solvents, other than deionised water, were dried prior to using by a solvent purification system (Innovative Technologies SPS PS-Micro) to remove any traces of moisture and dissolved oxygen before transfer to a J Young's ampoule and subsequent storage over 4 Å molecular sieves.

$C_6D_6$ was degassed by freeze-pump-thaw methods and stored over activated 4 Å molecular sieves. $CDCl_3$ was stored over activated 4 Å molecular sieves. $^{n}$BuLi (1.6 M in hexane) and $LiCH_2Si(CH_3)_3$ (1.0 M in pentane) were purchased commercially from Sigma-Aldrich, $^{n}$BuLi was used as received, while $LiCH_2Si(CH_3)_3$ was dried in vacuo and stored at -20°C. The parent NacNac(H) ligand and lithium derivative, NacNacLi, were made *via* known literature procedures.[1] Isocyanate, isothiocyanate and carbodiimide reagents were purchased from Sigma-Aldrich and stored over activated 4 Å molecular sieves before use. All other reagents were purchased from commercial sources and were also used as received.

Note that the yields presented herein are crude yields and are the sum of all crystalline material recovered from the respective reactions, including crystalline impurities.

NMR spectra were recorded on a Bruker AV400 MHz spectrometer operating at 400.13 MHz for $^{1}$H, 155.47 MHz for $^{7}$Li and 100.62 MHz for $^{13}$C. All $^{13}$C NMR spectra were proton decoupled. $^{1}$H, $^{13}$C{$^{1}$H} and $^{7}$Li chemical shifts are expressed in parts per million (δ, ppm) and where appropriate referenced to $C_6D_6$ or $CDCl_3$ peaks in the case of $^{1}$H, $^{13}$C{$^{1}$H} spectra.

Melting points were determined in sealed argon filled capillaries and were not corrected.

Infra-Red analysis was carried out on either a PerkinElmer Spectrum 100 Optica FT-IR spectrometer with air and moisture sensitive solid samples prepared in a glovebox using nujol mull. Pro-ligand samples were analysed on a Thermo Scientific Nicolet iS5 FT-IR spectrometer in the solid state. Spectra were normalised and baseline corrected.

Accurate elemental analysis was not possible due to impurities present in the samples of compounds **1-17**, though impurities have been characterised where possible by NMR spectroscopy.

## X-ray crystallography

Data for complexes **1**-**17** were measured with Rigaku Synergy-i or Oxford Diffraction Xcalibur E instruments using monochromated Cu Kα (λ 1.54184 Å) and Mo Kα (λ 0.71073 Å) radiation respectively. Data collection and processing used CrysalisPro software.[2] All structures were refined to convergence on $F^2$ against all independent reflections by the full-matrix least squares method using SHELXL[3] as implemented within OLEX2 or WinGX.[4, 5] For structures **4** and **6** data was measured from twinned samples. Here final refinement was against hklf 5 formatted reflection files. Most structures contained disordered components. Disordered groups were each modelled over two sites with appropriate restraints and constraints applied to bond lengths and displacement ellipsoids to ensure approximation to expected behaviour. Groups modelled in this way were *i*Pr groups in **3**, **7**, **8**, **9**, **10**, **11** and **14**; THF groups in **2a**, **3a**, **7.1**, **7a**, **7b**, **11a** and **13a**; an EtOH solvent and a cyclohexyl ring in **6**; a Et group of **9a**; and the toluene solvent of **7b**. Additionally the full body of structure **12** was modelled as disordered. Selected bond lengths and angles are presented in tables S1-S22, with selected crystallographic data shown in table S23. Deposition Numbers 2298370-2298394 contain the full supplementary crystallographic data for this paper in cif format. These data are provided free of charge by the joint Cambridge Crystallographic Data Centre and Fachinformationszentrum Karlsruhe Access Structures service www.ccdc.cam.ac.uk/structures.

## Synthesis of [(MeCNH-Dipp)(MeCN-Dipp)C($^t$BuNHCO)] (**1**)

NacNacH (2.10 g, 5.0 mmol) dissolved in hexane (10 ml), $^n$BuLi (4.25 ml in hexane, 1.2 M, 5.1 mmol) added at 0°C and stirred overnight to produce a yellow solution. Off-white suspension obtained on addition of $^t$BuNCO (0.6 ml, 5.3 mmol), suspension refluxed (4 hrs) before deionised water (5 ml) was added and mixture exposed to air before being stirred overnight. Separation performed using DCM (15 ml), organic layer dried using $MgSO_4$ before product was filtered, dried, and crystallised from methanol. Product was collected as brown/orange crystals (1.86 g, 3.58 mmol, 72.0 %).

**$^1$H NMR (400.1 MHz, $C_6D_6$, 300 K): δ** 13.42 (s, 1H, NH, NH), 7.15 (broad m, 4H, CH, Ar), 5.29 (s, 1H, NH, C(=O)NH), 3.33 (sep, 4H, CH, $^i$Pr), 2.05 (s, 6H, $CH_3$, Me),1.22 (d, 12H, $CH_3$, $^i$Pr), 1.18 (s, 9H, $CH_3$, $^t$Bu), 1.15 (d, 12H, $CH_3$, $^i$Pr), 0.29 ($H_2O$) ppm

Residual NacNacH: 12.46 (NH), 4.88 (γ-CH), 3.57 (CH, $^i$Pr), 2.14 ($CH_3$, Me) ppm

**$^{13}$C{$^1$H} NMR (100.6 MHz, $C_6D_6$, 300 K): δ** 169.8 ($C_{quaternary}$, C=O), 160.9 ($C_{quaternary}$, CMe), 142.7 ($C_{quaternary}$, Ar), 140.4 ($C_{quaternary}$, Ar), 128.0 ($C_6D_6$), 126.1 (CH, Ar), 123.7 (CH, Ar), 107.8 ($C_{quaternary}$, γ-C), 50.9 ($C_{quaternary}$, $^t$Bu), 28.7 (CH, $^i$Pr), 28.3 ($CH_3$, $^t$Bu), 24.5 ($CH_3$, $^i$Pr), 23.5 ($CH_3$, $^i$Pr), 18.5 ($CH_3$, Me) ppm

**IR spectrum:** ν 3434.6 (s, NH stretching), 1669.6 (s, C=O stretching) $cm^{-1}$

**Melting point analysis**: 157-159 °C

Figure S1: ORTEP diagram of [(MeCNH-Dipp)(MeCN-Dipp)C($^t$BuNHCO)] (**1**)

Molecular structure of **1**, with selected hydrogen atoms displayed and organic groups shown as wire frame for clarity. Thermal ellipsoids are displayed at 40 % probability level.

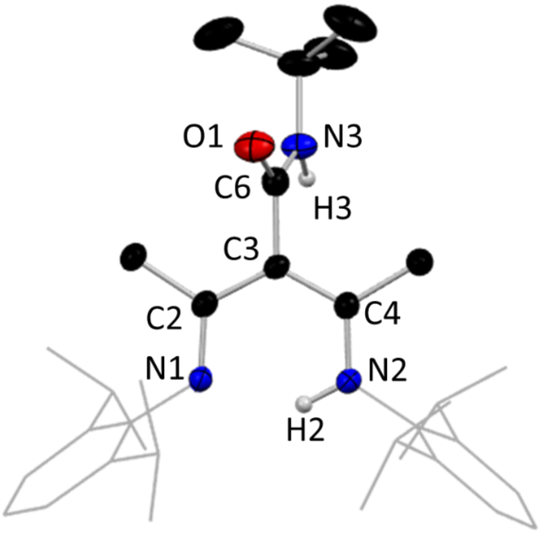


Table S1: Selected bond lengths (Å) and bond angles (°) of **1**

| Atoms | Bond Lengths (Å) |
|---|---|
| N1-C2 | 1.320(2) |
| C2-C3 | 1.423(2) |
| C3-C4 | 1.408(2) |
| C4-N2 | 1.325(2) |
| C3-C6 | 1.499(2) |
| C6-N3 | 1.352(2) |
| C6-O1 | 1.225(2) |
| N3-C7 | 1.478(2) |
| **Atoms** | **Bond Angles (°)** |
| C4-C3-C2 | 123.82(15) |
| C2-C3-C6 | 116.79(14) |
| C4-C3-C6 | 119.34(15) |
| C3-C6-O1 | 122.50(16) |
| O1-C6-N3 | 122.77(17) |
| C3-C6-N3 | 114.74(15) |
| C6-N3-C7 | 126.46(16) |

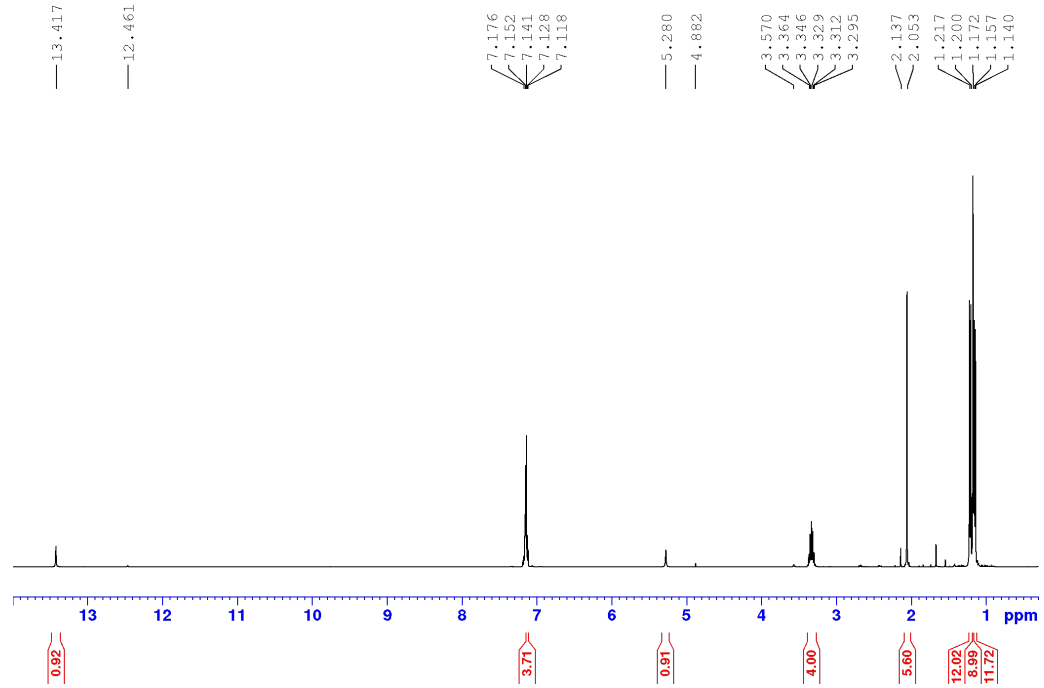


**Figure S1.1**: $^{1}H$ NMR spectrum in $C_6D_6$ of **1** [(MeCNH-Dipp)(MeCN-Dipp)C($^{t}$BuNHCO)]

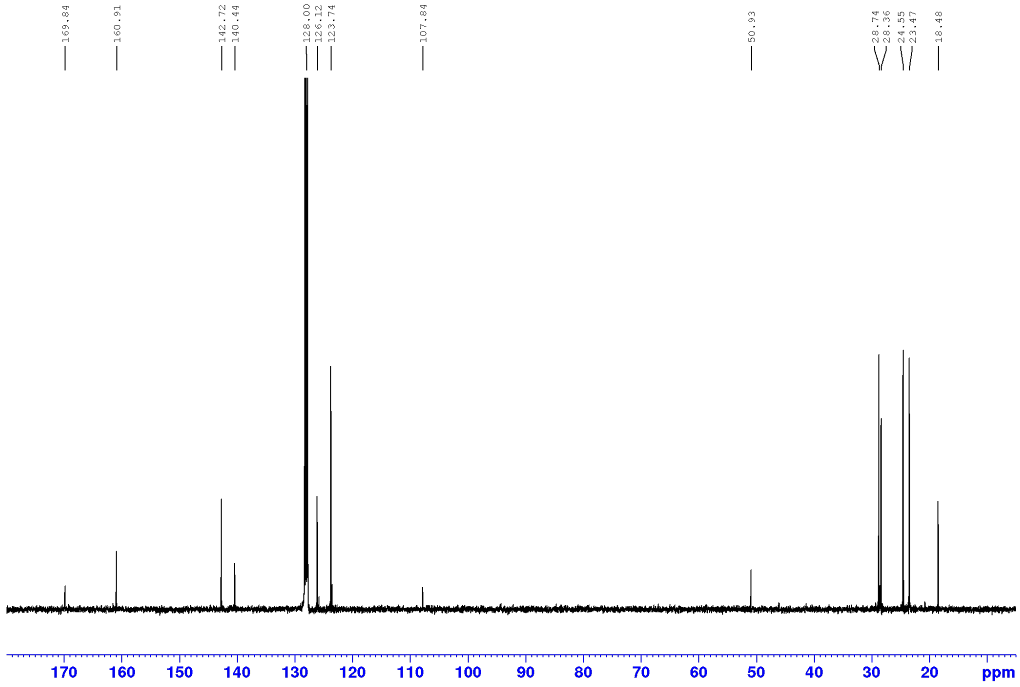


**Figure S1.2**: $^{13}C\{^{1}H\}$ NMR spectrum in $C_6D_6$ of **1** [(MeCNH-Dipp)(MeCN-Dipp)C($^{t}$BuNHCO)]

## Synthesis of [{(MeCN-Dipp)$_2$C($^t$BuNHCO)}Li·2THF] (**1a**)

NacNacLi (0.213 g, 0.5 mmol) placed in vial, dissolved in 5 ml hexane, $^t$BuNCO (57 µl, 0.5 mmol) added to form a peach suspension. THF (2 ml) added to redissolve and crop of pale-yellow crystals grew upon slow evaporation of solvent (0.238 g, 0.36 mmol, 71.2 %).

**$^1$H NMR (400.1 MHz, $C_6D_6$, 300 K): δ** 11.56 (s, 1H, NH, C(=O)NH), 7.25 (br s, 2H, CH, $C_6H_3$), 7.16 ($C_6D_6$), 7.12-7.03 (br m, 2H, CH, $C_6H_3$), 3.45 (br m, 10H, $CH_2$, THF), 3.27 (m, 4H, CH, $^i$Pr), 2.00 (br s, 6H, $CH_3$, Me), 1.54 (br s, 9H, $CH_3$, $^t$Bu), 1.36 (br m, 11H, $CH_2$, THF), 1.29 (br m, 13H, $CH_3$, $^i$Pr), 1.23 (d, 16H, $CH_3$, $^i$Pr), 0.89 (hexane) ppm

Residual NacNacH: 5.68 (γ-CH), 1.96 ($CH_3$, Me), 1.12-1.10 ($CH_3$, $^i$Pr), 1.06 ($CH_3$, $^i$Pr) ppm

**$^{13}$C{$^1$H} NMR (100.6 MHz, $C_6D_6$, 300 K): δ** 169.8 ($C_{quaternary}$, C=O), 148.5 ($C_{quaternary}$, CMe), 140.5 ($C_{quaternary}$, $C_6H_3$), 139.5 ($C_{quaternary}$, $C_6H_3$), 138.4 ($C_{quaternary}$, $C_6H_3$), 128.0 ($C_6D_6$), 123.5 (CH, $C_6H_3$), 122.8 (CH, $C_6H_3$), 100.5 ($C_{quaternary}$, γ-C), 68.0 ($CH_2$, THF), 49.7 ($C_{quaternary}$, $^t$Bu), 30.1 ($CH_3$, $^t$Bu), 28.3 (CH, $^i$Pr), 28.2 (CH, $^i$Pr), 28.0 (CH, $^i$Pr), 25.6 ($CH_2$, THF), 25.1 ($CH_3$, $^i$Pr), 24.5 ($CH_3$, $^i$Pr), 23.7 ($CH_3$, $^i$Pr), 23.5 ($CH_3$, $^i$Pr) ppm

**$^7$Li NMR (155.5 MHz, $C_6D_6$, 298K) δ** 1.75 (NacNacLi), 1.33 (**1a**) ppm

**IR spectrum:** ν 1612.7 (s, C=O stretching) $cm^{-1}$

**Melting point analysis**: 120-122 °C

Figure S1a: ORTEP diagram of [{(MeCN-Dipp)$_2$C($^t$BuNHCO)}Li·2THF] (**1a**)

Molecular structure of **1a**, with selected hydrogen atom displayed and organic groups shown as wire frame for clarity. Thermal ellipsoids are displayed at 40 % probability level, second molecule not shown.

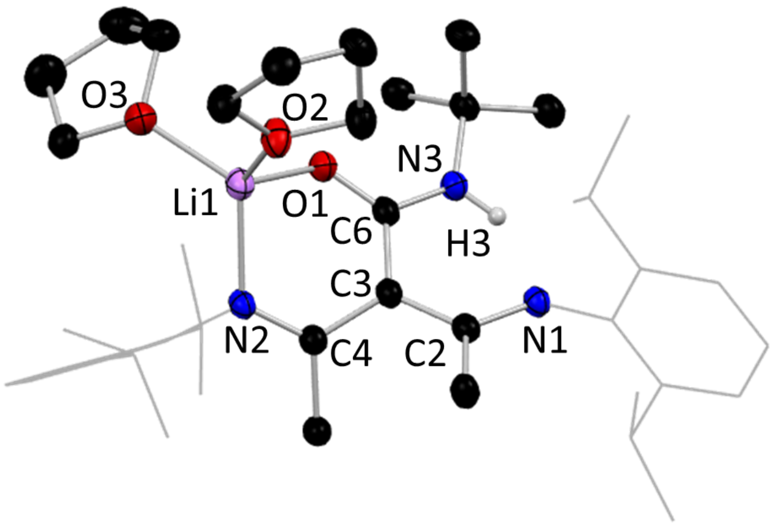

Table S2: Selected bond lengths (Å) and bond angles (°) of **1a**

| **Atoms** | **Bond Lengths (Å)** |
|---|---|
| Li1-O1 | 1.850(2) |
| Li1-O2 | 1.971(2) |
| Li1-O3 | 1.959(2) |
| Li1-N2 | 1.996(2) |
| N1-C2 | 1.3087(15) |
| C2-C3 | 1.4396(17) |
| C3-C4 | 1.4530(16) |
| C4-N2 | 1.3055(16) |
| C3-C6 | 1.4762(16) |
| C6-N3 | 1.3514(15) |
| C6-O1 | 1.2590(15) |
| N3-C7 | 1.4705(15) |
| **Atoms** | **Bond Angles (°)** |
| N2-Li1-O1 | 90.36(10) |
| N2-Li1-O2 | 122.89(12) |
| N2-Li1-O3 | 121.09(12) |
| O1-Li1-O2 | 107.73(11) |
| O1-Li1-O3 | 114.37(12) |
| O2-Li1-O3 | 100.15(10) |
| C4-C3-C2 | 121.45(11) |
| C2-C3-C6 | 121.20(10) |
| C4-C3-C6 | 117.12(10) |
| C3-C6-O1 | 123.31(10) |
| O1-C6-N3 | 120.01(11) |
| C3-C6-N3 | 116.65(10) |
| C6-N3-C7 | 125.71(10) |

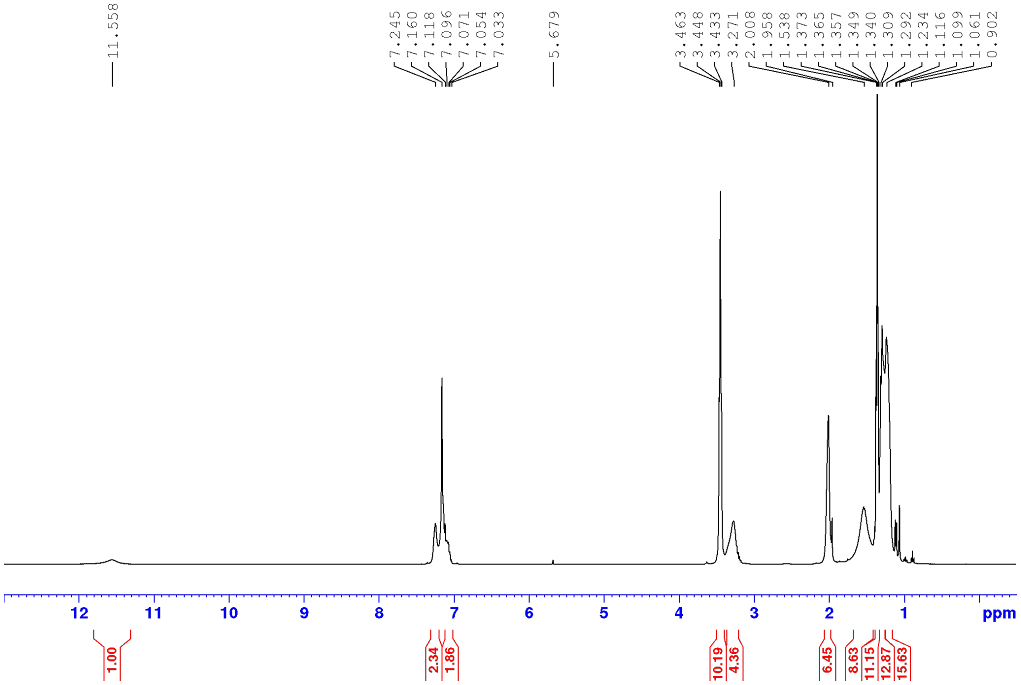


**Figure S1a.1**: $^{1}H$ NMR spectrum in $C_6D_6$ of **1a** [{(MeCN-Dipp)$_2$C($^{t}$BuNHCO)}Li·2THF]

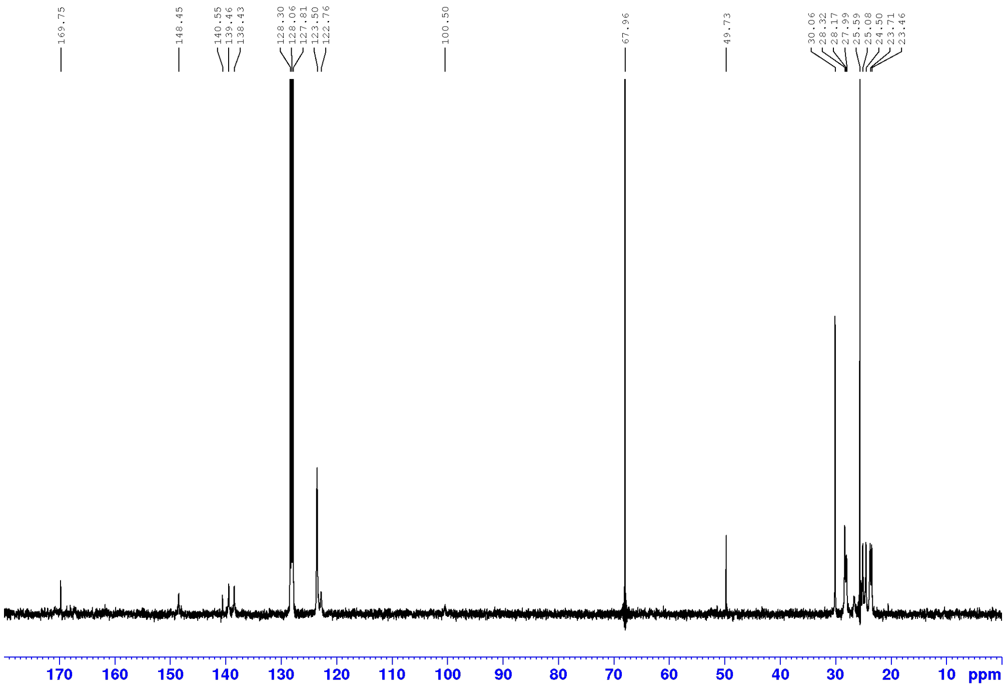


**Figure S1a.2**: $^{13}C\{^{1}H\}$ NMR spectrum in $C_6D_6$ of **1a** [{(MeCN-Dipp)$_2$C($^{t}$BuNHCO)}Li·2THF]

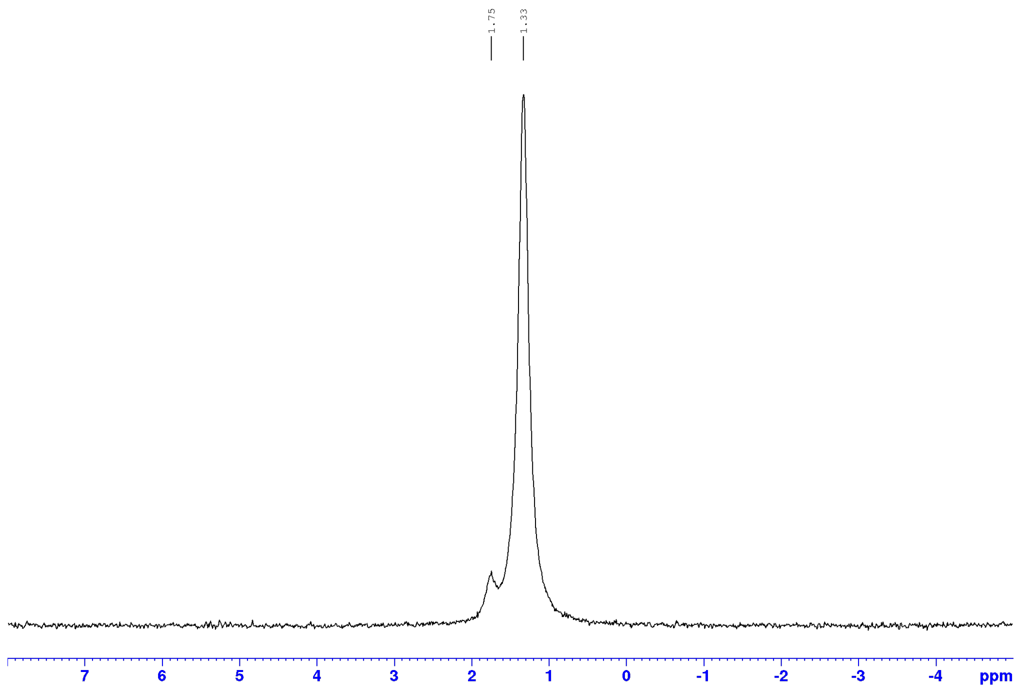


**Figure S1a.3**: $^{7}Li$ NMR spectrum in $C_6D_6$ of **1a** [{(MeCN-Dipp)$_2$C($^t$BuNHCO)}Li·2THF]

## Synthesis of [(MeCNH-Dipp)(MeCN-Dipp)C(4-OMe$C_6H_4$NHCO)] (**2**)

NacNacLi (0.860 g, 2.0 mmol) dissolved in hexane (10 ml) to produce a yellow solution before 4-OMePhNCO (0.26 ml, 2.0 mmol) was added giving a white suspension. Suspension stirred (3 hrs) before deionised water (5 ml) was added, mixture exposed to air and stirred overnight. Separation performed using DCM (15 ml), organic layer dried using $MgSO_4$ before product was filtered, dried, and crystallised from methanol. Product was collected as pale-yellow crystals (0.750 g, 1.18 mmol, 58.9 %).

**$^1$H NMR (400.1 MHz, $CDCl_3$, 300 K): δ** 13.29 (s, 1H, NH, NH), 11.45 (unknown impurity), 7.52 (m, 2H, CH, $C_6H_4$), 7.26 ($CDCl_3$), 7.14 (broad m, 8H, CH, $C_6H_3$), 7.06 (m, 2H, CH, $C_6H_3$), 6.91 (m, 2H, CH, $C_6H_4$), 6.85 (m, 2H, CH, $C_6H_4$), 3.81 (DCM), 3.07 (m, 4H, CH, $^i$Pr), 1.91 (s, 6H, $CH_3$, Me), 1.80 (s, 3H, $CH_3$, OMe), 1.57 ($H_2O$), 1.22 (d, 13H, $CH_3$, $^i$Pr), 1.13 (d, 14H, $CH_3$, $^i$Pr) ppm

Residual NacNacH: 12.19 (NH), 7.44 (CH, $C_6H_3$), 3.16 (CH, $^i$Pr), 2.91 (CH, $^i$Pr), 2.72 (CH, $^i$Pr), 1.94 ($CH_3$, Me), 1.26-1.12 ($CH_3$, $^i$Pr), 1.09-1.03 ($CH_3$, $^i$Pr) ppm

**$^{13}$C{$^1$H} NMR (100.6 MHz, $CDCl_3$, 300 K): δ** 161.0 ($C_{quaternary}$, C=O), 142.3 ($C_{quaternary}$, CMe), 125.6 (CH, $C_6H_3$), 123.3 (CH, $C_6H_3$), 121.6 (CH, $C_6H_4$), 114.3 (CH, $C_6H_4$), 77.0 ($CDCl_3$), 55.6 (DCM), 28.3 (CH, $^i$Pr), 24.3 ($CH_3$, $^i$Pr), 23.4 ($CH_3$, $^i$Pr), 18.8 ($CH_3$, OMe), 18.6 ($CH_3$, Me) ppm

**IR spectrum:** $\nu$ 3198.8 (s, NH stretching), 3086.0 (broad s, NH stretching) 1650.8 (s, C=O stretching) $cm^{-1}$

**Melting point analysis**: 175-177 °C

X-ray structural data obtained for compound **2** were of insufficient quality for publication.

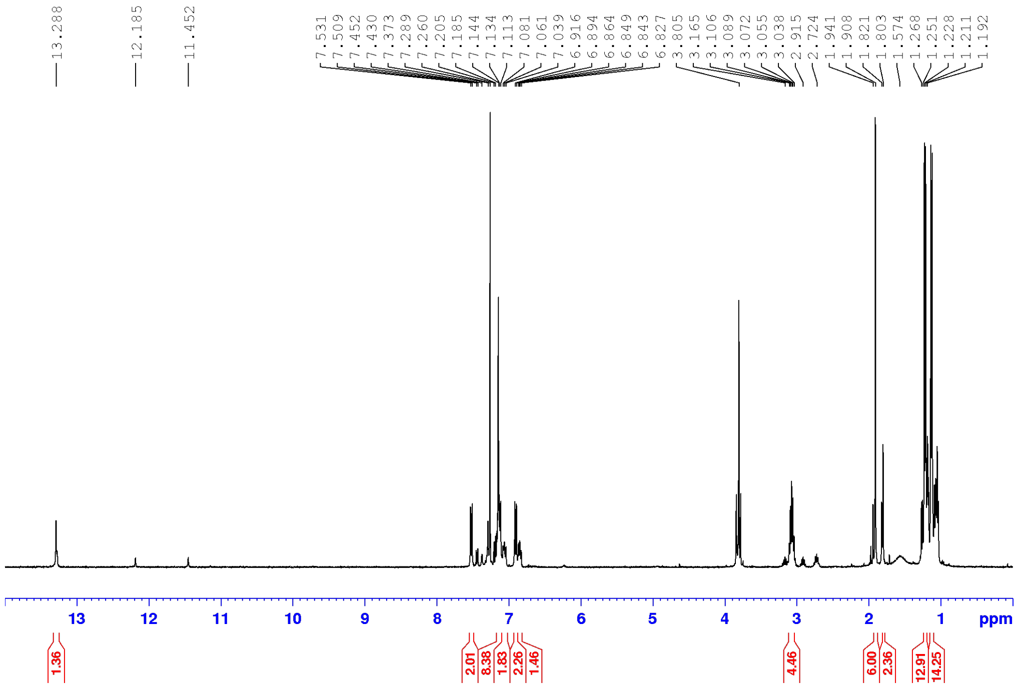


**Figure S2.1**: $^{1}H$ NMR spectrum in $C_6D_6$ of **2** [(MeCNH-Dipp)(MeCN-Dipp)C(4-OMe$C_6H_4$NHCO)]

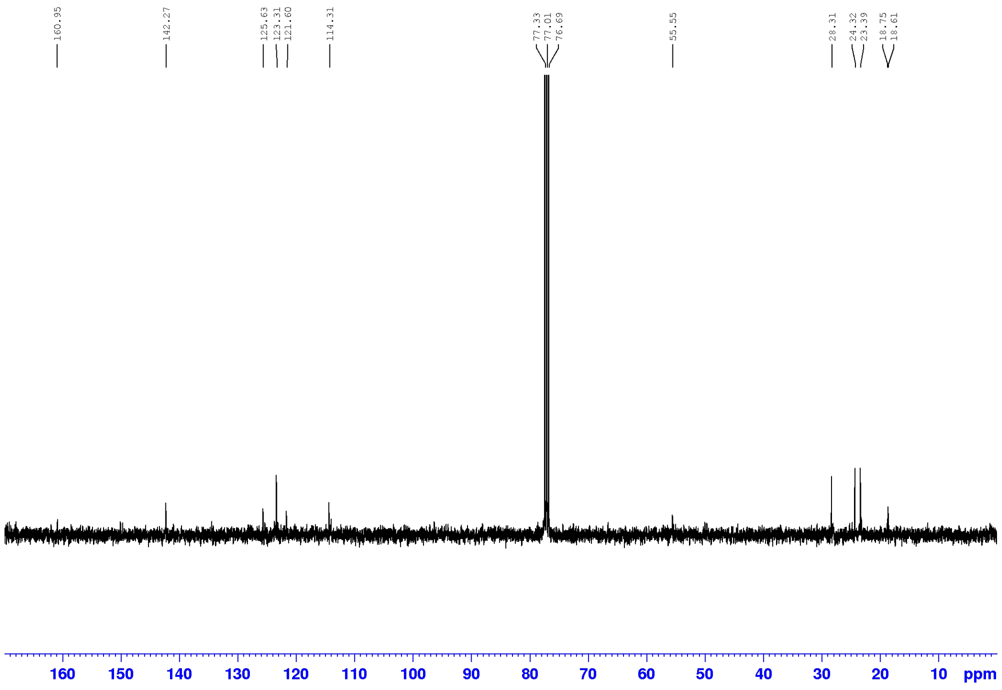


**Figure S2.2**: $^{13}C\{^{1}H\}$ NMR spectrum in $C_6D_6$ of **2** [(MeCNH-Dipp)(MeCN-Dipp)C(4-OMe$C_6H_4$NHCO)]

## Synthesis of [{(MeCN-Dipp)$_2$C(4-OMe$C_6H_4$NHCO)}Li·2THF] (**2a**)

NacNacLi (0.043 g, 0.1 mmol) placed in vial, dissolved in 1 ml hexane, 4-OMePhNCO (13 µl, 0.1 mmol) added to form a yellow suspension. THF (0.5 ml) added to regain solution and reaction placed in -20 °C freezer. Crop of colourless crystals grew overnight (0.029 g, 0.04 mmol, 40.3 %).

**$^{1}$H NMR (400.1 MHz, $C_6D_6$, 300 K): δ** 13.42 (s, 1H, NH, C(=O)NH), 7.16 ($C_6D_6$), 7.10 (m, 6H, CH, $C_6H_3$), 6.83 (m, 3H, CH, $C_6H_4$), 3.64 (br s, 9H, $CH_2$, THF), 3.20 (m, 2H, CH, $^{i}$Pr), 2.83 (m, 3H, CH, $^{i}$Pr), 2.21 (s, 3H, $CH_3$, OMe), 2.09 (s, 6H, $CH_3$, Me), 1.49 (br s, 9H, $CH_2$, THF), 1.15 (d, 7H, $CH_3$, $^{i}$Pr), 1.09 (d, 7H, $CH_3$, $^{i}$Pr), 1.02 (d, 12H, $CH_3$, $^{i}$Pr), 0.89 (hexane) ppm

Residual NacNacH: 12.47 (NH), 4.89 (γ-CH), 3.31 (CH, $^{i}$Pr), 2.02 ($CH_3$, Me), 1.23-1.17 ($CH_3$, $^{i}$Pr) ppm

**$^{13}$C{$^{1}$H} NMR (100.6 MHz, $C_6D_6$, 300 K): δ** 176.8 ($C_{quaternary}$, C=O), 160.6 ($C_{quaternary}$, CMe), 148.1 ($C_{quaternary}$, Ar), 142.9 ($C_{quaternary}$, $^{i}$Pr), 142.2 ($C_{quaternary}$, $^{i}$Pr), 140.6 ($C_{quaternary}$, Ar), 130.1 (CH, Ar), 129.0 (CH, Ar), 128.0 ($C_6D_6$), 125.8 (CH, Ph), 123.8 (CH, Ph), 123.6 (CH, Ph), 123.4 (CH, Ph), 106.6 ($C_{quaternary}$, γ-C), 67.9 (THF), 28.4 (CH, $^{i}$Pr), 28.0 (CH, $^{i}$Pr), 25.8 (THF), 24.7 ($CH_3$, $^{i}$Pr), 24.6 ($CH_3$, $^{i}$Pr), 23.9 ($CH_3$, $^{i}$Pr), 23.3 ($CH_3$, $^{i}$Pr), 20.9 (CH, CH-OMe), 19.8 ($CH_3$, Me) ppm

**$^{7}$Li NMR (155.5 MHz, $C_6D_6$, 298K) δ** 1.74 ppm

**IR spectrum:** ν 1631.0 (s, C=O stretching) $cm^{-1}$

**Melting point analysis**: 123-125 °C

Figure S2a: ORTEP diagram of [{(MeCN-Dipp)2C(4-OMeC6H4NHCO)}Li·2THF] (**2a**)

Molecular structure of **2a**, with selected hydrogen atom displayed and organic groups shown as wire frame for clarity. Thermal ellipsoids are displayed at 40 % probability level.

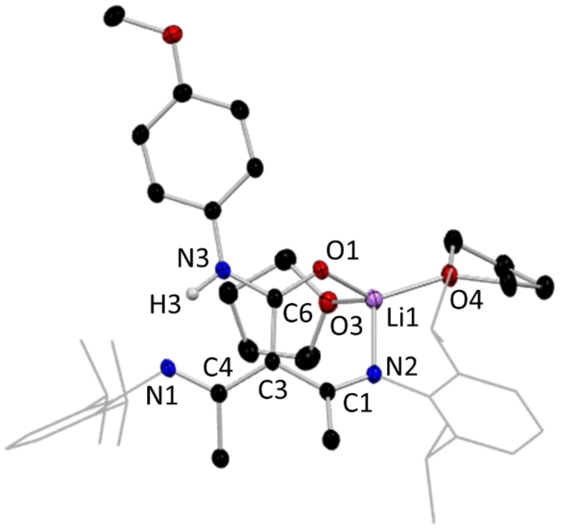

Table S3: Selected bond lengths (Å) and bond angles (°) of **2a**

| Atoms | Bond Lengths (Å) |
|---|---|
| Li1-N1 | 2.015(2) |
| Li1-O1 | 1.8797(19) |
| Li1-O3 | 1.964(2) |
| Li1-O4 | 1.9686(19) |
| N2-C4 | 1.3139(13) |
| C4-C3 | 1.4445(13) |
| C3-C1 | 1.4667(13) |
| C1-N1 | 1.3001(13) |
| C3-C6 | 1.4627(14) |
| C6-O1 | 1.2582(12) |
| C6-N3 | 1.3671(13) |
| N3-C7 | 1.4050(13) |
| C10-O2 | 1.3729(13) |
| O2-C13 | 1.4211(13) |
| **Atoms** | **Bond Angles (°)** |
| N1-Li1-O1 | 88.66(8) |
| N1-Li1-O3 | 113.92(9) |
| N1-Li1-O4 | 115.16(9) |
| O1-Li1-O3 | 102.50(9) |
| O1-Li1-O4 | 135.62(10) |
| O3-Li1-O4 | 100.78(9) |
| C1-C3-C4 | 120.17(9) |
| C4-C3-C6 | 122.95(9) |
| C1-C3-C6 | 116.74(8) |
| O1-C6-C3 | 124.17(9) |
| C3-C6-N3 | 116.23(8) |
| N3-C6-O1 | 119.60(9) |

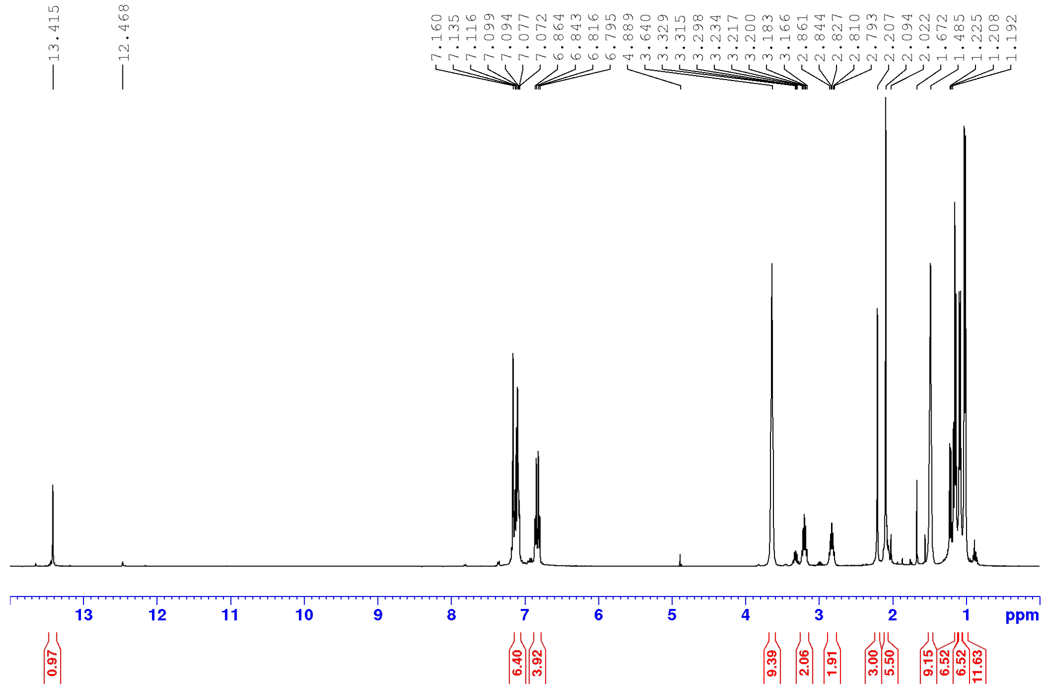


**Figure S2a.1**: $^{1}H$ NMR spectrum in $C_6D_6$ of **2a** [{(MeCN-Dipp)$_2$C(4-OMeC$_6$H$_4$NHCO)}Li·2THF]

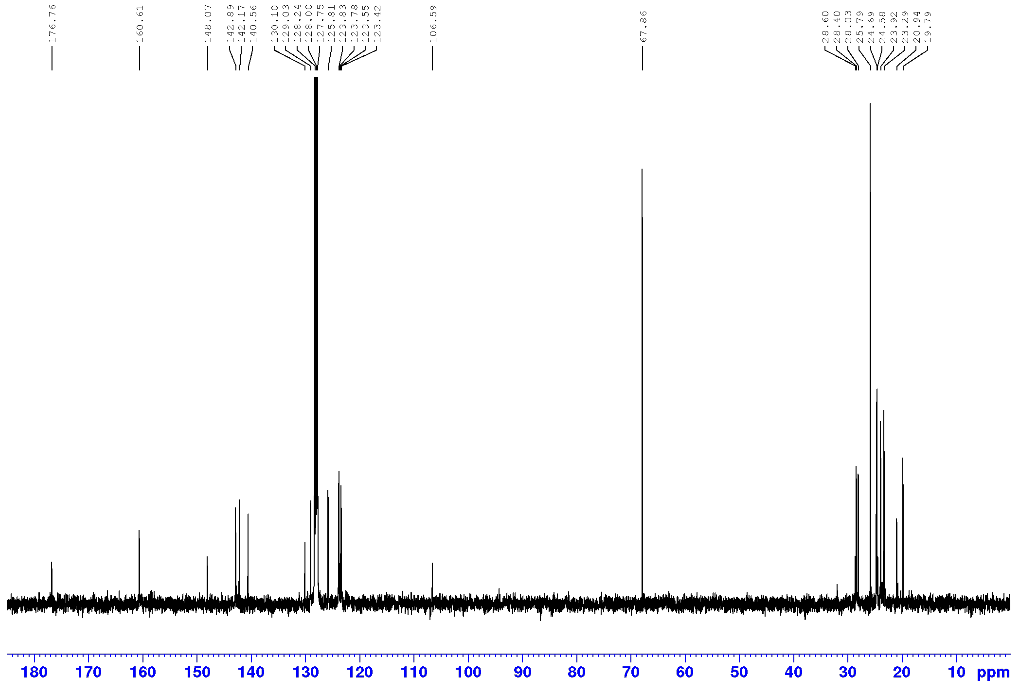


**Figure S2a.2**: $^{13}C\{^{1}H\}$ NMR spectrum in $C_6D_6$ of **2a** [{(MeCN-Dipp)$_2$C(4-OMeC$_6$H$_4$NHCO)}Li·2THF]

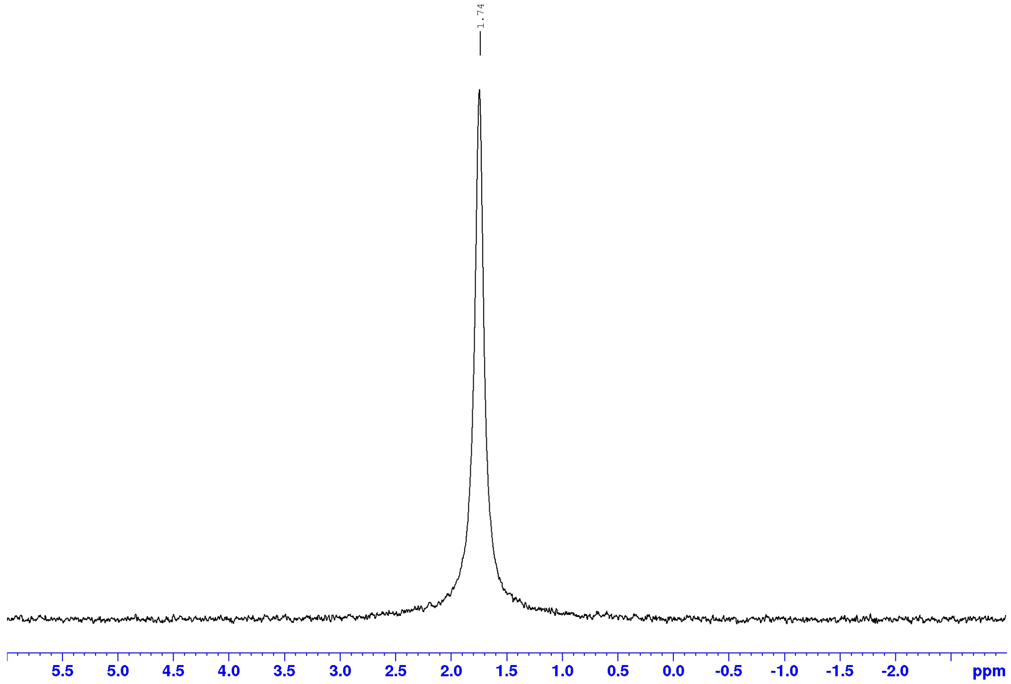


**Figure S2a.3**: $^{7}$Li NMR spectrum in $C_6D_6$ of **2a** [{(MeCN-Dipp)$_2$C(4-OMeC$_6$H$_4$NHCO)}Li·2THF]

## Synthesis of [(MeCNH-Dipp)(MeCN-Dipp)C(4-Me$C_6H_4$NHCO)] (**3**)

NacNacH (2.10 g, 5.0 mmol) dissolved in hexane (10 ml), $^{n}$BuLi (4.25 ml, 1.2 M, 5.1 mmol) added at 0°C and stirred overnight to produce a yellow solution. Yellow suspension obtained on addition of 4-MePhNCO (0.65 ml, 5.2 mmol), suspension refluxed (4 hrs) before deionised water (5 ml) was added and mixture exposed to air before being stirred overnight. Separation performed using DCM (15 ml), organic layer dried using $MgSO_4$ before product was filtered, dried, and crystallised from methanol. Product was collected as yellow crystals (1.87 g, 3.38 mmol, 68.0 %).

**$^{1}$H NMR (400.1 MHz, $CDCl_3$, 300 K): δ** 13.32 (br s, 1H, NH, NH), 11.57 (unknown impurity), 7.51 (m, 2H, CH, $C_6H_3$), 7.34 (m, 2H, CH, $C_6H_3$), 7.26 ($CDCl_3$), 7.22-7.09 (br m, 14H, CH, $C_6H_3$/$C_6H_4$), 3.09 (sep, 5H, CH, $^{i}$Pr), 2.34 (s, 3H, $CH_3$, $C_6H_4$Me), 1.92 (s, 6H, $CH_3$, Me), 1.81 (s, 2H, NH, C(=O)NH), 1.62 ($H_2O$), 1.23 (d, 12H, $CH_3$, $^{i}$Pr), 1.20 (hexane), 1.15 (d, 14H, $CH_3$, $^{i}$Pr), 0.90 (hexane) ppm

Residual NacNacH: 12.21 (NH), 4.66 (γ-CH), 3.18 (CH, $^{i}$Pr), 2.92 (CH, $^{i}$Pr), 2.77 (CH, $^{i}$Pr), 1.99 ($CH_3$, Me), 1.27-1.21 ($CH_3$, $^{i}$Pr), 1.11-1.03 ($CH_3$, $^{i}$Pr) ppm

**$^{13}$C{$^{1}$H} NMR (100.6 MHz, $CDCl_3$, 300 K): δ** 169.7 ($C_{quaternary}$, C=O), 167.9 ($C_{quaternary}$, CMe), 161.1 ($C_{quaternary}$, COMe), 160.7 (unknown impurity), 146.7 ($C_{quaternary}$, $C_6H_4$Me), 142.4 ($C_{quaternary}$, $C_6H_3$), 139.7 ($C_{quaternary}$, $C_6H_3$), 136.9 ($C_{quaternary}$, $C_6H_3$), 134.2 (unknown impurity), 129.7 (CH, $C_6H_3$/ $C_6H_4$), 128.3 (unknown impurity), 125.8 (CH, $C_6H_3$/ $C_6H_4$), 123.4 (CH, $C_6H_3$/ $C_6H_4$), 120.9 (CH, $C_6H_3$), 120.0 (CH, $C_6H_3$), 105.7 ($C_{quaternary}$, γ-C), 77.2 ($CDCl_3$), 28.4 (CH, $^{i}$Pr), 24.9 (unknown impurity), 24.5 ($CH_3$, $^{i}$Pr), 23.5 ($CH_3$, $^{i}$Pr), 22.9 (hexane), 21.0 ($CH_3$, - $C_6H_4$Me), 18.8 ($CH_3$, Me) ppm

Residual NacNacH: 162.5 ($C_{quaternary}$, CMe), 129.3 (CH, $C_6H_3$), 123.8 (CH, $C_6H_3$), 123.6 (CH, $C_6H_3$), 28.7 (CH, $^{i}$Pr), 28.3 (CH, $^{i}$Pr), 28.2 (CH, $^{i}$Pr), 26.1 ($CH_3$, Me), 23.8 ($CH_3$, $^{i}$Pr), 23.3 ($CH_3$, $^{i}$Pr) ppm

**IR spectrum:** ν 3195.0 (s, NH stretching), 3092.3 (broad s, NH stretching), 1648.4 (s, C=O stretching) $cm^{-1}$

**Melting point analysis**: 163-165 °C

Figure S3: ORTEP diagram of [(MeCNH-Dipp)(MeCN-Dipp)C(4-$MeC_6H_4NHCO$)] (**3**)

Molecular structure of **3**, with selected hydrogen atoms displayed and organic groups shown as wire frame for clarity. Thermal ellipsoids are displayed at 40 % probability level.

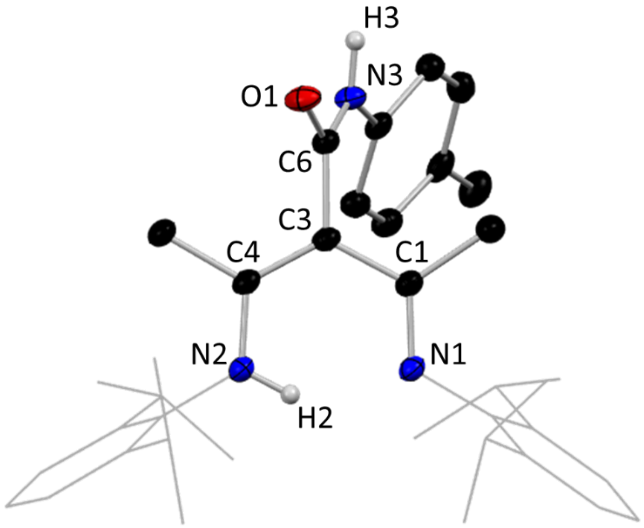


Table S4: Selected bond lengths (Å) and bond angles (°) of **3**

| Atoms | Bond Lengths (Å) |
|---|---|
| N1-C1 | 1.306(2) |
| C1-C3 | 1.449(2) |
| C3-C4 | 1.394(2) |
| C4-N2 | 1.343(2) |
| C3-C6 | 1.498(2) |
| C6-N3 | 1.359(2) |
| C6-O1 | 1.2418(19) |
| **Atoms** | **Bond Angles (°)** |
| C1-C3-C6 | 118.48(13) |
| C4-C3-C6 | 117.91(13) |
| O1-C6-N3 | 118.16(14) |

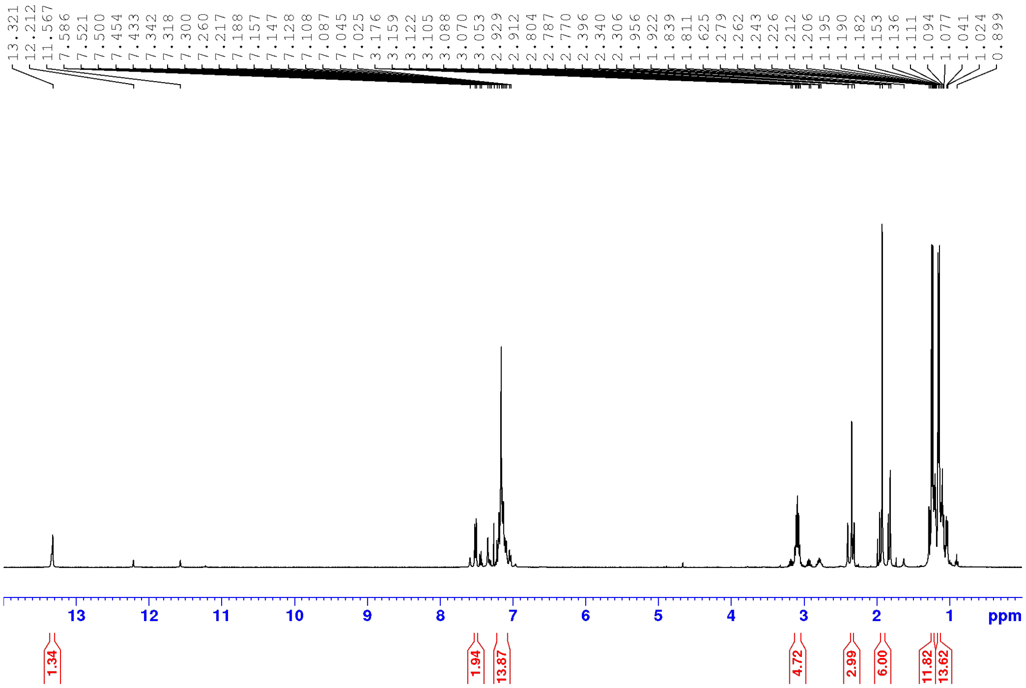


**Figure S3.1**: $^{1}H$ NMR spectrum in $C_6D_6$ of **3** [(MeCNH-Dipp)(MeCN-Dipp)C(4-$MeC_6H_4$NHCO)]

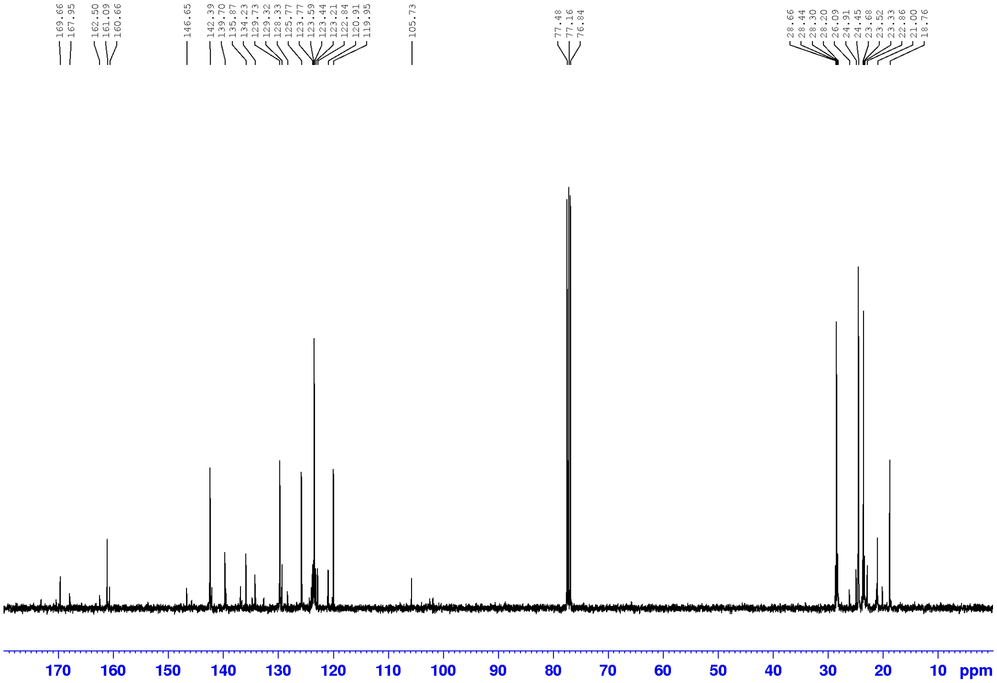


**Figure S3.2**: $^{13}C\{^{1}H\}$ NMR spectrum in $C_6D_6$ of **3** [(MeCNH-Dipp)(MeCN-Dipp)C(4-$MeC_6H_4$NHCO)]

## Synthesis of [{(MeCN-Dipp)$_2$C(4-MeC$_6$H$_4$NHCO)}Li·2THF](**3a**)

NacNacLi (0.043 g, 0.1 mmol) placed in vial, dissolved in hexane (1 ml), p-tolylNCO (12 µl, 0.1 mmol) added to form a yellow suspension. THF (0.5 ml) added to regain solution and mixture placed in -20 °C freezer. A crop of colourless crystals grew overnight (0.025 g, 0.04 mmol, 44.7 %).

**$^1$H NMR (400.1 MHz, $C_6D_6$, 300 K): δ** 13.42 (s, 1H, NH, C(=O)NH), 7.16 ($C_6D_6$), 7.10 (broad m, 6H, CH, $C_6H_3$), 6.83 (m, 4H, CH, $C_6H_4$), 3.64 (s, 9H, $CH_2$, THF), 3.20 (m, 2H, CH, $^i$Pr), 2.83 (m, 2H, CH, $^i$Pr), 2.21 (s, 3H, $CH_3$, $C_6H_4$Me), 2.10 (s, 5H, $CH_3$, Me), 1.49 (s, 9H, $CH_2$, THF), 1.15 (d, 6H, $CH_3$, $^i$Pr), 1.09 (d, 6H, $CH_3$, $^i$Pr), 1.02 (d, 11H, $CH_3$, $^i$Pr), 0.89 (m, hexane) ppm

Residual NacNacH: 12.47 (NH), 4.89 (γ-CH), 3.31 (CH, $^i$Pr), 2.03 ($CH_3$, Me), 1.22-1.17 ($CH_3$, $^i$Pr) ppm

**$^{13}$C{$^1$H} NMR (100.6 MHz, $C_6D_6$, 300 K): δ** 176.8 ($C_{quaternary}$, C=O), 160.6 ($C_{quaternary}$, CMe), 148.1 ($C_{quaternary}$, $C_6H_3$), 142.9 ($C_{quaternary}$, $C_6H_3$), 142.2 ($C_{quaternary}$, $C_6H_4$), 140.6 ($C_{quaternary}$, $C_6H_4$), 130.1 (CH, $C_6H_4$), 129.0 (CH, $C_6H_4$), 128.2-127.8 ($C_6D_6$), 125.8 (CH, $C_6H_3$), 123.8 (CH, $C_6H_3$), 123.8 (CH, $C_6H_3$), 123.6 (CH, $C_6H_3$), 123.4 (CH, $C_6H_3$), 106.6 ($C_{quaternary}$, γ-C), 67.9 ($CH_2$, THF), 28.4 (CH, $^i$Pr), 28.0 (CH, $^i$Pr), 25.8 ($CH_2$, THF), 24.7 ($CH_3$, $^i$Pr), 24.6 ($CH_3$, $^i$Pr), 23.9 ($CH_3$, $^i$Pr), 23.3 ($CH_3$, $^i$Pr), 20.9 ($CH_3$, Me), 19.8 ($CH_3$, Me) ppm

**$^7$Li NMR (155.5 MHz, $C_6D_6$, 298K): δ** 1.74 ppm

**IR spectrum:** ν 1609.3 (s, C=O stretching) $cm^{-1}$

**Melting point analysis**: 185-187 °C

Figure S3a: ORTEP diagram of [{(MeCN-Dipp)$_2$C(4-MeC$_6$H$_4$NHCO)}Li·2THF](**3a**)

Molecular structure of **3a**, with selected hydrogen atom displayed and organic groups shown as wire frame for clarity. Thermal ellipsoids are displayed at 40 % probability level.

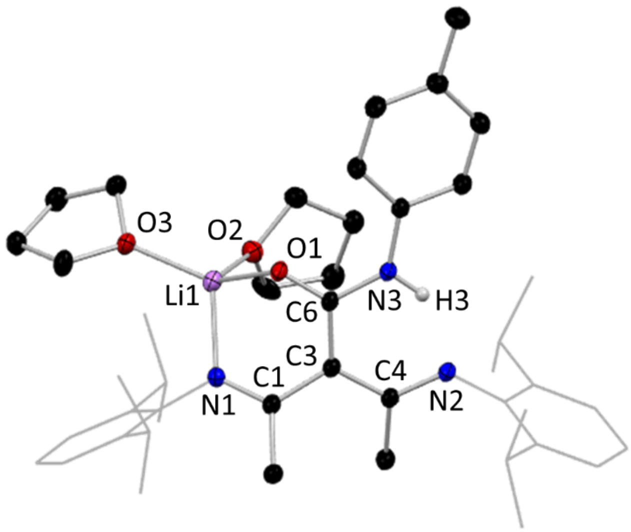

Table S5: Selected bond lengths (Å) and bond angles (°) of **3a**

| **Atoms** | **Bond Lengths (Å)** |
|---|---|
| Li1-O1 | 1.879(2) |
| Li1-O2 | 1.960(2) |
| Li1-O3 | 1.963(2) |
| Li1-N1 | 2.015(2) |
| N1-C1 | 1.3010(15) |
| C1-C3 | 1.4638(16) |
| C3-C4 | 1.4455(16) |
| C4-N2 | 1.3118(15) |
| C3-C6 | 1.4635(16) |
| C6-N3 | 1.3677(15) |
| C6-O1 | 1.2571(14) |
| N3-C7 | 1.4035(15) |
| **Atoms** | **Bond Angles (°)** |
| N1-Li1-O1 | 89.08(9) |
| N1-Li1-O2 | 114.41(11) |
| N1-Li1-O3 | 116.32(11) |
| O1-Li1-O2 | 102.60(10) |
| O1-Li1-O3 | 133.64(12) |
| O2-Li1-O3 | 100.73(10) |
| C4-C3-C1 | 119.97(10) |
| C1-C3-C6 | 117.29(10) |
| C4-C3-C6 | 122.68(10) |
| C3-C6-O1 | 124.16(11) |
| O1-C6-N3 | 119.53(10) |
| C3-C6-N3 | 116.30(10) |
| C6-N3-C7 | 128.35(10) |

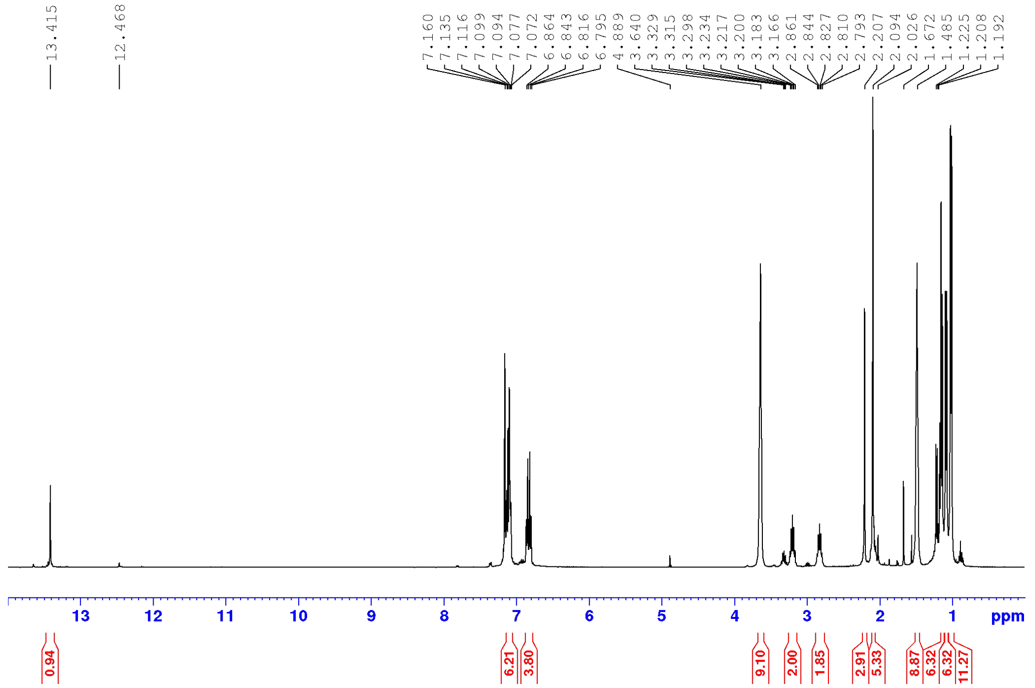


**Figure S3a.1**: $^{1}H$ NMR spectrum in $C_6D_6$ of **3a** [{(MeCN-Dipp)$_2$C(4-MeC$_6$H$_4$NHCO)}Li·2THF]

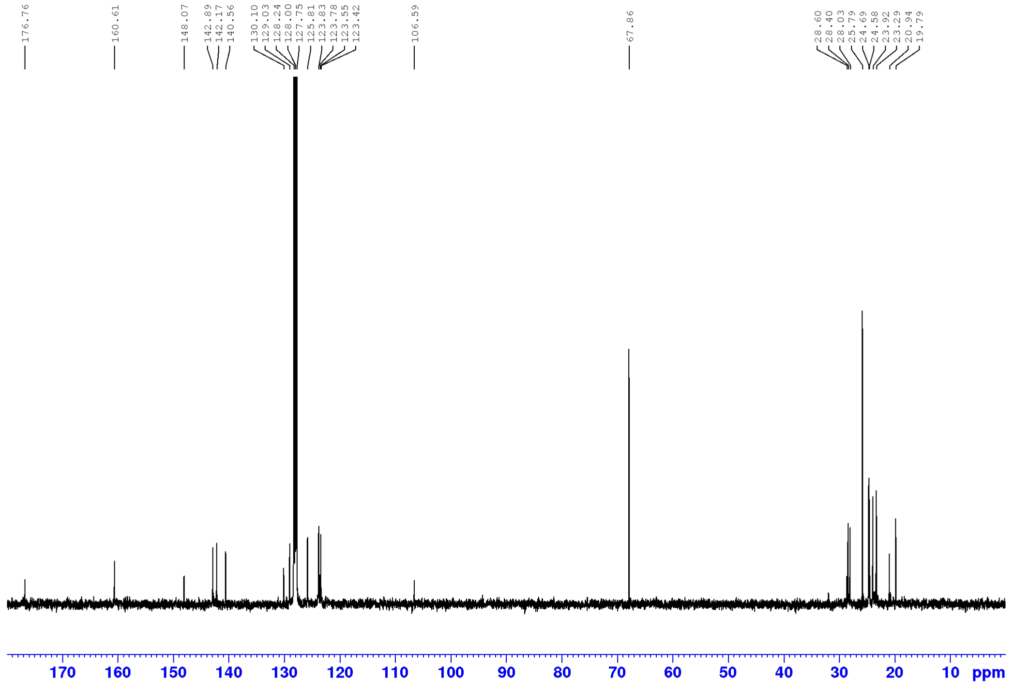


**Figure S3a.2**: $^{13}C\{^{1}H\}$ NMR spectrum in $C_6D_6$ of **3a** [{(MeCN-Dipp)$_2$C(4-MeC$_6$H$_4$NHCO)}Li·2THF]

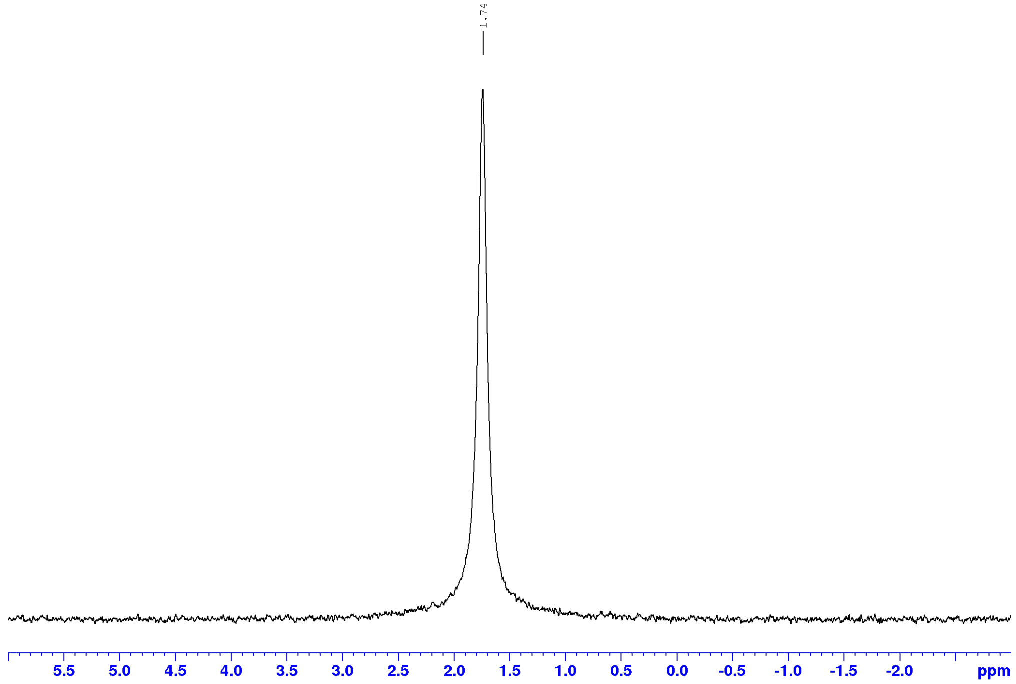


**Figure S3a.3**: $^{7}$Li NMR spectrum in $C_6D_6$ of **3a** [{(MeCN-Dipp)$_2$C(4-MeC$_6$H$_4$NHCO)}Li·2THF]

## Synthesis of [(MeCNH-Dipp)(MeCN-Dipp)C(2-$MeC_6H_4$NHCO)] (**4**)

NacNacLi (0.86 g, 2.0 mmol) dissolved in hexane (10 ml) to produce a yellow solution before 2-MePhNCO (0.25 ml, 2.0 mmol) was added giving a yellow suspension. Suspension stirred (3 hrs) before deionised water (5 ml) was added and mixture exposed to air and stirred overnight. Separation performed using DCM (15 ml), organic layer dried using $MgSO_4$ before product was filtered, dried, and crystallised from methanol. Product was collected as off-white/yellow crystals (0.99 g, 1.86 mmol, 93.0 %). Improved crystallographic data were obtained on samples recrystallised from acetonitrile.

**$^1$H NMR (400.1 MHz, $CDCl_3$, 300 K): δ** 13.31 (s, 1H, NH, NH), 11.70 (unknown impurity), 8.04 (m, 1H, CH, $C_6H_3$), 7.26 ($CDCl_3$), 7.22-7.16 (broad m, 12H, CH, $C_6H_3$/ $C_6H_4$), 3.10 (broad m, 4H, CH, $^i$Pr), 2.31 (s, 3H, $CH_3$, $C_6H_4$Me), 1.96 (s, 6H, $CH_3$, Me), 1.24 (d, 12H, $CH_3$, $^i$Pr), 1.15 (d, 12H, $CH_3$, $^i$Pr) ppm

Residual NacNacH: 12.25 (NH), 7.82 (CH, Ph), 4.70 (γ-CH), 2.90 (CH, $^i$Pr), 2.75 (CH, $^i$Pr), 1.78 ($CH_3$, Me) ppm

**$^{13}$C{$^1$H} NMR (100.6 MHz, $CDCl_3$, 300 K): δ** 169.6 ($C_{quaternary}$, C=O), 160.9 ($C_{quaternary}$, CMe), 142.2 ($C_{quaternary}$, $C_6H_3$), 139.6 ($C_{quaternary}$, $C_6H_3$), 130.6 (CH, $C_6H_3$/$C_6H_4$), 127.1 (CH, $C_6H_3$/$C_6H_4$), 125.7 (CH, $C_6H_3$/$C_6H_4$), 125.0 (CH, $C_6H_3$/$C_6H_4$), 123.6 (CH, $C_6H_3$/$C_6H_4$), 123.4 (CH, $C_6H_3$/$C_6H_4$), 122.2 (CH, $C_6H_3$/$C_6H_4$), 77.1 ($CDCl_3$), 28.3 (CH, $^i$Pr), 24.3 ($CH_3$, $^i$Pr), 23.3 ($CH_3$, $^i$Pr), 18.8 ($CH_3$, Me), 17.8 ($CH_3$, $C_6H_4$Me) ppm

**IR spectrum:** ν 3194.1 (s, NH stretching), 3048.9 (broad s, NH stretching), 1646.0 (s, C=O stretching) $cm^{-1}$

**Melting point analysis**: 140-142 °C

Figure S4: ORTEP diagram of [(MeCNH-Dipp)(MeCN-Dipp)C(2-MeC$_6$H$_4$NHCO)] (**4**)

Molecular structure of **4**, with selected hydrogen atoms displayed and organic groups shown as wire frame for clarity. Thermal ellipsoids are displayed at 40 % probability level.

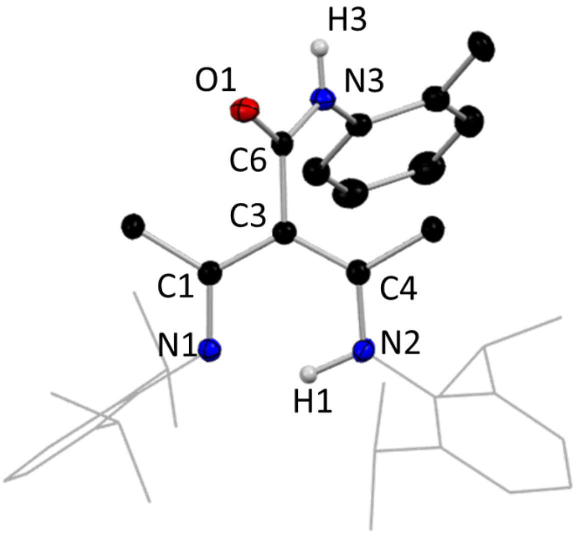


Table S6: Selected bond lengths (Å) and bond angles (°) of **4**

| Atoms | Bond Lengths (Å) |
|---|---|
| N1-C1 | 1.312(5) |
| C1-C3 | 1.439(5) |
| C3-C4 | 1.407(5) |
| C4-N2 | 1.338(5) |
| C3-C6 | 1.496(5) |
| C6-N3 | 1.365(5) |
| C6-O1 | 1.237(4) |
| **Atoms** | **Bond Angles (°)** |
| C1-C3-C6 | 117.6(3) |
| C4-C3-C6 | 119.3(3) |
| O1-C6-N3 | 119.3(3) |

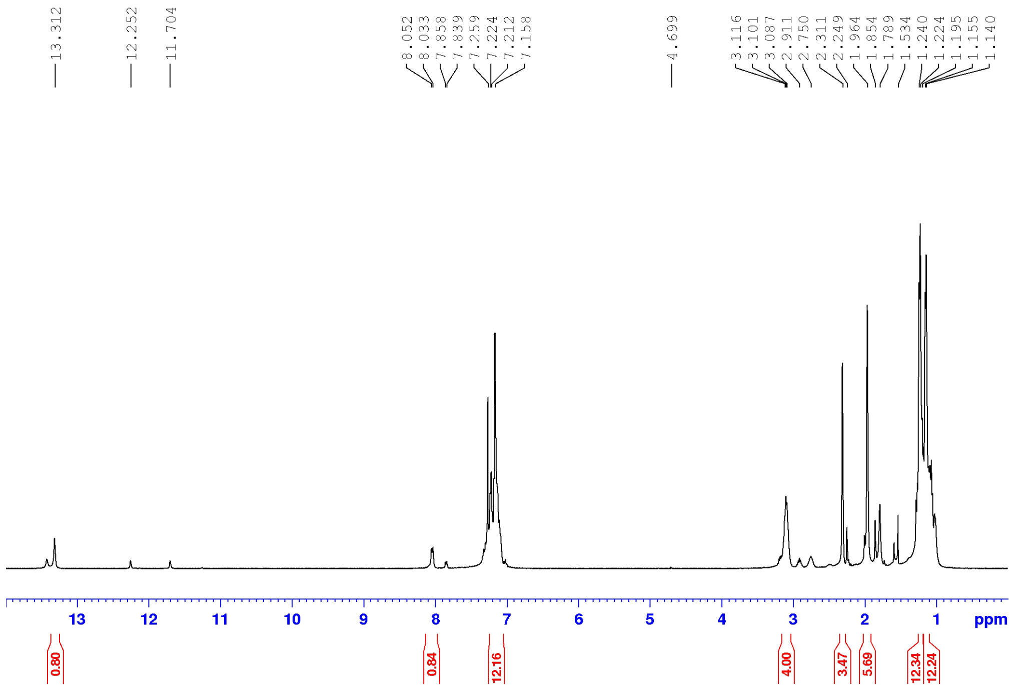


**Figure S4.1**: $^{1}H$ NMR spectrum in $C_6D_6$ of **4** [(MeCNH-Dipp)(MeCN-Dipp)C(2-$MeC_6H_4$NHCO)]

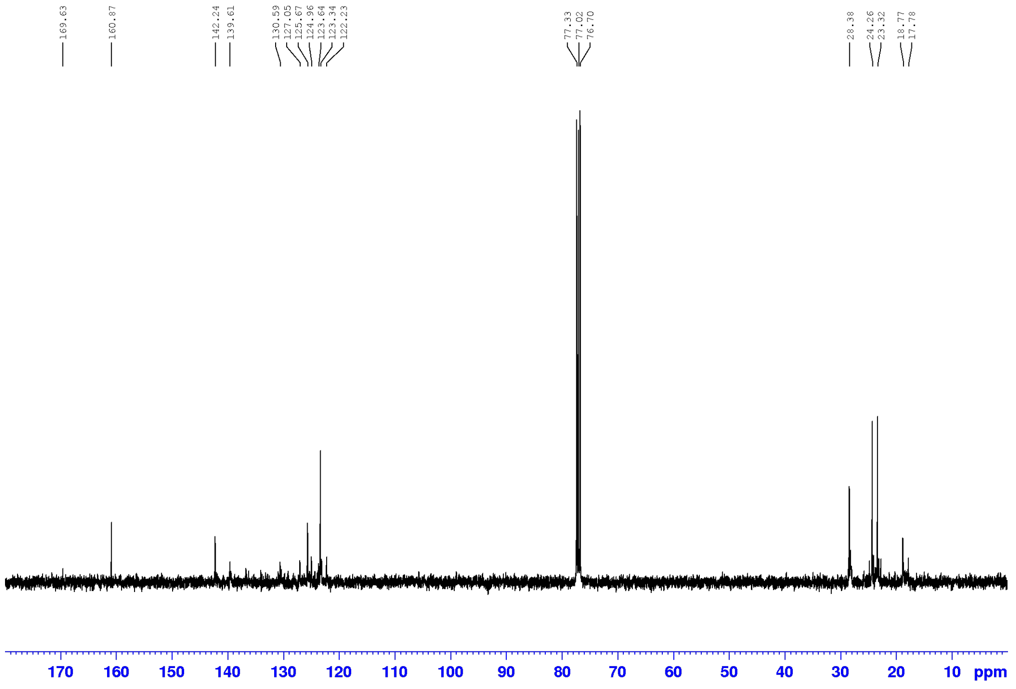


**Figure S4.2**: $^{13}C\{^{1}H\}$ NMR spectrum in $C_6D_6$ of **4** [(MeCNH-Dipp)(MeCN-Dipp)C(2-$MeC_6H_4$NHCO)]

## Synthesis of [(MeCNH-Dipp)(MeCN-Dipp)C(2,6-$Me_2C_6H_3$NHCO)] (**5**)

NacNacLi (0.85 g, 2.0 mmol) dissolved in hexane (10 ml) to produce a yellow solution before 2,6-$Me_2$NCO (0.28 ml, 2.0 mmol) was added giving an off-white suspension. Suspension stirred (3 hrs) before deionised water (5 ml) was added and mixture exposed to air and stirred overnight. Separation performed using DCM (15 ml), organic layer dried using $MgSO_4$ before product was filtered, dried, and crystallised from methanol. Product was collected as off-white/yellow crystals (0.67 g, 1.15 mmol, 58.0 %).

**$^1$H NMR (400.1 MHz, $CDCl_3$, 300 K): δ** 13.49 (s, 1H, NH, NH), 10.80 (unknown impurity), 7.26 ($CDCl_3$), 7.18-7.05 (broad m, 14H, CH, $C_6H_3$/$C_6H_4$), 6.98 (m, 1H, CH, $C_6H_3$), 3.09 (broad m, 4H, CH, $^i$Pr), 2.35 (s, 6H, $CH_3$, $C_6H_3$Me), 2.03 (s, 6H, $CH_3$, $C_6H_3$Me), 1.53 (s, 6H, $CH_3$, Me), 1.22 (d, 16H, $CH_3$, $^i$Pr), 1.15 (d, 16H, $CH_3$, $^i$Pr) ppm

Residual NacNacH: 12.17 (NH), 4.78 (γ-CH), 3.17 (CH, $^i$Pr), 2.94 (CH, $^i$Pr), 1.84 ($CH_3$, Me), 1.26 ($CH_3$, $^i$Pr), 1.05 ($CH_3$, $^i$Pr) ppm

**$^{13}$C{$^1$H} NMR (100.6 MHz, $CDCl_3$, 300 K): δ** 161.0 ($C_{quaternary}$, CMe), 142.2 ($C_{quaternary}$, $C_6H_3$), 128.7 (CH, $C_6H_3$), 125.6 (CH, $C_6H_3$), 123.3 (CH, $C_6H_3$), 77.0 ($CDCl_3$), 28.3 (CH, $^i$Pr), 24.3 ($CH_3$, $^i$Pr), 23.4 ($CH_3$, $^i$Pr), 19.4 ($CH_3$, Me), 19.2 ($CH_3$, $C_6H_3$Me) ppm

**IR spectrum:** ν 3242.2 (broad m, NH stretching), 1690.3 (s, C=O stretching) $cm^{-1}$

**Melting point analysis**: 119-121 °C

## Figure S5: ORTEP diagram of [(MeCNH-Dipp)(MeCN-Dipp)C(2,6-$Me_2C_6H_3$NHCO)] (**5**)

Molecular structure of **5**, with selected hydrogen atoms displayed and organic groups shown as wire frame for clarity. Thermal ellipsoids are displayed at 40 % probability level. Co-crystallised solvent omitted, and second molecule not shown.

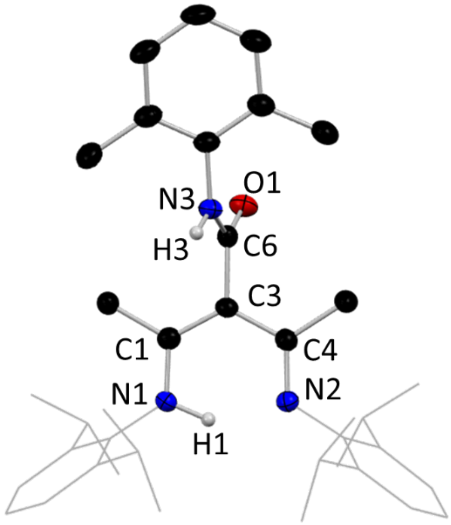


Table S7: Selected bond lengths (Å) and bond angles (°) of **5**

| Atoms | Bond Lengths (Å) |
|---|---|
| N1-C1 | 1.352(3) |
| C1-C3 | 1.386(3) |
| C3-C4 | 1.454(3) |
| C4-N2 | 1.296(3) |
| C3-C6 | 1.507(3) |
| C6-N3 | 1.337(3) |
| C6-O1 | 1.240(3) |
| N3-C31 | 1.443(3) |
| **Atoms** | **Bond Angles (°)** |
| C4-C3-C1 | 123.67(18) |
| C1-C3-C6 | 118.12(18) |
| C4-C3-C6 | 118.00(18) |
| C3-C6-O1 | 122.44(18) |
| O1-C6-N3 | 120.24(19) |
| C3-C6-N3 | 117.32(18) |
| C6-N3-C31 | 122.42(19) |

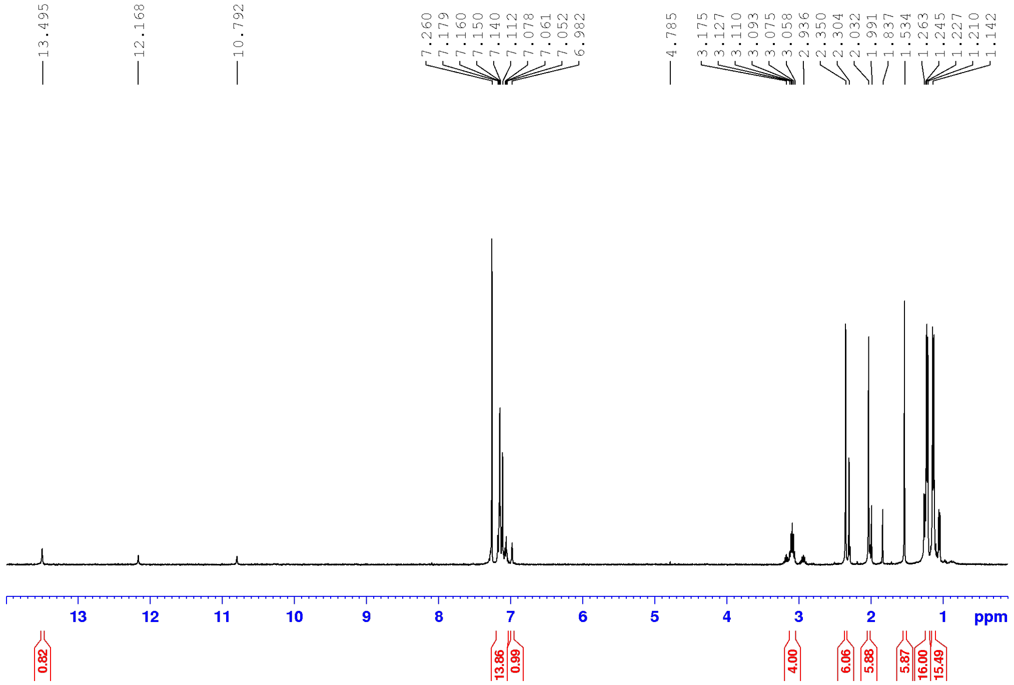


**Figure S5.1**: $^{1}$H NMR spectrum in $C_6D_6$ of **5** [(MeCNH-Dipp)(MeCN-Dipp)C(2,6-$Me_2C_6H_3$NHCO)]

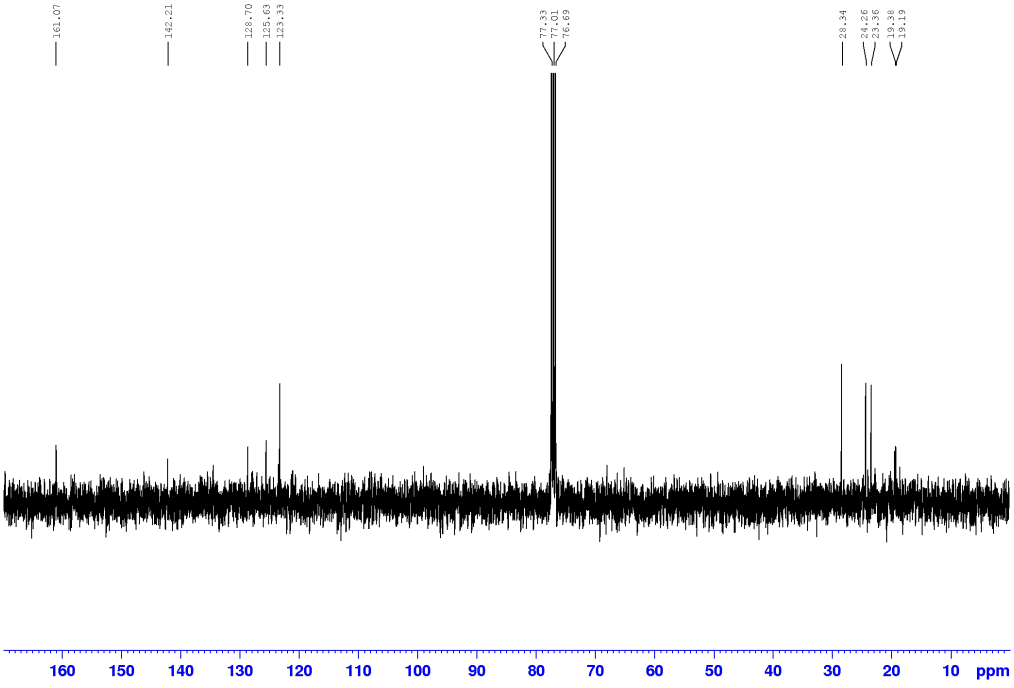


**Figure S5.2**: $^{13}C\{^{1}H\}$ NMR spectrum in $C_6D_6$ of **5** [(MeCNH-Dipp)(MeCN-Dipp)C(2,6-$Me_2C_6H_3$NHCO)]

## Synthesis of [(MeCNH-Dipp)(MeCN-Dipp)C(CyNHCO)] (**6**)

NacNacLi (0.86 g, 2.0 mmol) dissolved in hexane (10 ml) to produce a yellow solution before CyNCO (0.26 ml, 2.0 mmol) was added giving an off-white suspension. Suspension stirred (3 hrs) before deionised water (5 ml) was added and mixture exposed to air and stirred overnight. Separation performed using DCM (15 ml), organic layer dried using $MgSO_4$ before product was filtered, dried, and crystallised from methanol. Product was collected as off-white crystals (0.87 g, 1.60 mmol, 80.3 %).

**$^1$H NMR (400.1 MHz, $CDCl_3$, 300 K): δ** 13.08 (s, 1H, NH, NH), 7.26 ($CDCl_3$), 7.13 (broad m, 6H, CH, $C_6H_3$), 5.56 (s, 1H, NH, C(=O)NH), 3.90 (m, 1H, CH, Cy), 3.35 (sep, 4H, CH, $^i$Pr), 3.49 (MeOH), 2.02 (m, 2H, $CH_2$, Cy), 1.90 (m, 1H, $CH_2$, Cy), 1.82 (s, 6H, $CH_3$, Me), 1.74 (m, 2H, $CH_2$, Cy), 1.62 (broad m, 3H, $CH_2$, Cy), 1.40 (m, 2H, $CH_2$, Cy), 1.20 (d, 13H, $CH_3$, $^i$Pr), 1.11 (d, 12H, $CH_3$, $^i$Pr), 0.96 (hexane) ppm

Residual NacNacH: 12.05 (NH), 4.52 (γ-CH), 1.25-1.16 ($CH_3$, $^i$Pr) ppm

**$^{13}$C{$^1$H} NMR (100.6 MHz, $CDCl_3$, 300 K): δ** 170.5 ($C_{quaternary}$, C=O), 160.4 ($C_{quaternary}$, CMe), 142.5 ($C_{quaternary}$, $^i$Pr), 139.9 ($C_{quaternary}$, $C_6H_3$), 126.1 (CH, $C_6H_3$), 123.7 (CH, $C_6H_3$), 107.8 ($C_{quaternary}$, γ-C), 70.16 ($CDCl_3$), 48.8 (CH, Cy), 33.3 ($CH_2$, Cy), 28.6 (CH, $^i$Pr), 28.4 (CH, $^i$Pr), 25.7 ($CH_2$, Cy), 25.1 ($CH_2$, Cy), 24.4 ($CH_3$, $^i$Pr), 23.5 ($CH_3$, $^i$Pr), 18.5 ($CH_3$, Me) ppm

**IR spectrum:** ν 3193.5 (broad s, NH stretching), 3077.4 (s, NH stretching), 1645.9 (s, C=O stretching) $cm^{-1}$

**Melting point analysis**: 145-147 °C

Figure S6: ORTEP diagram of [(MeCNH-Dipp)(MeCN-Dipp)C(CyNHCO)] (**6**)

Molecular structure of **6**, with selected hydrogen atoms displayed and organic groups shown as wire frame for clarity. Thermal ellipsoids are displayed at 40 % probability level. Co-crystallised solvent omitted, and second molecule not shown.

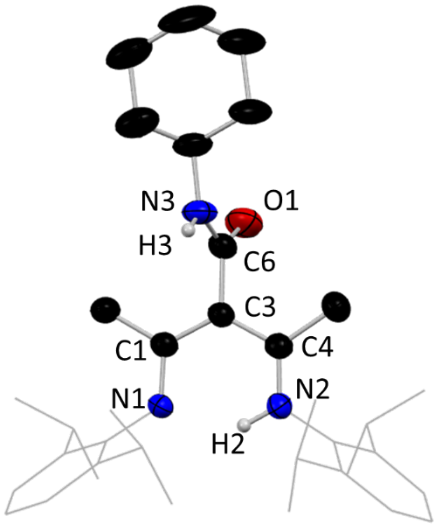


Crystallographic data of compound **6** were of insufficient quality to allow meaningful discussion of bond lengths or bond angles.

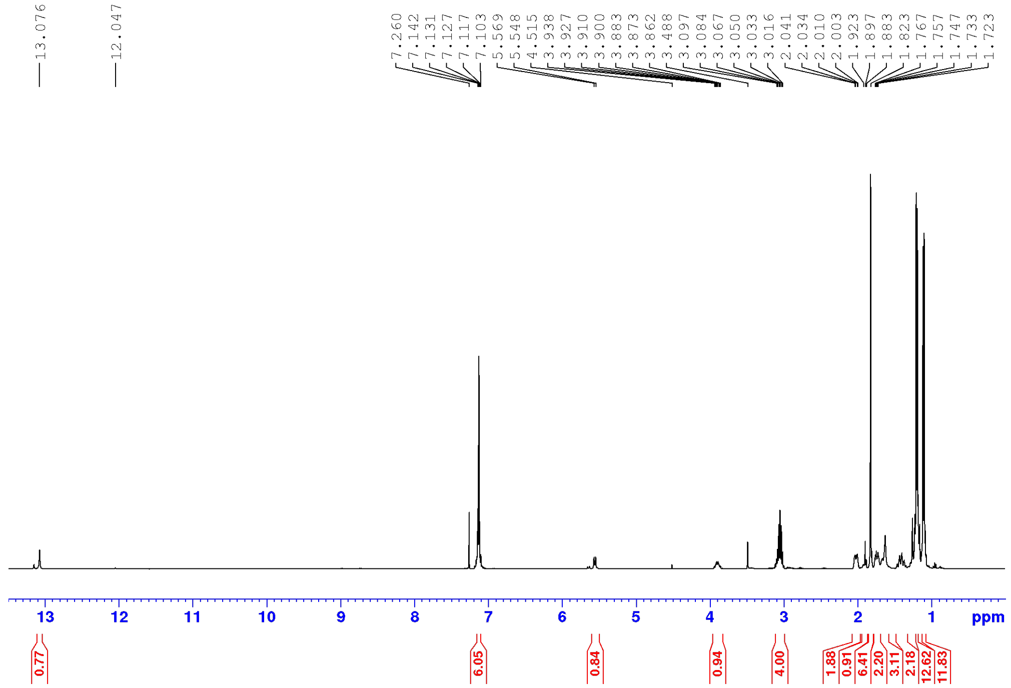


**Figure S6.1**: $^{1}H$ NMR spectrum in $CDCl_3$ of **6** [(MeCNH-Dipp)(MeCN-Dipp)C(CyNHCO)]

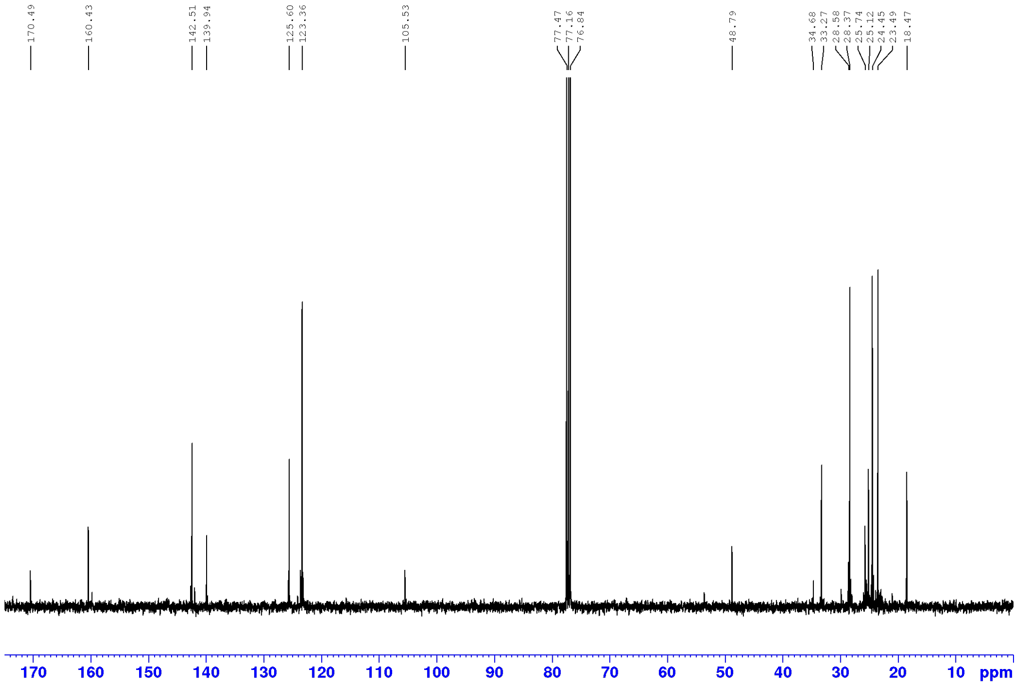


**Figure S6.2**: $^{13}C\{^{1}H\}$ NMR spectrum in $CDCl_3$ of **6** [(MeCNH-Dipp)(MeCN-Dipp)C(CyNHCO)]

## Synthesis of [(MeCNH-Dipp)(MeCN-Dipp)C(PhNHCO)] (**7**)

NacNacLi (0.86 g, 2.0 mmol) dissolved in hexane (10 ml) to produce a yellow solution before PhNCO (0.22 ml, 2.0 mmol) was added giving a yellow suspension. Suspension stirred (3 hrs) before deionised water (5 ml) was added and mixture exposed to air and stirred overnight. Separation performed using DCM (15 ml), organic layer dried using $MgSO_4$ before product was filtered, dried, and crystallised from methanol (0.91 g, 1.67 mmol, 83.4 %).

**$^1$H NMR (400.1 MHz, $CDCl_3$, 300 K): δ** 13.34 (s, 1H, NH, NH), 11.72 (unknown impurity), 7.63 (m, 2H, CH, $C_6H_3$), 7.56 (m, 1H, CH, $C_6H_3$), 7.37 (broad m, 3H, CH, $C_6H_3$), 7.26 ($CDCl_3$), 7.15 (broad m, 7H, CH, $C_6H_3$), 3.08 (m, 4H, CH, $^i$Pr), 1.92 (s, 5H, $CH_3$, Me), 1.81 (unknown impurity), 1.60 ($H_2O$), 1.23 (d, 9H, $CH_3$, $^i$Pr), 1.14 (d, 12H, $CH_3$, $^i$Pr), 1.10 (MeOH), 0.89 (hexane) ppm

Residual NacNacH: 12.22 (NH), 4.67 (γ-CH), 3.17 (CH, $^i$Pr), 2.91 (CH, $^i$Pr), 2.80 (CH, $^i$Pr), 1.95 ($CH_3$, Me), 1.84 ($CH_3$, Me), 1.27 ($CH_3$, $^i$Pr), 1.20 ($CH_3$, $^i$Pr) ppm

**$^{13}$C{$^1$H} NMR (100.6 MHz, $CDCl_3$, 300 K): δ** 169.8 ($C_{quaternary}$, C=O), 161.1 ($C_{quaternary}$, CMe), 146.6 ($C_{quaternary}$, $C_6H_3$), 142.4 ($C_{quaternary}$, $C_6H_3$), 139.7 ($C_{quaternary}$, $C_6H_3$), 138.5 ($C_{quaternary}$, $C_6H_3/C_6H_4$), 136.9 (CH, $C_6H_3/C_6H_4$), 129.3 (CH, $C_6H_3/C_6H_4$), 128.8 (CH, $C_6H_3/C_6H_4$), 125.8 (CH, $C_6H_3/C_6H_4$), 124.5 (CH, $C_6H_3/C_6H_4$), 124.0 (CH, $C_6H_3/C_6H_4$), 123.8 (CH, $C_6H_3/C_6H_4$), 123.6 (CH, Ph), 123.5 (CH, $C_6H_3/C_6H_4$), 122.6 (CH, $C_6H_3/C_6H_4$), 119.8 (CH, $C_6H_3/C_6H_4$), 105.7 ($C_{quaternary}$, γ-C ), 77.2 ($CDCl_3$), 28.5 (CH, $^i$Pr), 24.5 ($CH_3$, $^i$Pr), 23.5 ($CH_3$, $^i$Pr), 18.8 ($CH_3$, Me) ppm

Residual NacNacH: 120.8 (CH, $C_6H_3$), 128.4 (CH, $C_6H_3$), 28.6 (CH, $^i$Pr), 24.9 ($CH_3$, $^i$Pr), 22.9 ($CH_3$, $^i$Pr), 20.2 ($CH_3$, Me) ppm

**IR spectrum:** ν 3197.4 (s, NH stretching), 3069.7 (m, NH stretching), 1652.2 (s, C=O stretching) $cm^{-1}$

**Melting point analysis**: 141-143 °C

A second crystallographic dataset of compound **7** was collected, **7.1**, which contains a co-crystallised THF molecule within the asymmetric unit.

Figure S7: ORTEP diagram of [(MeCNH-Dipp)(MeCN-Dipp)C(PhNHCO)] (**7**)

Molecular structure of **7**, with selected hydrogen atoms displayed and organic groups shown as wire frame for clarity. Thermal ellipsoids are displayed at 40 % probability level. Second molecule and co-crystallised solvent not shown.

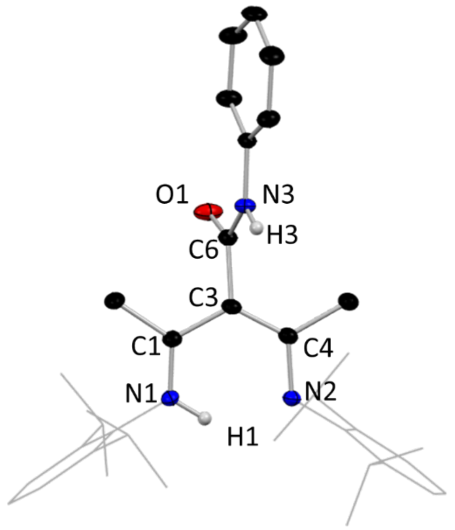


Table S8: Selected bond lengths (Å) and bond angles (°) of **7**

| Atoms | Bond Lengths (Å) |
|---|---|
| N1-C1 | 1.2951(14) |
| C1-C3 | 1.4608(16) |
| C3-C4 | 1.3801(16) |
| C4-N2 | 1.3571(14) |
| C3-C6 | 1.4998(15) |
| C6-N3 | 1.3522(15) |
| C6-O1 | 1.2350(15) |
| N3-C7 | 1.4225(14) |
| **Atoms** | **Bond Angles (°)** |
| C4-C3-C1 | 123.87(10) |
| C1-C3-C6 | 115.46(10) |
| C4-C3-C6 | 120.17(10) |
| C3-C6-O1 | 120.04(10) |
| O1-C6-N3 | 122.60(10) |
| C3-C6-N3 | 117.33(10) |
| C6-N3-C7 | 127.68(10) |

Molecular structure of **7.1**, showing **7** with THF co-crystallised, with selected hydrogen atoms displayed and organic groups shown as wire frame for clarity. Thermal ellipsoids are displayed at 40 % probability level. Second molecule not included.

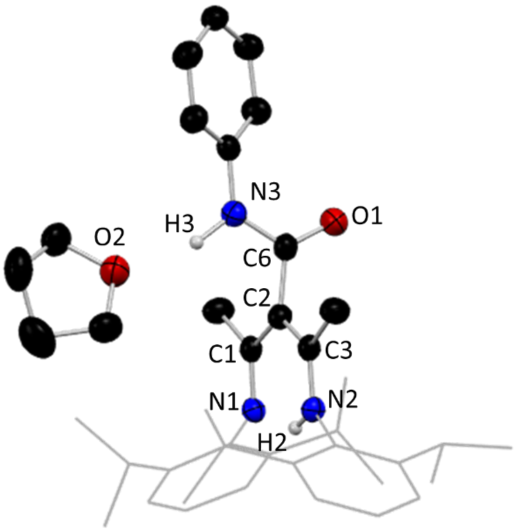

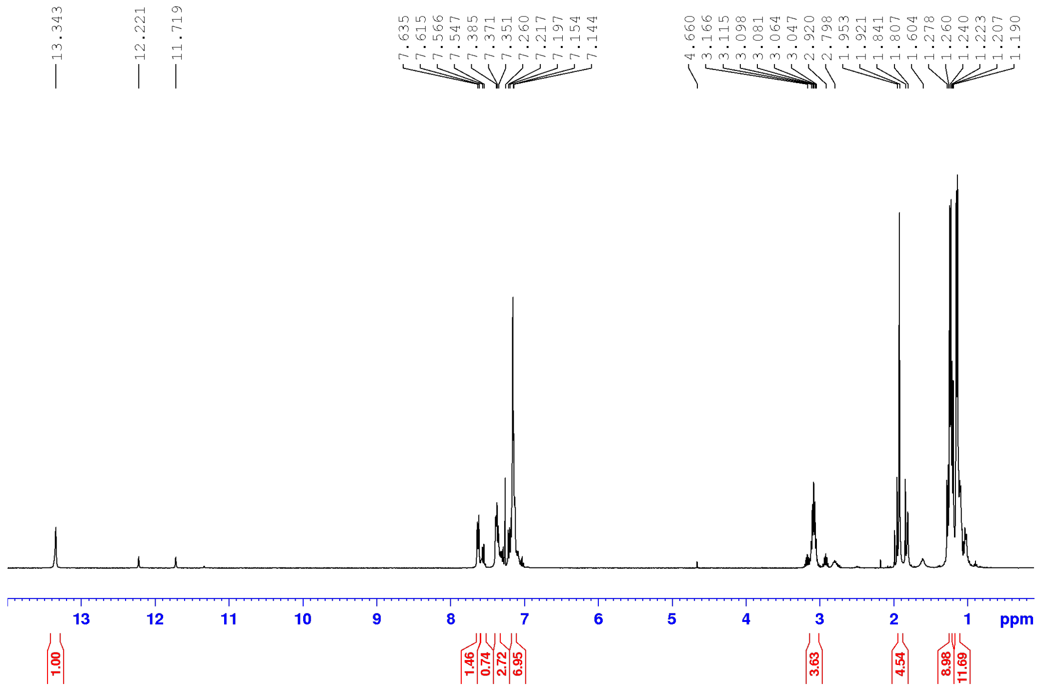


**Figure S7.1**: $^{1}H$ NMR spectrum in $CDCl_3$ of **7** [(MeCNH-Dipp)(MeCN-Dipp)C(PhNHCO)]

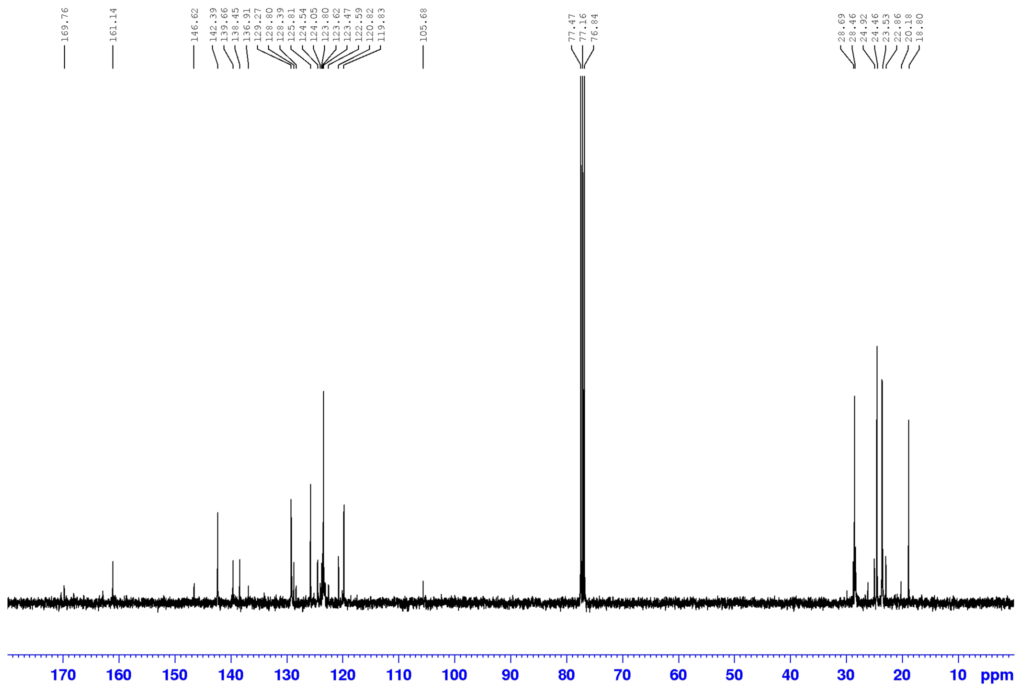


**Figure S7.2**: $^{13}C\{^{1}H\}$ NMR spectrum in $CDCl_3$ of **7** [(MeCNH-Dipp)(MeCN-Dipp)C(PhNHCO)]

## Synthesis of [{(MeCN-Dipp)$_2$C(4-MeC$_6$H$_4$NHCO)}Li·2THF] (**7a**)

NacNacLi (0.043 g, 0.1 mmol) placed in vial, dissolved in 1 ml hexane, PhNCO (11 µl, 0.1 mmol) added, yellow suspension. THF (1 ml) added to produce a pale-yellow solution, placed in -20 °C freezer, and a small batch of colourless crystals formed overnight (0.030 g, 0.04 mmol, 43.5 %).

**$^1$H NMR (400.1 MHz, C$_6$D$_6$, 300 K): δ** 13.44 (s, 1H, NH, C(=O)NH), 7.22 (m, 2H, CH, C$_6$H$_3$), 7.16 (C$_6$D$_6$), 7.09 (broad m, 9H, CH, C$_6$H$_3$), 7.03 (m, 3H, CH, C$_6$H$_3$), 6.90 (m, 2H, CH, Ph), 6.80 (m, 1H, CH, Ph), 3.89 (m, 5H, CH$_2$, THF), 3.17 (m, 2H, CH, $^i$Pr), 2.79 (m, 2H, CH, $^i$Pr), 2.08 (s, 6H, CH$_3$, Me), 1.53 (m, 5H, CH$_2$, THF), 1.12 (d, 6H, CH$_3$, $^i$Pr), 1.07 (d, 7H, CH$_3$, $^i$Pr), 1.01 (dd, 12H, CH$_3$, $^i$Pr), 0.89 (hexane) ppm

**$^{13}$C{$^1$H} NMR (100.6 MHz, C$_6$D$_6$, 300 K): δ** 160.3 (C$_{quaternary}$, C=O), 150.1 (C$_{quaternary}$, CMe), 142.5 (C$_{quaternary}$, C$_6$H$_3$), 141.8 (C$_{quaternary}$, C$_6$H$_3$), 140.1 (C$_{quaternary}$, Ph), 128.9 (CH, C$_6$H$_3$), 128.8 (CH, C$_6$H$_3$), 128.6 (CH, C$_6$H$_3$), 128 (C$_6$D$_6$), 125.5 (CH, Ph), 123.6 (CH, Ph), 123.5 (CH, Ph), 123.1 (CH, Ph), 67.6 (CH$_2$, THF), 28.1 (CH, $^i$Pr), 27.7 (CH, $^i$Pr), 25.5 (CH$_2$, THF), 24.4 (CH$_3$, $^i$Pr), 24.3 (CH$_3$, $^i$Pr), 23.6 (CH$_3$, $^i$Pr), 23.2 (CH$_3$, $^i$Pr), 19.5 (CH$_3$, Me) ppm

**$^7$Li NMR (155.5 MHz, C$_6$D$_6$, 298K) δ** 1.76 ppm

**IR spectrum:** ν 3079.3 (s, NH stretching), 1631.5 (s, C=O stretching) cm$^{-1}$

**Melting point analysis**: 159-161 °C

Figure S7a: ORTEP diagram of [{(MeCN-Dipp)$_2$C(4-MeC$_6$H$_4$NHCO)}Li·2THF] (**7a**)

Molecular structure of **7a**, with selected hydrogen atom displayed and organic groups shown as wire frame for clarity. Thermal ellipsoids are displayed at 40 % probability level. Second molecule not included.

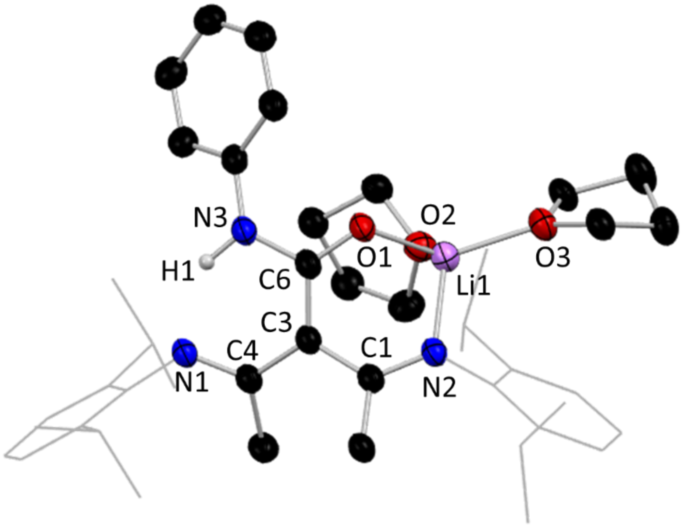

Table S9: Selected bond lengths (Å) and bond angles (°) of **7a**

| **Atoms** | **Bond Lengths (Å)** |
|---|---|
| Li1-O1 | 1.889(7) |
| Li1-O2 | 1.967(7) |
| Li1-O3 | 1.957(7) |
| Li1-N1 | 2.013(7) |
| N1-C1 | 1.299(4) |
| C1-C3 | 1.464(5) |
| C3-C4 | 1.454(5) |
| C4-N2 | 1.310(5) |
| C3-C6 | 1.451(5) |
| C6-N3 | 1.376(5) |
| C6-O1 | 1.267(4) |
| N3-C7 | 1.407(5) |
| **Atoms** | **Bond Angles (°)** |
| N1-Li1-O1 | 89.2(3) |
| N1-Li1-O2 | 117.6(3) |
| N1-Li1-O3 | 115.1(3) |
| O1-Li1-O2 | 102.7(3) |
| O1-Li1-O3 | 137.6(4) |
| O2-Li1-O3 | 95.9(3) |
| C4-C3-C1 | 119.0(3) |
| C1-C3-C6 | 118.2(3) |
| C4-C3-C6 | 122.8(3) |
| C3-C6-O1 | 124.5(3) |
| O1-C6-N3 | 118.5(3) |
| C3-C6-N3 | 117.0(3) |
| C6-N3-C7 | 126.0(3) |

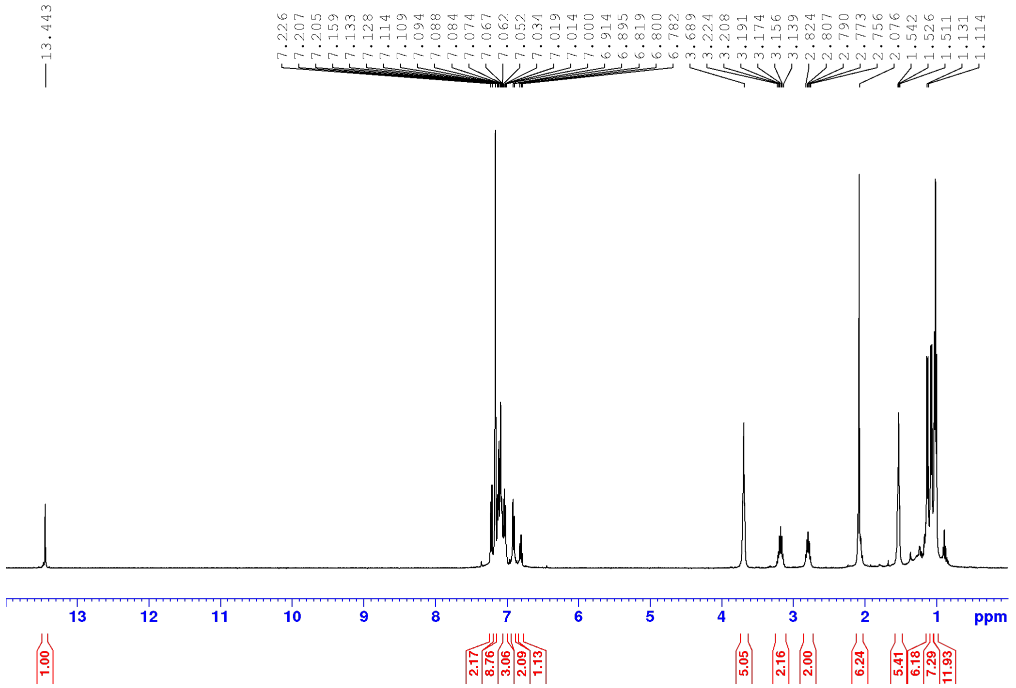

Figure S7a.1: $^{1}H$ NMR spectrum in $C_6D_6$ of **7a** [{(MeCN-Dipp)$_2$C(4-MeC$_6$H$_4$NHCO)}Li·2THF]

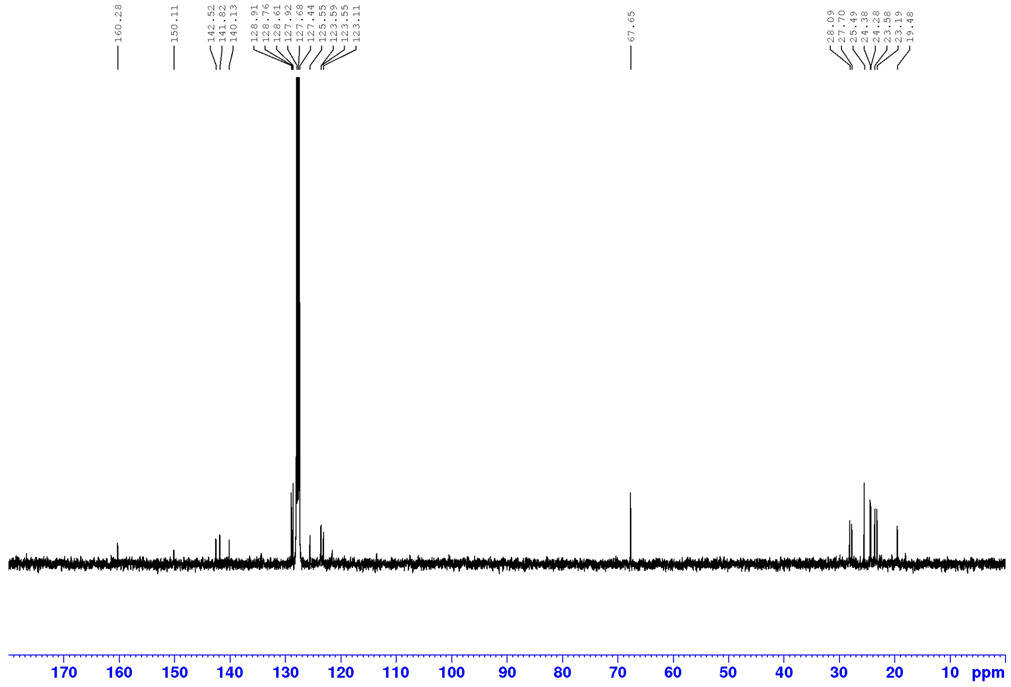

**Figure S7a.2**: $^{13}C\{^{1}H\}$ NMR spectrum in $C_6D_6$ of **7a** [{(MeCN-Dipp)$_2$C(4-MeC$_6$H$_4$NHCO)}Li·2THF]

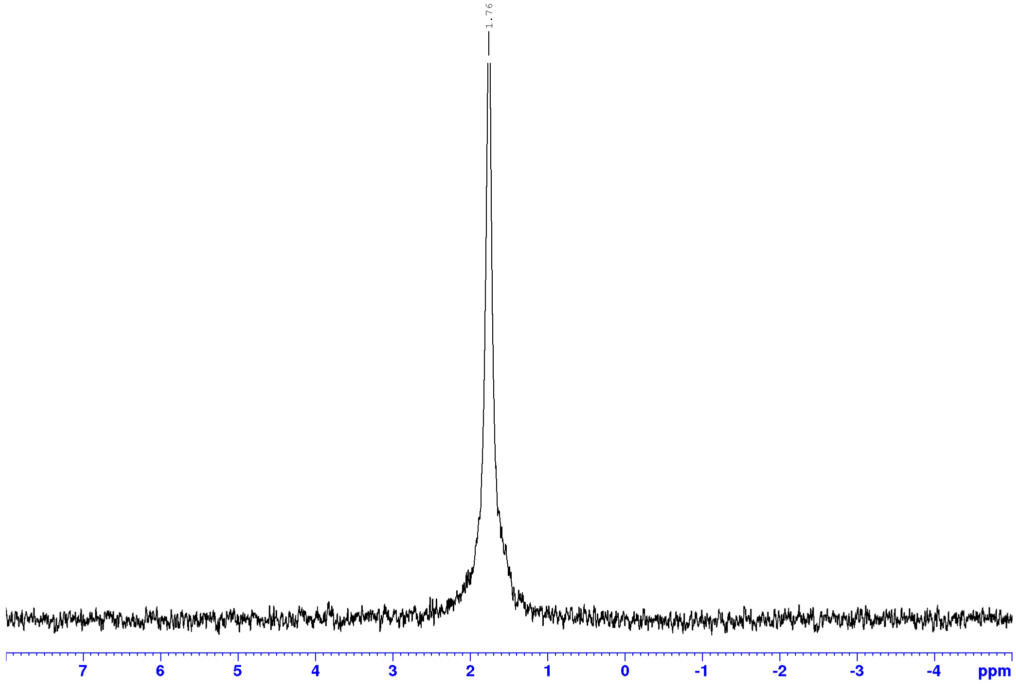


**Figure S7a.3**: $^{7}Li$ NMR spectrum in $C_6D_6$ of **7a** [{(MeCN-Dipp)$_2$C(4-Me$C_6H_4$NHCO)}Li·2THF]

## Synthesis of [{(MeCN-Dipp)(MeCNH-Dipp)C(PhNCO)}Li]$_4$·2THF (**7b**)

$^{Ph}$NacNacLi (0.27 g, 0.5 mmol) placed in vial, dissolved in 5 ml toluene, THF (2 ml) added to produce a pale-yellow solution, and a batch of large colourless crystals formed over the course of several weeks by slow evaporation (0.09 g, 0.16 mmol, 31.0 %).

**$^{1}$H NMR (400.1 MHz, $C_6D_6$, 300 K): δ** 13.53 (s, 1H, NH, C(=O)NH), 7.24 (m, 2H, CH, $C_6H_3$), 7.19 (broad m, 3H, CH, $C_6H_3$), 7.16 ($C_6D_6$), 7.14 (toluene), 7.10 (toluene), 6.98 (m, 2H, CH, Ph), 6.88 (m, 1H, CH, Ph), 3.92 (m, 2H, $CH_2$, THF), 3.25 (m, 2H, CH, $^{i}$Pr), 2.86 (m, 2H, CH, $^{i}$Pr), 2.19 (toluene), 2.15 (s, 6H, $CH_3$, Me), 1.76 (m, 3H, $CH_2$, THF), 1.20 (d, 7H, $CH_3$, $^{i}$Pr), 1.15 (d, 7H, $CH_3$, $^{i}$Pr), 1.09 (dd, 12H, $CH_3$, $^{i}$Pr) ppm

**$^{13}$C{$^{1}$H} NMR (100.6 MHz, $C_6D_6$, 300 K): δ** 177.1 ($C_{quaternary}$, C=O), 160.6 ($C_{quaternary}$, CMe), 150.3 ($C_{quaternary}$, $C_6H_3$), 142.8 ($C_{quaternary}$, $C_6H_3$), 142.1 ($C_{quaternary}$, Ph), 140.4 ($C_{quaternary}$, Ph), 137.9 (toluene), 129.3 (CH, $C_6H_3$), 128.5 (toluene), 128.4 (toluene), 128 ($C_6D_6$), 125.9 (CH, $C_6H_3$), 125.6 (CH, $C_6H_3$), 123.9 (CH, Ph), 123.8 (CH, Ph), 123.4 (CH, Ph), 121.9 (CH, Ph), 106.2 ($C_{quaternary}$, γ-C), 68.2 ($CH_2$, THF), 28.4 (CH, $^{i}$Pr), 28.0 (CH, $^{i}$Pr), 25.9 ($CH_2$, THF), 24.7 ($CH_3$, $^{i}$Pr), 24.6 ($CH_3$, $^{i}$Pr), 23.9 ($CH_3$, $^{i}$Pr), 23.5 ($CH_3$, $^{i}$Pr), 21.4 (toluene), 19.8 ($CH_3$, Me) ppm

**$^{7}$Li NMR (155.5 MHz, $C_6D_6$, 298K) δ** 1.76 ppm

Due to the NMR spectroscopic analysis of **7a** and **7b** varying only in their THF ratios, further analysis of **7b** was considered to be unnecessary.

Figure S7b: ORTEP diagram of [{(MeCN-Dipp)(MeCNH-Dipp)C(PhNCO)}Li]$_4$·2THF (**7b**)

Molecular structure of **7b**, with organic groups shown as wire frame for clarity. Thermal ellipsoids are displayed at 40 % probability level. Solvent molecule omitted.

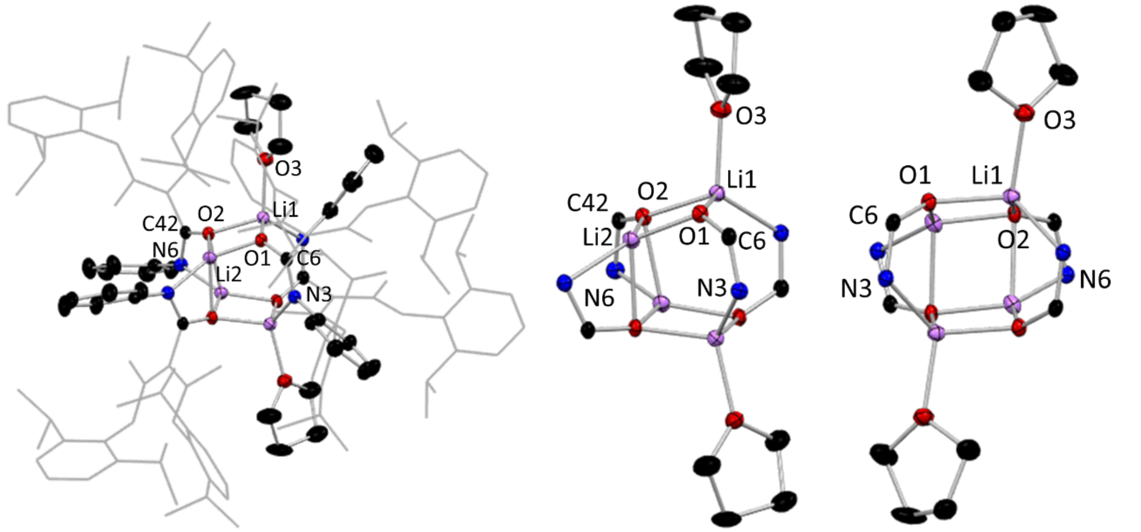

Table S10: Selected bond lengths (Å) and bond angles (°) of **7b**

| **Atoms** | **Bond Lengths (Å)** |
|---|---|
| Li1-O1 | 1.924(5) |
| Li1-O2 | 1.953(5) |
| Li1-O3 | 1.987(5) |
| Li1-N3’ | 2.055(5) |
| O1-C6 | 1.296(3) |
| C6-N3 | 1.306(4) |
| C6-C3 | 1.503(4) |
| C3-C1 | 1.387(4) |
| C1-N1 | 1.353(4) |
| C3-C4 | 1.447(4) |
| C4-N2 | 1.300(4) |
| Li2-O1 | 1.883(5) |
| Li2-O2 | 1.894(5) |
| Li2-O2’ | 2.109(5) |
| Li2-N6’ | 2.006(5) |
| O2-C42 | 1.304(3) |
| C42-N6 | 1.299(4) |
| C42-C39 | 1.502(4) |
| C39-C37 | 1.382(4) |
| C37-N4 | 1.353(4) |
| C40-N5 | 1.315(4) |
| **Atoms** | **Bond Angles (°)** |
| N3’-Li1-O1 | 120.7(2) |
| N3’-Li1-O2 | 110.4(2) |
| N3’-Li1-O3 | 116.9(2) |
| O1-Li1-O2 | 94.1(2) |
| O1-Li1-O3 | 102.1(2) |
| O2-Li1-O3 | 110.2(2) |
| N6’-Li2-O1 | 135.4(3) |
| N6’-Li2-O2 | 127.0(3) |
| N6’-Li2-O2’ | 65.91(17) |
| O1-Li2-O2 | 97.4(2) |
| O1-Li2-O2’ | 110.3(2) |
| O2-Li2-O2’ | 97.2(2) |
| O2-C42-C39 | 117.6(2) |
| O2-C42-N6 | 118.7(2) |
| N6-C42-C39 | 123.6(2) |
| C42-N6-C43 | 121.2(2) |
| O1-C6-C3 | 116.2(2) |
| O1-C6-N3 | 120.1(2) |
| N3-C6-C3 | 123.6(2) |
| C6-N3-C7 | 121.3(2) |

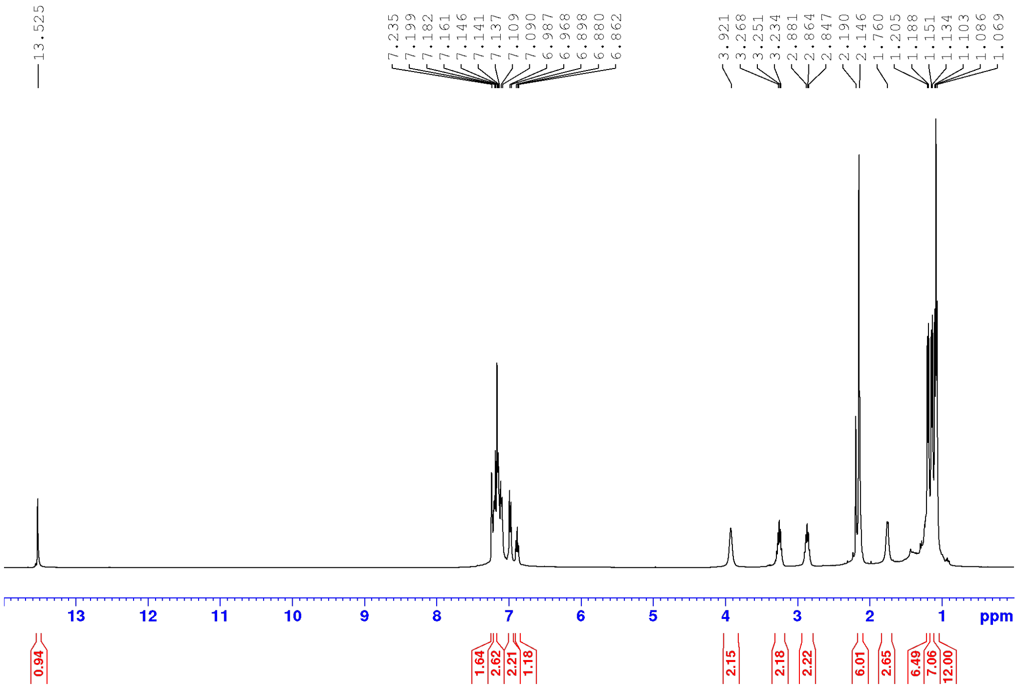


**Figure S7b.1**: $^{1}H$ NMR spectrum in $C_6D_6$ of **7b** [{(MeCN-Dipp)(MeCNH-Dipp)C(PhNCO)}Li]$_4$·2THF

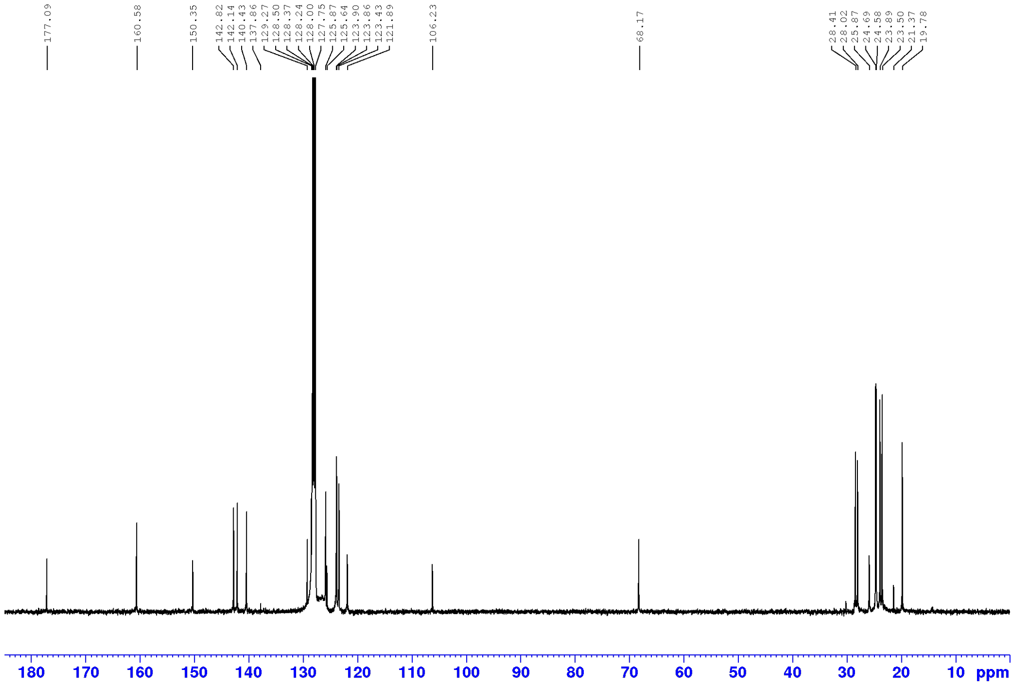


**Figure S7b.2**: $^{13}C\{^{1}H\}$ NMR spectrum in $C_6D_6$ of **7b** [{(MeCN-Dipp)(MeCNH-Dipp)C(PhNCO)}Li]$_4$·2THF

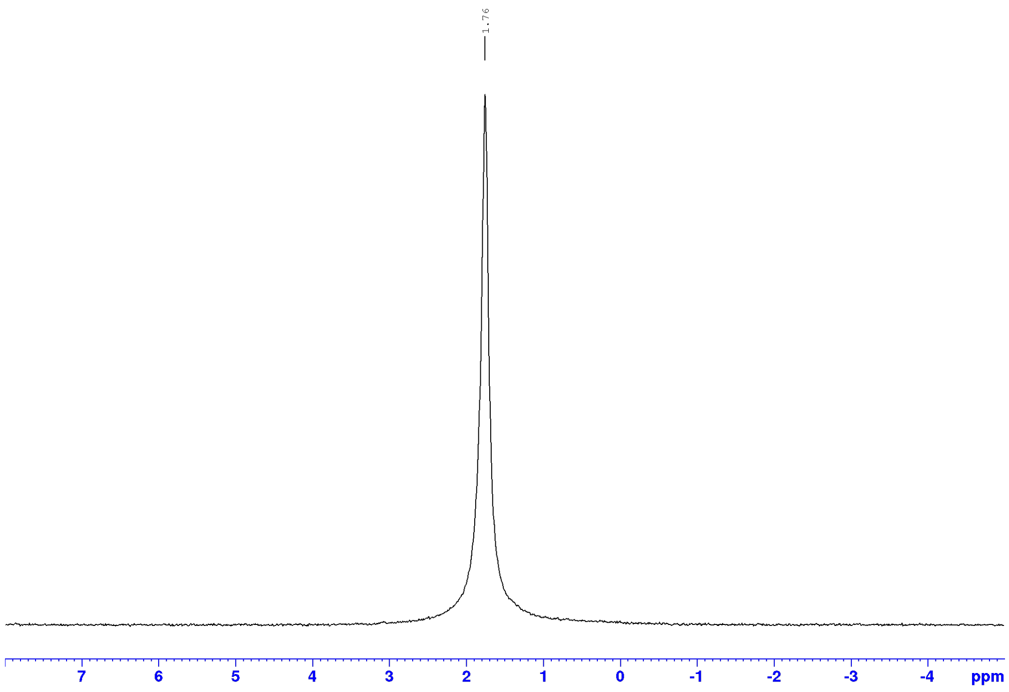


**Figure S7b.3**: $^{7}$Li NMR spectrum in $C_6D_6$ of **7b** [{(MeCN-Dipp)(MeCNH-Dipp)C(PhNCO)}Li]$_4$·2THF

## Synthesis of [(MeCNH-Dipp)(MeCN-Dipp)C($^i$PrNHCO)] (**8**)

NacNacLi (0.86 g, 2.0 mmol) dissolved in hexane (10 ml) to produce a yellow solution before $^i$PrNCO (0.20 ml, 2.0 mmol) was added giving an off-white suspension. Suspension stirred (3 hrs) before deionised water (5 ml) was added and mixture exposed to air and stirred overnight. Separation performed using DCM (15 ml), organic layer dried using $MgSO_4$ before product was filtered, dried, and crystallised from methanol. Product was collected as off-white crystals (0.89 g, 1.8 mmol, 90.8 %).

**$^1$H NMR (400.1 MHz, $CDCl_3$, 300 K): δ** 13.09 (s, 1H, NH, NH), 7.26 ($CDCl_3$), 7.13 (broad m, 7H, CH, $C_6H_3$), 5.50 (m, 1H, NH, C(=O)NH), 4.21 (m, 1H, CH, $^i$Pr), 3.05 (m, 4H, CH, $^i$Pr), 1.82 (s, 6H, $CH_3$, Me), 1.55 ($H_2O$), 1.24 (d, 7H, $CH_3$, $^i$Pr), 1.20 (d, 3H, $CH_3$, $^i$Pr), 1.11 (d, 12H, $CH_3$, $^i$Pr) 0.96 (hexane) ppm

**$^{13}$C{$^1$H} NMR (100.6 MHz, $CDCl_3$, 300 K): δ** 160.3 ($C_{quaternary}$, C=O), 142.3 ($C_{quaternary}$, $C_6H_3$), 139.8 ($C_{quaternary}$, $C_6H_3$), 125.5 (CH, $C_6H_3$), 123.2 (CH, $C_6H_3$), 77.2 ($CDCl_3$), 41.8 (CH, $^i$Pr), 28.2 (CH, $^i$Pr), 24.3 ($CH_3$, $^i$Pr), 23.3 ($CH_3$, $^i$Pr), 22.7 ($CH_3$, $^i$Pr), 18.3 ($CH_3$, Me) ppm

**IR spectrum:** ν 3444.7 (s, NH stretching), 3300.1 (broad s, NH stretching), 1626.7 (s, C=O stretching) $cm^{-1}$

**Melting point analysis**: 151-153 °C

Figure S8: ORTEP diagram of [(MeCNH-Dipp)(MeCN-Dipp)C($^{i}$PrNHCO)] (**8**)

Molecular structure of **8**, with selected hydrogen atoms displayed and organic groups shown as wire frame for clarity. Thermal ellipsoids are displayed at 40 % probability level.

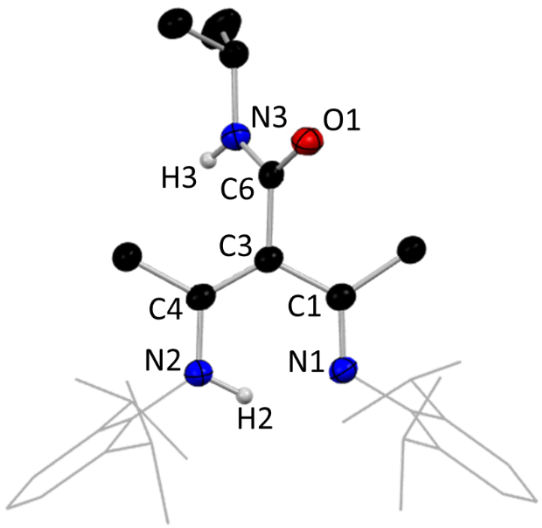


Table S11: Selected bond lengths (Å) and bond angles (°) of **8**

| **Atoms** | **Bond Lengths (Å)** |
|---|---|
| N1-C1 | 1.304(2) |
| C1-C3 | 1.456(2) |
| C3-C4 | 1.387(3) |
| C4-N2 | 1.354(2) |
| C3-C6 | 1.505(2) |
| C6-N3 | 1.345(2) |
| C6-O1 | 1.234(2) |
| N3-C7 | 1.467(2) |
| **Atoms** | **Bond Angles (°)** |
| C4-C3-C1 | 123.47(16) |
| C1-C3-C6 | 115.98(15) |
| C4-C3-C6 | 120.48(15) |
| C3-C6-O1 | 121.97(16) |
| O1-C6-N3 | 120.55(16) |
| C3-C6-N3 | 117.39(15) |
| C6-N3-C7 | 122.24(17) |

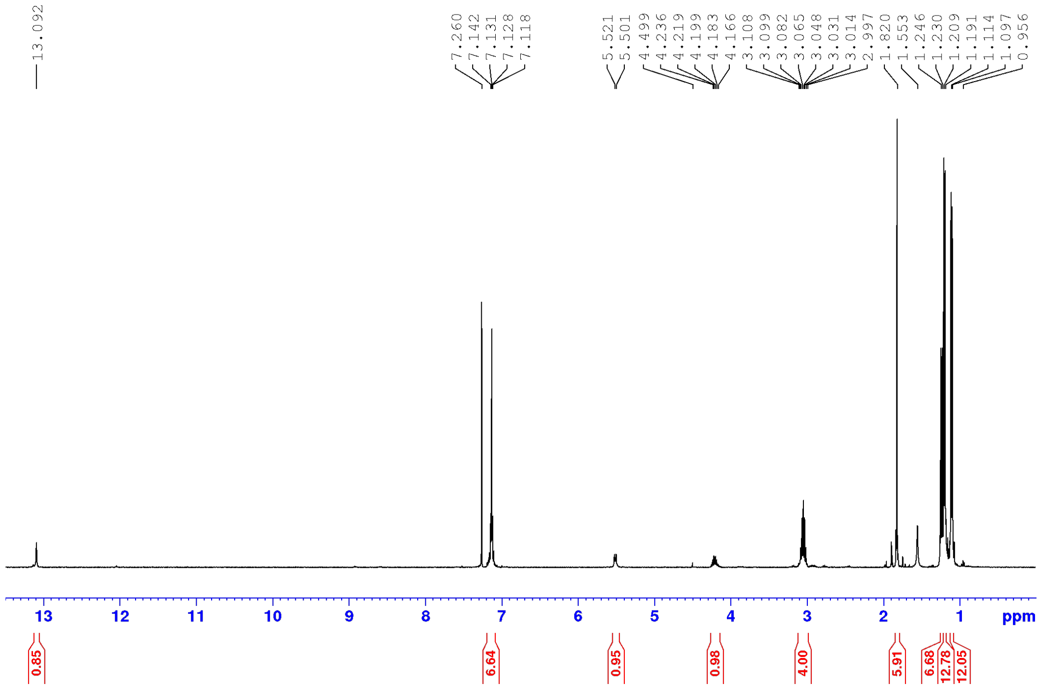


**Figure S8.1**: $^{1}H$ NMR spectrum in $CDCl_3$ of **8** [(MeCNH-Dipp)(MeCN-Dipp)C($^{i}$PrNHCO)]

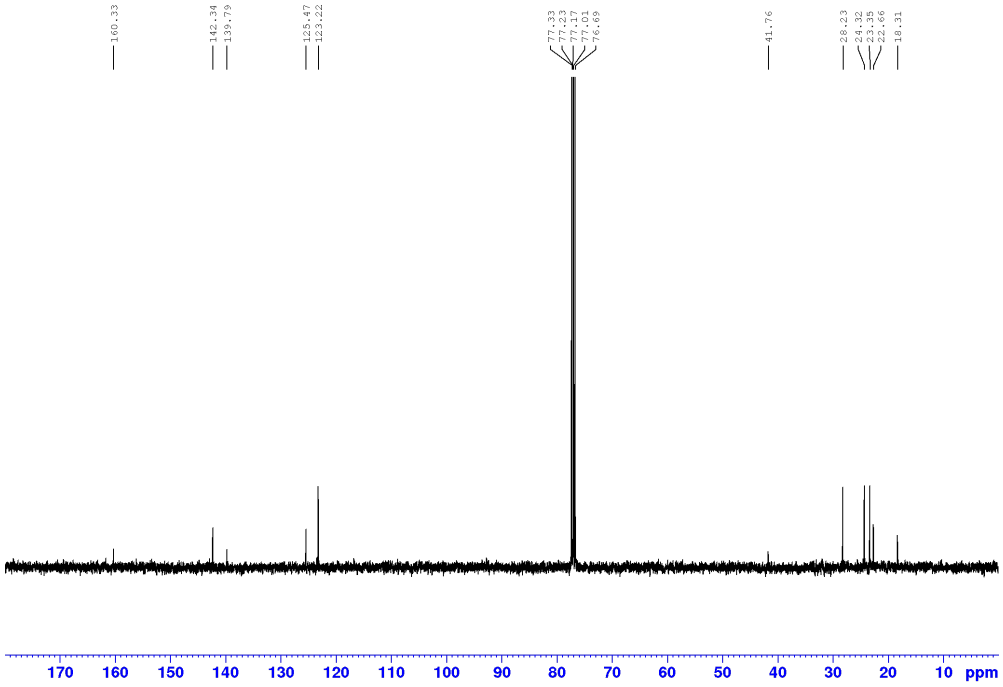


**Figure S8.2**: $^{13}C\{^{1}H\}$ NMR spectrum in $CDCl_3$ of **8** [(MeCNH-Dipp)(MeCN-Dipp)C($^{i}$PrNHCO)]

## Synthesis of [{(MeCN-Dipp)$_2$C($^i$PrNHCO)}Li·THF]$_2$ (**8a**)

NacNacLi (0.213 g, 0.5 mmol) placed in vial, dissolved in 5 ml hexane, $^i$PrNCO (49 µl, 0.5 mmol) added to form a white suspension. THF (0.5 ml) added, and crop of colourless crystals grew upon slow evaporation of solvent (0.16 g, 0.3 mmol, 64.0 %).

**$^1$H NMR (400.1 MHz, $C_6D_6$, 300 K): δ** 11.64 (m, 1H, NH, NH), 7.24 (m, 2H, CH, $C_6H_3$), 7.16 ($C_6D_6$), 7.12 (m, 2H, CH, $C_6H_3$), 7.05 (m, 1H, CH, $C_6H_3$), 3.66 (br m, 6H, $CH_2$, THF), 3.48 (m, 1H, CH, NH$^i$Pr), 3.22 (m, 4H, CH, $^i$Pr), 1.96 (s, 3H, $CH_3$, Me), 1.93 (s, 3H, $CH_3$, Me), 1.48 (br m, 6H, $CH_2$, THF), 1.30-1.16 (broad m, 28H, $CH_3$, $^i$Pr), 0.89 (hexane), 0.81 (d, 6H, $CH_3$, NH$^i$Pr) ppm

**$^{13}$C{$^1$H} NMR (100.6 MHz, $C_6D_6$, 300 K): δ** 172.0 ($C_{quaternary}$, C=O), 168.8 ($C_{quaternary}$, CMe), 167.5 ($C_{quaternary}$, CMe), 147.7 ($C_{quaternary}$, $C_6H_3$), 147.4 ($C_{quaternary}$, $C_6H_3$), 138.9 ($C_{quaternary}$, $C_6H_3$), 138.1 ($C_{quaternary}$, $C_6H_3$), 128.0 ($C_6D_6$), 124.1 (CH, $C_6H_3$), 124.0 (CH, $C_6H_3$), 123.6 (CH, $C_6H_3$), 123.3 (CH, $C_6H_3$), 98.1 ($C_{quaternary}$, γ-C), 68.0 ($CH_2$, THF), 40.7 (CH, NH$^i$Pr), 28.2 (CH, $^i$Pr), 26.8 ($CH_3$, Me), 25.7 ($CH_2$, THF), 25.0 ($CH_3$, Me), 24.3 ($CH_3$, $^i$Pr), 24.0 ($CH_3$, $^i$Pr), 23.7 ($CH_3$, NH$^i$Pr), 14.3 ($CH_3$, Me) ppm

**$^7$Li NMR (155.5 MHz, $C_6D_6$, 298K) δ** 1.65 ppm

**IR spectrum:** ν 1593.4 (s, C=O stretching) $cm^{-1}$

**Melting point analysis**: 148-150 °C

Figure S8a: ORTEP diagram of [{(MeCN-Dipp)$_2$C($^i$PrNHCO)}Li.THF]$_2$ (**8a**)

Molecular structure of **8a**, with selected hydrogen atom displayed and organic groups shown as wire frame for clarity. Thermal ellipsoids are displayed at 40 % probability level, second molecule not included.

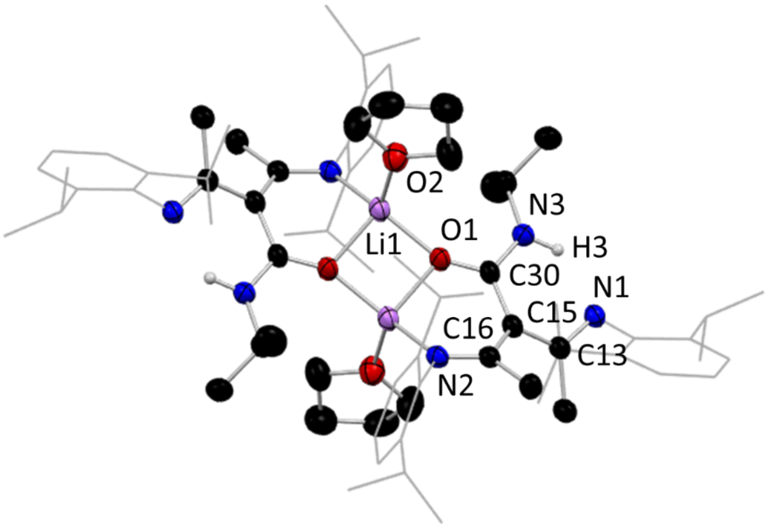


Note that compound **8a** has previously been published by the Mulvey group with structural data presented in the CSD (deposition number 1947187).[6]

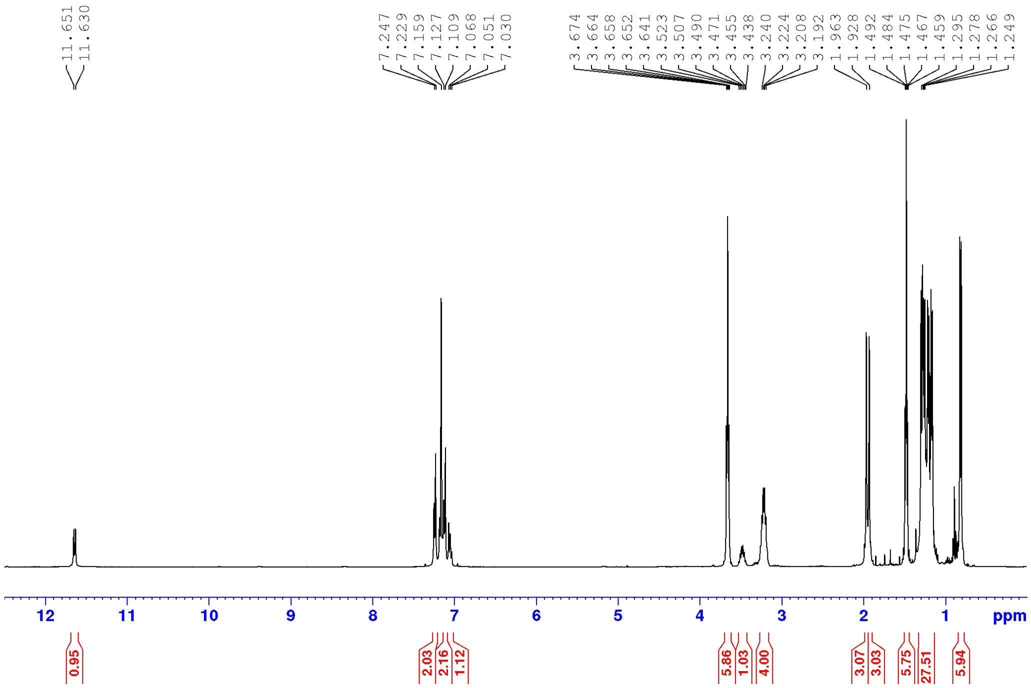


**Figure S8a.1**: $^{1}$H NMR spectrum in $C_6D_6$ of **8a** [{(MeCN-Dipp)$_2$C($^{i}$PrNHCO)}Li·THF]$_2$

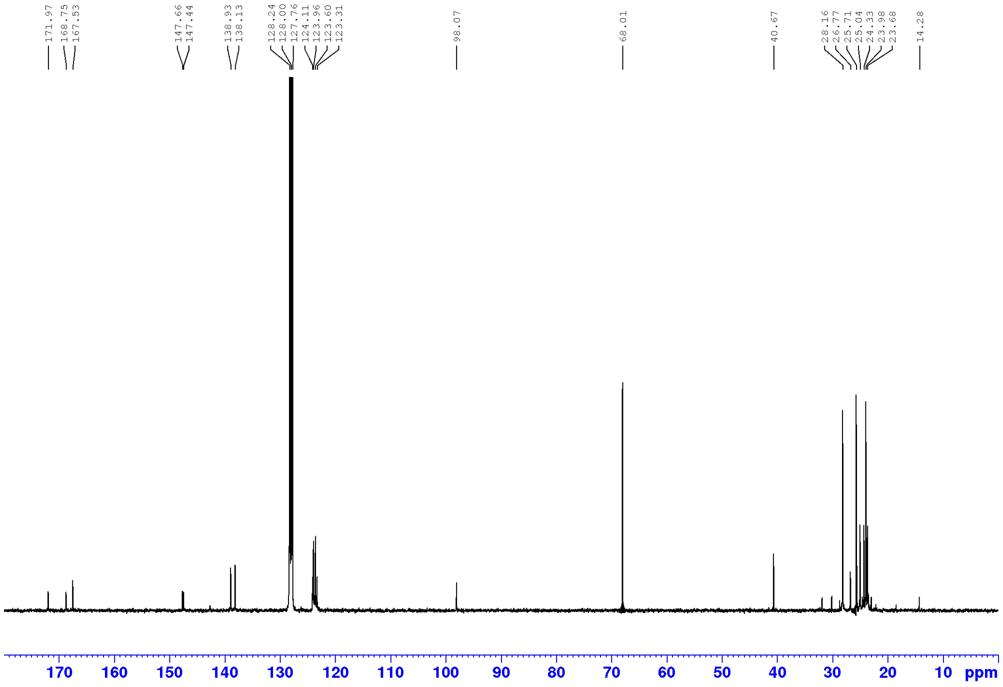


**Figure S8a.2**: $^{13}$C{$^{1}$H} NMR spectrum in $C_6D_6$ of **8a** [{(MeCN-Dipp)$_2$C($^{i}$PrNHCO)}Li·THF]$_2$

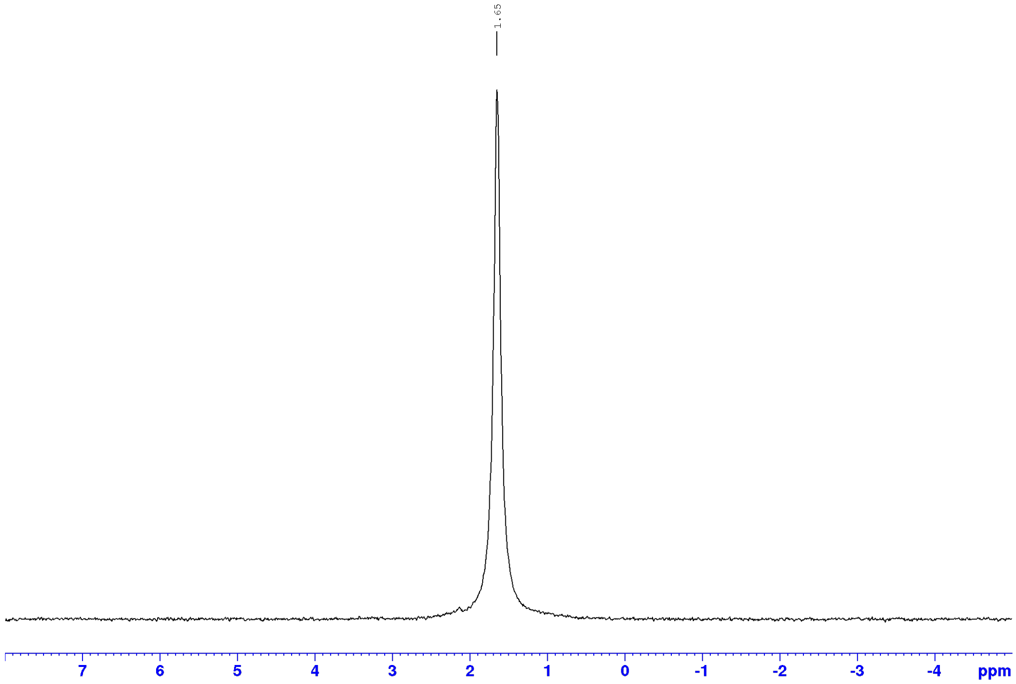


**Figure S8a.3**: $^{7}Li$ NMR spectrum in $C_6D_6$ of **8a** [{(MeCN-Dipp)$_2$C($^{i}$PrNHCO)}Li·THF]$_2$

## Synthesis of [(MeCNH-Dipp)(MeCN-Dipp)C(EtNHCO)] (**9**)

NacNacLi (0.85 g, 2.0 mmol) dissolved in hexane (10 ml) to produce a yellow solution before EtNCO (0.16 ml, 2.0 mmol) was added giving an off-white suspension. Suspension stirred (3 hrs) before deionised water (5 ml) was added and mixture exposed to air and stirred overnight. Separation performed using DCM (15 ml), organic layer dried using $MgSO_4$ before product was filtered, dried, and crystallised from methanol. Product was collected as off-white/yellow crystals (0.70 g, 1.42 mmol, 71 %).

**$^1$H NMR (400.1 MHz, $CDCl_3$, 300 K): δ** 13.12 (s, 1H, NH, NH), 7.26 ($CDCl_3$), 7.13 (broad m, 7H, CH, $C_6H_3$), 5.72 (s, 1H, NH, C(=O)NH), 3.43 (m, 2H, $CH_2$, Et), 3.05 (sep, 4H, CH, $^i$Pr), 1.83 (s, 6H, $CH_3$, Me), 1.61 ($H_2O$), 1.25-1.23 (m, 3H, $CH_3$, Et), 1.20 (d, 15H, $CH_3$, $^i$Pr), 1.11 (d, 12H, $CH_3$, $^i$Pr) ppm

**$^{13}$C{$^1$H} NMR (100.6 MHz, $CDCl_3$, 300 K): δ** 171.4 ($C_{quaternary}$, C=O), 160.5 ($C_{quaternary}$, CMe), 142.5 ($C_{quaternary}$, $C_6H_3$), 139.9 ($C_{quaternary}$, $C_6H_3$), 125.6 (CH, $C_6H_3$), 123.4 (CH, $C_6H_3$), 105.3 ($C_{quaternary}$, γ-C), 77.16 ($CDCl_3$), 35.0 ($CH_2$, Et), 28.4 (CH, $^i$Pr), 24.4 ($CH_3$, $^i$Pr), 23.5 ($CH_3$, $^i$Pr), 18.5 ($CH_3$, Me), 15.0 ($CH_3$, Et) ppm

**IR spectrum:** ν 3331.5 (broad m, NH stretching, -NH), 1700.4 (s, C=O stretching) $cm^{-1}$

**Melting point analysis**: 125-127 °C

Figure S9: ORTEP diagram of [(MeCNH-Dipp)(MeCN-Dipp)C(EtNHCO)] (**9**)

Molecular structure of **9**, with selected hydrogen atoms displayed with organic groups shown as wire frame for clarity. Thermal ellipsoids are displayed at 40 % probability level.

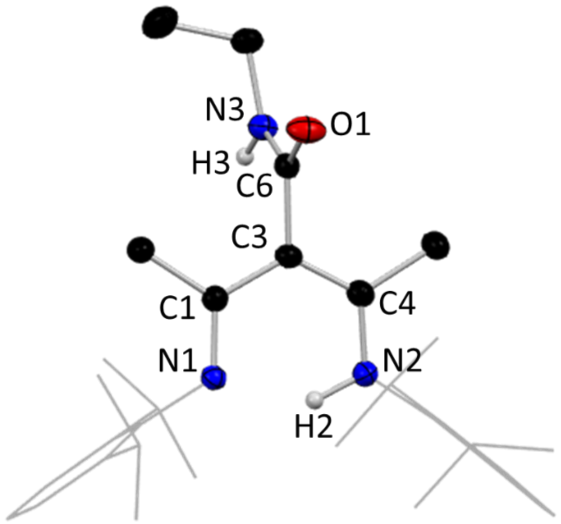


Table S12: Selected bond lengths (Å) and bond angles (°) of **9**

| Atoms | Bond Lengths (Å) |
|---|---|
| N1-C1 | 1.3143(14) |
| C1-C3 | 1.4397(14) |
| C3-C4 | 1.3916(15) |
| C4-N2 | 1.3424(14) |
| C3-C6 | 1.5086(14) |
| C6-N3 | 1.3461(14) |
| C6-O1 | 1.2341(13) |
| N3-C7 | 1.4587(14) |
| **Atoms** | **Bond Angles (°)** |
| C4-C3-C1 | 123.92(10) |
| C1-C3-C6 | 117.38(9) |
| C4-C3-C6 | 118.70(9) |
| C3-C6-O1 | 122.72(10) |
| O1-C6-N3 | 122.45(10) |
| C3-C6-N3 | 114.83(9) |
| C6-N3-C7 | 122.37(10) |

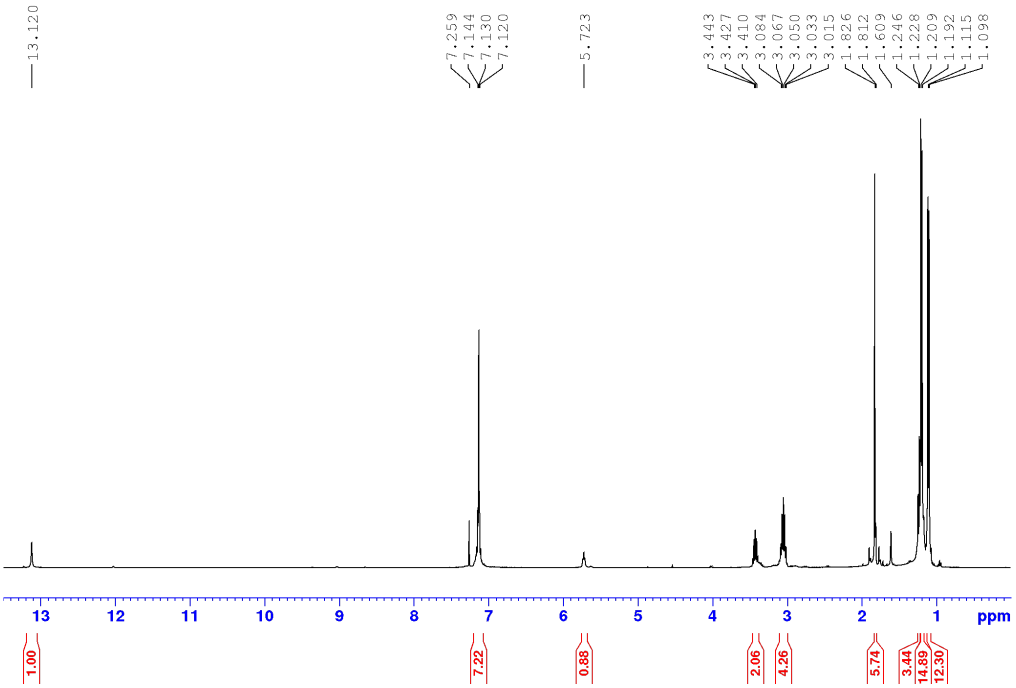


**Figure S9.1**: $^{1}$H NMR spectrum in $CDCl_3$ of **9** [(MeCNH-Dipp)(MeCN-Dipp)C(EtNHCO)]

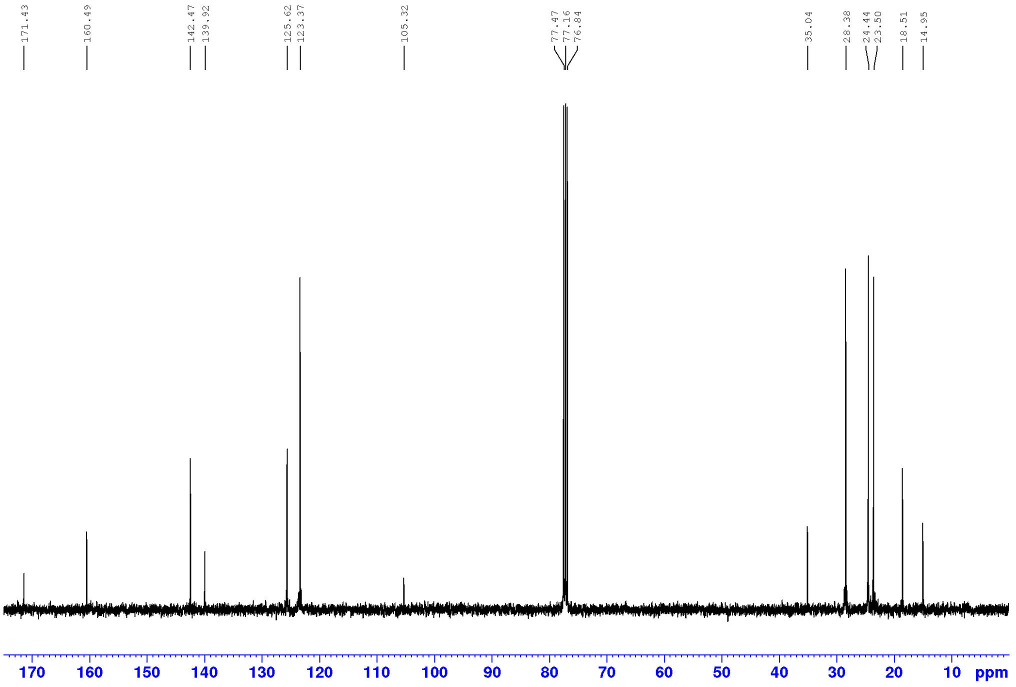


**Figure S9.2**: $^{13}C\{^1H\}$ NMR spectrum in $CDCl_3$ of **9** [(MeCNH-Dipp)(MeCN-Dipp)C(EtNHCO)]

## Synthesis of [{(MeCN-Dipp)$_2$C(EtNHCO)}Li·THF]$_2$ (**9a**)

NacNacLi (0.213 g, 0.5 mmol) dissolved in hexane (2 ml) to produce a yellow solution before EtNCO (40 µl, 0.5 mmol) was added giving an off-white suspension. Suspension stirred overnight before THF (0.5 ml) was added, suspension left to settle before solvent layer was filtered and placed in a -20°C freezer. Product was collected as off-white/yellow crystals (0.11 g, 0.17 mmol, 34.7 %).

**$^1$H NMR (400.1 MHz, $C_6D_6$, 300 K): δ** 11.56 (s, 1H, NH, C(=O)NH), 7.24 (m, 2H, CH, $C_6H_3$), 7.16 ($C_6D_6$), 7.11 (m, 2H, CH, $C_6H_3$), 7.04 (broad m, 1H, CH, $C_6H_3$), 3.62 (m, 8H, $CH_2$, THF), 3.19 (sep, 4H, CH, $^i$Pr), 2.69 (s, 2H, $CH_2$, Et), 1.98 (s, 3H, $CH_3$, Me), 1.94 (s, 3H, $CH_3$, Me), 1.45 (m, 8H, $CH_2$, THF), 1.30 (d, 7H, $CH_3$, $^i$Pr), 1.21 (dd, 12H, $CH_3$, $^i$Pr), 1.17 (s, 6H, $CH_3$, $^i$Pr), 0.90 (hexane), 0.76 (m, 3H, $CH_3$, Et) ppm

**$^{13}$C{$^1$H} NMR (100.6 MHz, $C_6D_6$, 300 K): δ** 171.7 ($C_{quaternary}$, C=O), 168.3 ($C_{quaternary}$, CMe), 147.8 ($C_{quaternary}$, $C_6H_3$), 147.0 ($C_{quaternary}$, $C_6H_3$), 138.9 ($C_{quaternary}$, $C_6H_3$), 138.0 ($C_{quaternary}$, $C_6H_3$), 128.0 ($C_6D_6$), 124.1 (CH, $C_6H_3$), 123.9 (CH, $C_6H_3$), 123.5 (CH, $C_6H_3$), 123.3 (CH, $C_6H_3$), 98.1 ($C_{quaternary}$, γ-C), 67.9 ($CH_2$, THF), 34.3 ($CH_2$, Et), 28.3 (CH, $^i$Pr), 28.1 (CH, $^i$Pr), 26.5 ($CH_3$, Me), 25.7 ($CH_2$, THF), 25.2 ($CH_3$, Me), 24.7 ($CH_3$, $^i$Pr), 24.3 ($CH_3$, $^i$Pr), 23.9 ($CH_3$, $^i$Pr), 23.4 ($CH_3$, $^i$Pr), 14.7 ($CH_3$, Et) ppm

**$^7$Li NMR (155.5 MHz, $C_6D_6$, 298K) δ** 1.66 ppm

**IR spectrum:** ν 1672.0 (s, C=O stretching) $cm^{-1}$

**Melting point analysis**: 178-180 °C

Figure S9a: ORTEP diagram of [{(MeCN-Dipp)$_2$C(EtNHCO)}Li·THF]$_2$ (**9a**)

Molecular structure of **9a**, with selected hydrogen atom displayed and organic groups shown as wire frame for clarity. Thermal ellipsoids are displayed at 40 % probability level. Co-crystallised solvent molecule omitted.

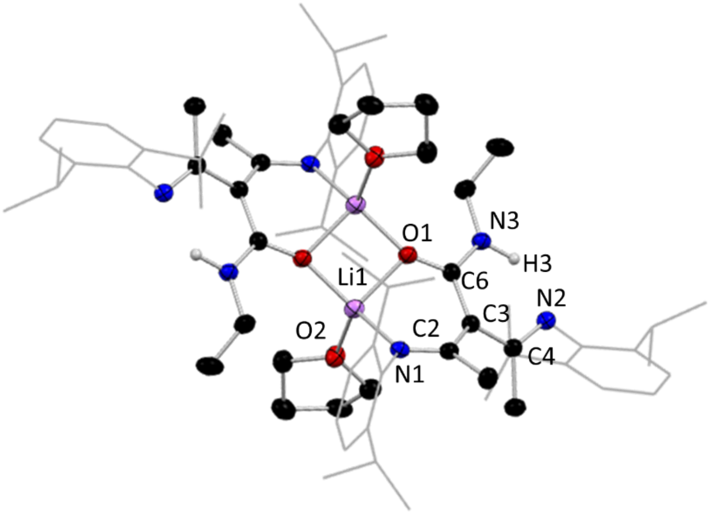

Table S13: Selected bond lengths (Å) and bond angles (°) of **9a**

| Atoms | Bond Lengths (Å) |
|---|---|
| Li1-O1 | 1.936(3) |
| Li1-O1’ | 1.870(3) |
| Li1’-O1 | 1.870(3) |
| Li1-O2 | 1.984(3) |
| Li1-N1 | 2.017(3) |
| N1-C2 | 1.298(2) |
| C2-C3 | 1.468(2) |
| C3-C4 | 1.439(2) |
| C4-N2 | 1.311(2) |
| C3-C6 | 1.443(2) |
| C6-N3 | 1.351(2) |
| C6-O1 | 1.273(2) |
| N3-C7 | 1.455(3) |
| **Atoms** | **Bond Angles (°)** |
| O1-Li1-O1’ | 91.52(15) |
| N1-Li1-O1 | 90.27(14) |
| N1-Li1-O2 | 107.36(16) |
| O1-Li1-O2 | 123.71(17) |
| C4-C3-C2 | 118.87(15) |
| C2-C3-C6 | 119.41(15) |
| C4-C3-C6 | 121.59(15) |
| C3-C6-O1 | 122.49(15) |
| O1-C6-N3 | 118.72(16) |
| C3-C6-N3 | 118.78(16) |
| C6-N3-C7 | 123.49(16) |

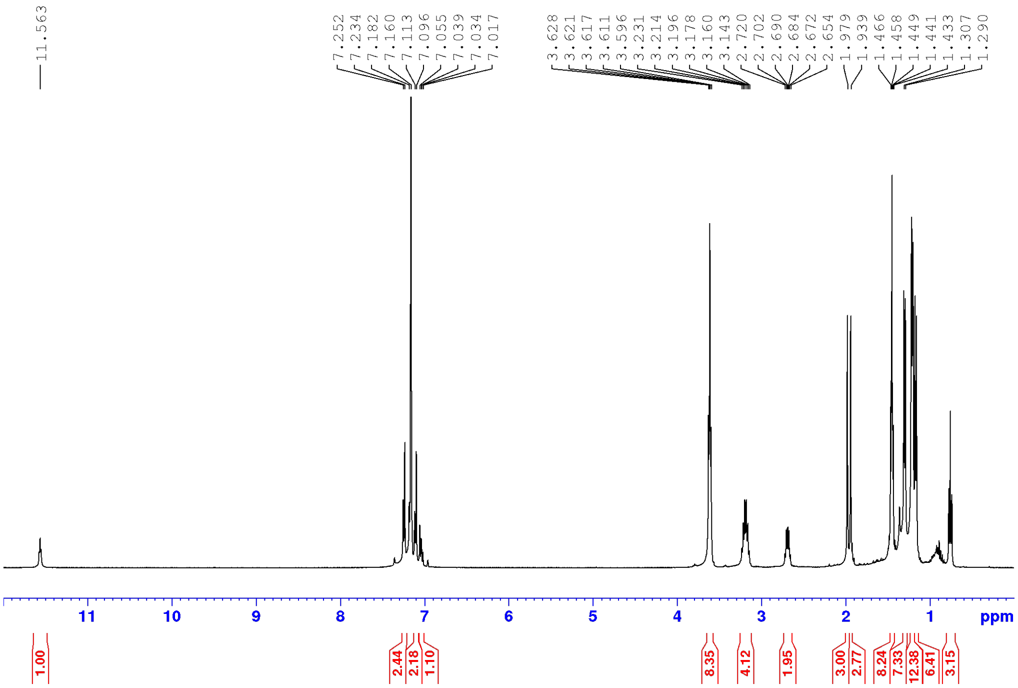


**Figure S9a.1**: $^{1}H$ NMR spectrum in $C_6D_6$ of **9a** [{(MeCN-Dipp)$_2$C(EtNHCO)}Li·THF]$_2$

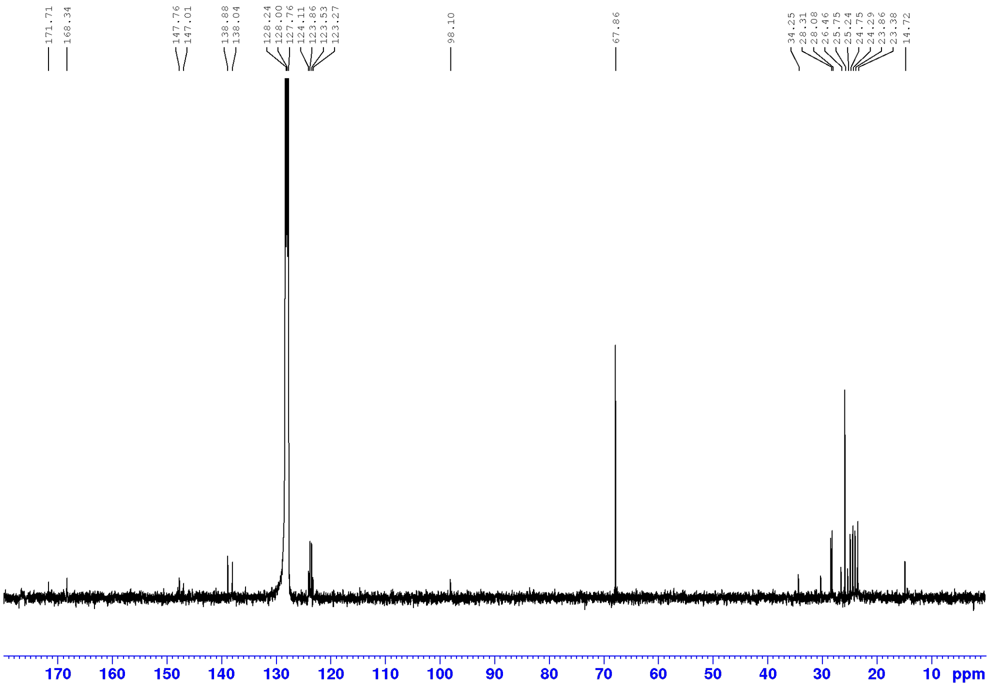


**Figure S9a.2**: $^{13}C\{^{1}H\}$ NMR spectrum in $C_6D_6$ of **9a** [{(MeCN-Dipp)$_2$C(EtNHCO)}Li·THF]$_2$

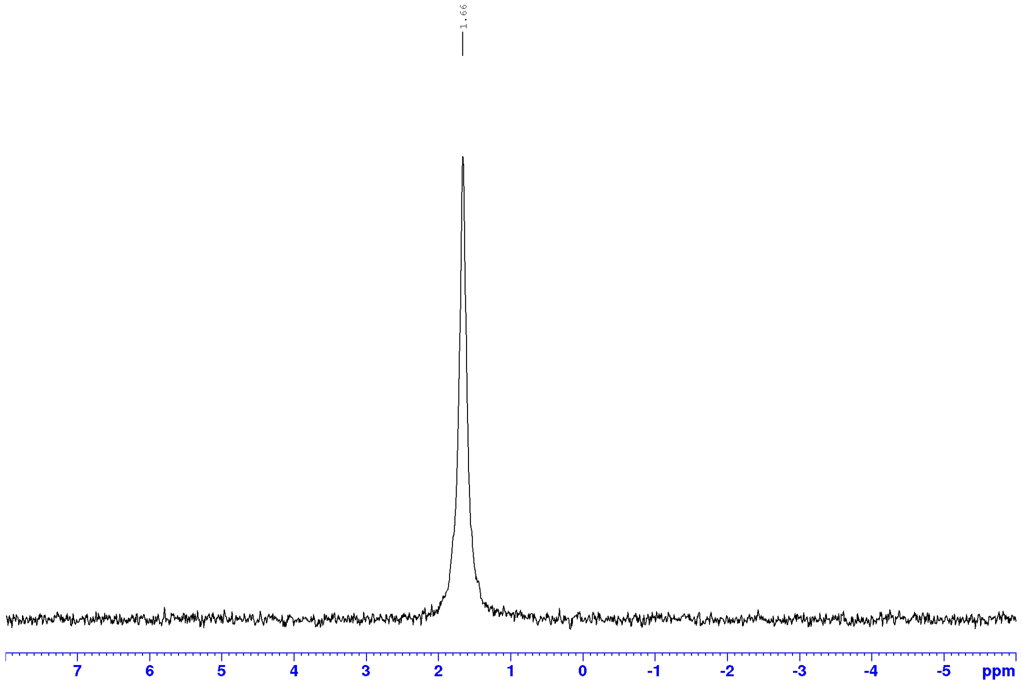


**Figure S9a.3**: $^{7}$Li NMR spectrum in $C_6D_6$ of **9a** [{(MeCN-Dipp)$_2$C(EtNHCO)}Li·THF]$_2$

## Synthesis of [(MeCNH-Dipp)(MeCN-Dipp)C{C(O)N($C_6H_4$OMe)C(O)N(H)$C_6H_4$OMe}] (**10**)

NacNacLi (0.859 g, 2.0 mmol) dissolved in hexane (10 ml), 4-OMePhNCO (0.26 ml, 2.0 mmol) added to form a yellow suspension. Suspension stirred (3 hrs) before deionised water (5 ml) was added, reaction exposed to air and stirred overnight. Separation performed using DCM (15 ml), organic layer concentrated, and products crystallised. Due to the mixed nature of the crystalline product, an individual yield for compound **10** could not be obtained, thus a crude yield of the mixture of compound 2 and 10 is provided (0.930 g) . Crystals of compound **10** exist as large colourless blocks that are visually distinct under a microscope from neighbouring crystals of compound **2**.

**$^1$H NMR (400.1 MHz, $CDCl_3$, 300 K): δ** 13.14 (s, 1H, NH, NH), 11.78 (s, 1H, NH, C(=O)NH), 7.66 (m, 2H, CH, $C_6H_3$), 7.39 (m, 2H, CH, $C_6H_3$), 7.32 (m, 3H, CH, $C_6H_3$), 7.26 ($CDCl_3$), 7.10 (m, 3H, CH, $C_6H_4$), 7.00 (m, 2H, CH, $C_6H_4$), 4.02 (s, 3H, $CH_3$, $C_6H_4$Me), 3.93 (s, 3H, $CH_3$, $C_6H_4$Me), 3.13 (m, 2H, CH, $^i$Pr), 2.66 (m, 2H, CH, $^i$Pr), 1.98 (s, 6H, $CH_3$, Me), 1.69 ($H_2O$), 1.34 (d, 13H, $CH_3$, $^i$Pr), 1.24 (d, 7H, $CH_3$, $^i$Pr), 1.07 (d, 6H, $CH_3$, $^i$Pr) ppm

**$^{13}$C{$^1$H} NMR (100.6 MHz, $CDCl_3$, 300 K): δ** 177.0 ($C_{quaternary}$, C=O), 159.1 ($C_{quaternary}$, CMe), 156.3 ($C_{quaternary}$, COMe), 152.9 ($C_{quaternary}$, COMe), 142.2 ($C_{quaternary}$, $^i$Pr), 142.2 ($C_{quaternary}$, $^i$Pr), 139.2 ($C_{quaternary}$, $C_6H_3$), 131.9 ($C_{quaternary}$, $C_6H_3$), 131.2 ($C_{quaternary}$, $C_6H_3$), 129.7 (CH, $C_6H_3$), 125.9 (CH, $C_6H_3$), 123.6 (CH, $C_6H_3$), 123.4 (CH, $C_6H_3$), 121.6 (CH, $C_6H_4$), 114.3 (CH, $C_6H_4$), 114.0 (CH, $C_6H_4$), 105.6 ($C_{quaternary}$, γ-C), 77.2 ($CDCl_3$), 55.6 ($CH_3$, OMe), 55.5 ($CH_3$, OMe), 28.3 (CH, $^i$Pr), 27.9 (CH, $^i$Pr), 24.6 ($CH_3$, $^i$Pr), 24.4 ($CH_3$, $^i$Pr), 23.9 ($CH_3$, $^i$Pr), 23.2 ($CH_3$, $^i$Pr), 19.5 ($CH_3$, Me) ppm

**IR spectrum:** ν 1716.8 (s, C=O stretching) $cm^{-1}$

**Melting point analysis**: 173-175 °C

Figure S10: ORTEP diagram of [(MeCNH-Dipp)(MeCN-Dipp)C{C(O)N($C_6H_4$OMe)C(O)N(H)$C_6H_4$OMe}] (**10**)

Molecular structure of **10**, with selected hydrogen atoms displayed with organic groups shown as wire frame for clarity. Thermal ellipsoids are displayed at 40 % probability level.

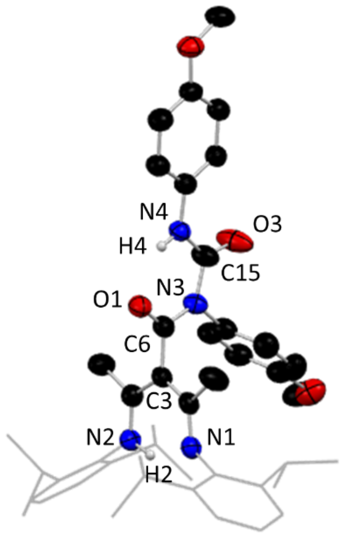

Table S14: Selected bond lengths (Å) and bond angles (°) of **10**

| Atoms | Bond Lengths (Å) |
|---|---|
| N1-C1 | 1.310(3) |
| C1-C3 | 1.421(3) |
| C3-C4 | 1.408(3) |
| C4-N2 | 1.325(3) |
| C3-C6 | 1.496(3) |
| C6-O1 | 1.223(3) |
| C6-N3 | 1.398(3) |
| N3-C8 | 1.462(3) |
| C11-O2 | 1.371(3) |
| O2-C14 | 1.424(3) |
| N3-C15 | 1.437(3) |
| C15-O3 | 1.218(3) |
| C15-N4 | 1.346(3) |
| C19-O4 | 1.376(2) |
| O4-C22 | 1.421(3) |
| **Atoms** | **Bond Angles (°)** |
| C1-C3-C4 | 124.30(18) |
| C4-C3-C6 | 119.00(18) |
| C1-C3-C6 | 116.56(16) |
| C3-C6-N3 | 115.74(18) |
| C3-C6-O1 | 122.50(19) |
| N3-C6-O1 | 121.74(19) |
| C6-N3-C8 | 120.70(17) |
| N3-C15-O3 | 119.0(2) |
| N3-C15-N4 | 115.70(19) |
| C15-N4-C16 | 125.97(19) |

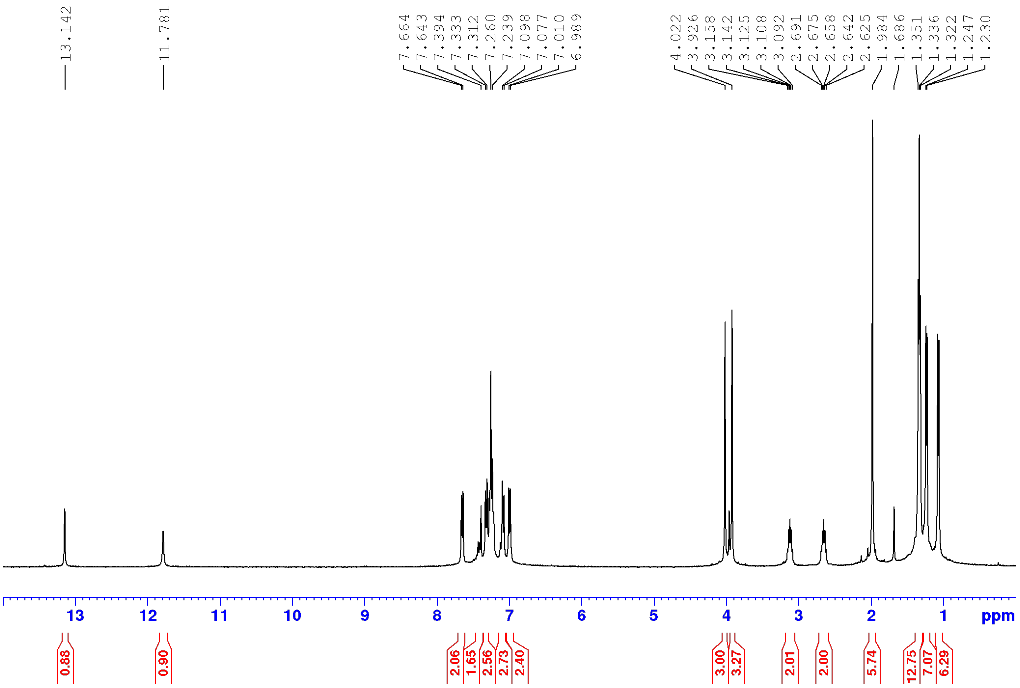


**Figure S10.1**: $^{1}H$ NMR spectrum in $CDCl_3$ of **10** [(MeCNH-Dipp)(MeCN-Dipp)C{C(O)N($C_6H_4$OMe)C(O)N(H)$C_6H_4$OMe}]

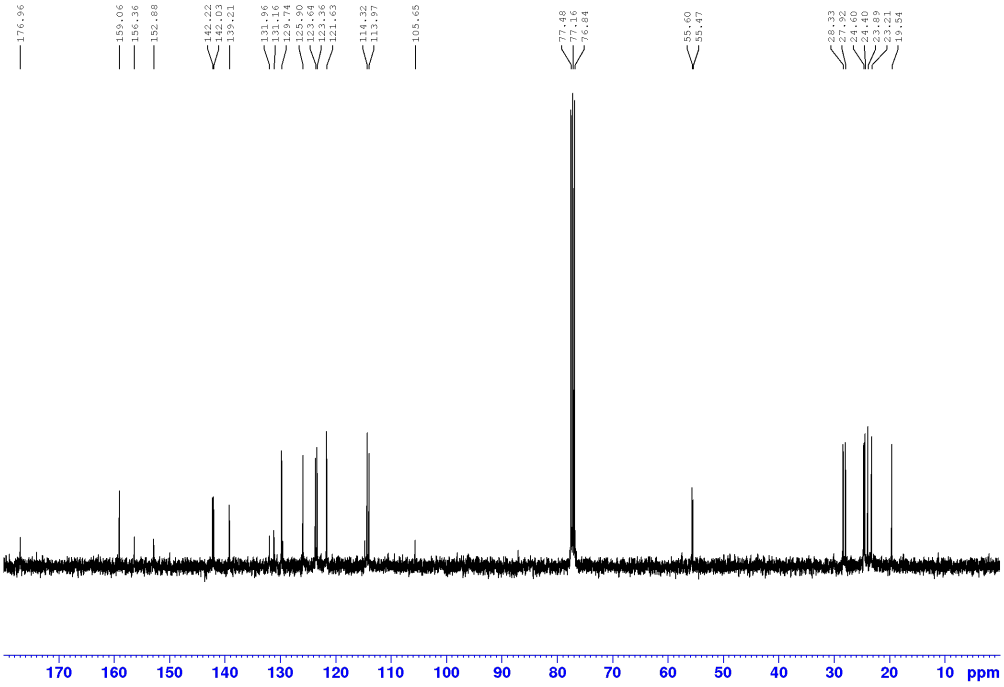


**Figure S10.2**: $^{13}C\{^{1}H\}$ NMR spectrum in $CDCl_3$ of **10** [(MeCNH-Dipp)(MeCN-Dipp)C{C(O)N($C_6H_4$OMe)C(O)N(H)$C_6H_4$OMe}]

## Synthesis of [(MeCNH-Dipp)(MeCN-Dipp)C(4-Me$C_6H_4$NHCS)] (**11**)

NacNacH (0.87 g, 2.1 mmol) dissolved in hexane (10 ml), $^{n}$BuLi (2.0 ml, 1.2 M, 2.4 mmol) added at 0°C and stirred overnight to produce a yellow solution. Yellow suspension obtained on addition of 4-MePhNCS (0.30 ml, 2.0 mmol), suspension refluxed (3 hrs) before deionised water (5 ml) was added and mixture exposed to air before being stirred overnight. Separation performed using DCM (15 ml), organic layer dried using $MgSO_4$ before product was filtered, dried, and crystallised from methanol. Product was collected as yellow crystals (0.66 g, 1.04 mmol, 58.0 %).

**$^{1}$H NMR (400.1 MHz, $CDCl_3$, 300 K): δ** 13.54 (s, 1H, NH, NH), 9.71 (s, 1H, NH, C(=S)NH), 7.26 ($CDCl_3$), 7.17 (broad m, 11H, CH, $C_6H_3$), 7.07 (broad m, 3H, CH, $C_6H_4$), 3.08 (sep, 4H, CH, $^{i}$Pr), 2.74 (sep, 2H, CH, $^{i}$Pr), 2.40 (s, 3H, $CH_3$, $C_6H_3$Me), 1.87 (s, 6H, $CH_3$, Me), 1.56 ($H_2O$), 1.20 (d, 8H, $CH_3$, $^{i}$Pr), 1.11 (d, 9H, $CH_3$, $^{i}$Pr), 1.06 (d, 7H, $CH_3$, $^{i}$Pr), 1.00 (d, 6H, $CH_3$, $^{i}$Pr) ppm

Residual NacNacH: 7.63 (CH, $C_6H_3$), 7.49 (CH, $C_6H_3$), 1.97 ($CH_3$, Me), 1.91 ($CH_3$, Me), 1.24-1.22 ($CH_3$, $^{i}$Pr), 1.15-1.13 ($CH_3$, $^{i}$Pr) ppm

**$^{13}$C{$^{1}$H} NMR (100.6 MHz, $CDCl_3$, 300 K): δ** 205.3 ($C_{quaternary}$, C=S), 159.6 ($C_{quaternary}$, CMe), 142.5 ($C_{quaternary}$, $C_6H_3$), 141.7 ($C_{quaternary}$, $C_6H_3$), 139.5 ($C_{quaternary}$, $C_6H_3$), 136.7 ($C_{quaternary}$, $C_6H_3$), 135.9 ($C_{quaternary}$, $C_6H_3$), 129.7 (CH, $C_6H_3$), 125.5 (CH, $C_6H_3$), 123.4 (CH, $C_6H_4$), 123.3 (CH, $C_6H_4$), 123.1 (CH, $C_6H_4$), 122.2 (CH, $C_6H_4$), 107.3 ($C_{quaternary}$, γ-C), 77.1 ($CDCl_3$), 28.3 (CH, $^{i}$Pr), 28.1 (CH, $^{i}$Pr, 24.3 ($CH_3$, $^{i}$Pr), 23.5 ($CH_3$, $^{i}$Pr), 23.4 ($CH_3$, $^{i}$Pr), 23.1 ($CH_3$, $^{i}$Pr), 21.1 ($CH_3$, $C_6H_4$Me), 19.1 ($CH_3$, Me) ppm

**IR spectrum:** ν 3167.9 (s, NH stretching), 3159.8 (s, NH stretching) $cm^{-1}$

**Melting point analysis**: 138-140 °C

Figure S11: ORTEP diagram of [(MeCNH-Dipp)(MeCN-Dipp)C(4-MeC$_6$H$_4$NHCS)] (**11**)

Molecular structure of **11**, with selected hydrogen atoms displayed with organic groups shown as wire frame for clarity. Thermal ellipsoids are displayed at 40 % probability level.

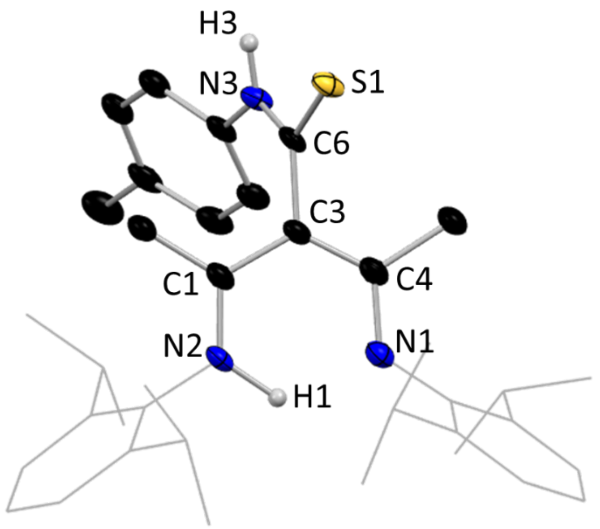


The X-ray crystallographic data of compound **11** are of insufficient quality to allow meaningful discussion of bond lengths or bond angles.

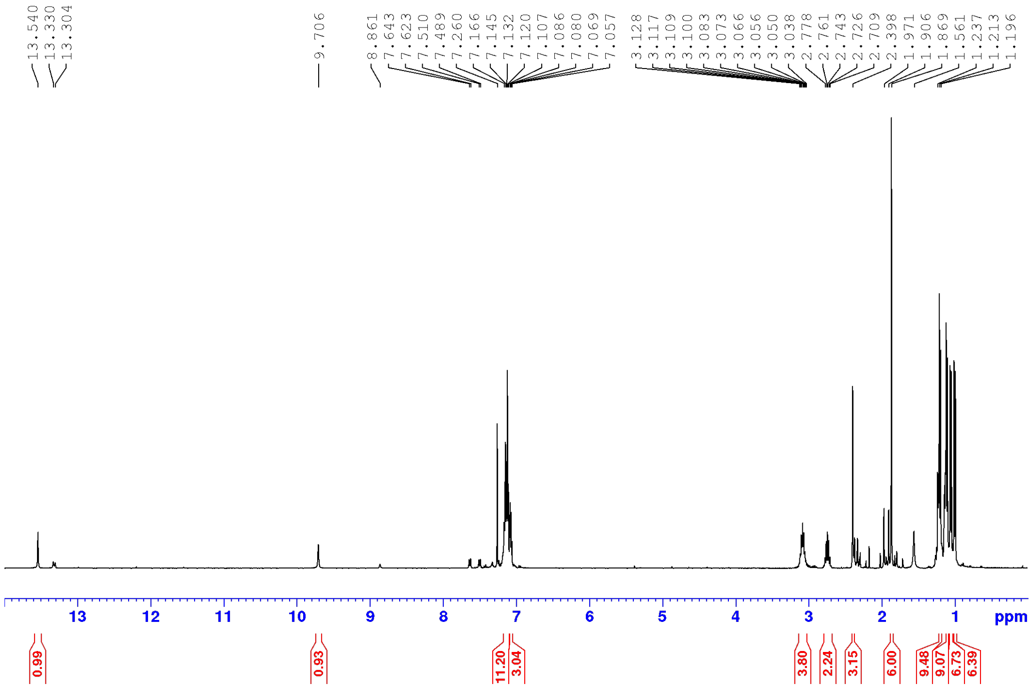


**Figure S11.1**: $^{1}$H NMR spectrum in $CDCl_3$of **11** [(MeCNH-Dipp)(MeCN-Dipp)C(4-$MeC_6H_4NHCS$)]

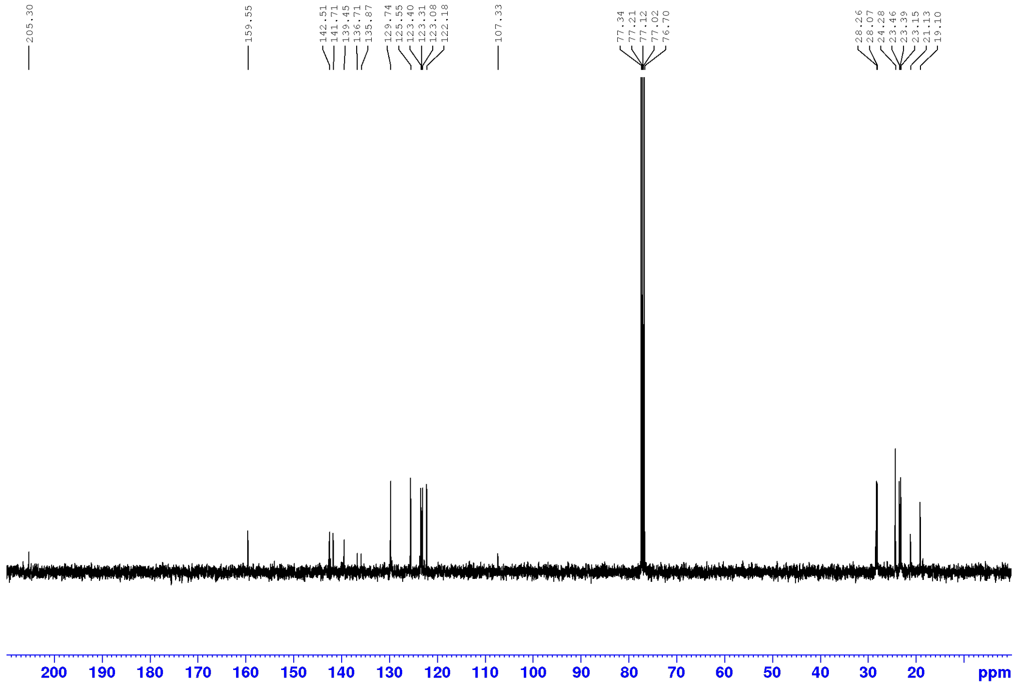


**Figure S11.2**: $^{13}C\{^{1}H\}$ NMR spectrum in $CDCl_3$ of **11** [(MeCNH-Dipp)(MeCN-Dipp)C(4-$MeC_6H_4NHCS$)]

## Synthesis of [{(MeCN-Dipp)$_2$CH(4-MeC$_6$H$_4$NHCS)}Li·THF] (**11a**)

NacNacLi (0.428 g, 1.0 mmol) dissolved in THF (5 ml) before p-tolylNCS (0.150 g, 1.0 mmol) added. Yellow solution stirred for 2 hours before being placed in -18 °C freezer. Crop of crystals formed overnight (0.493 g, 0.75 mmol, 75.2 %).

**$^1$H NMR (400.1 MHz, $C_6D_6$, 300 K): δ** 8.00 (broad s, 2H, CH, $C_6H_4$), 7.20 (broad m, 2H, CH, $C_6H_4$), 7.16 ($C_6D_6$), 7.05 (broad m, 7H, CH, $C_6H_3$), 6.11 (s, 1H, CH, γ-CH), 3.37 (broad s, 8H, $CH_2$, THF), 3.31 (broad m, 2H, CH, $^i$Pr), 2.84 (m, 2H, CH, $^i$Pr), 2.24 (s, 3H, $CH_3$, $C_6H_4$), 1.94 (s, 6H, $CH_3$, Me), 1.67 (unknown impurity), 1.18 (broad m, 22H, $CH_3$, $^i$Pr / $CH_2$, THF), 1.04 (pentane), 1.00 (d, 7H, $CH_3$, $^i$Pr) ppm

Residual NacNacH: 12.47 (NH), 4.89 (γ-CH), 1.84 ($CH_3$, Me) ppm

**$^{13}$C{$^1$H} NMR (100.6 MHz, $C_6D_6$, 300 K): δ** 173.7 ($C_{quaternary}$, C=S), 144.9 ($C_{quaternary}$, CMe), 140.1 ($C_{quaternary}$, $C_6H_3$), 137.6 ($C_{quaternary}$, $C_6H_4$), 131.4 ($C_{quaternary}$, $C_6H_4$), 128.8 (CH, $C_6H_4$), 128.2-127.8 ($C_6D_6$), 125.6 (CH, $C_6H_3$), 124.6 (CH, $C_6H_3$), 123.9 (CH, $C_6H_3$), 123.5 (CH, $C_6H_4$), 123.2 (CH, $C_6H_3$), 68.0 (THF), 28.5 (CH, $^i$Pr), 27.6 (CH, $^i$Pr), 25.4 ($CH_3$, $^i$Pr), 24.7 (THF), 24.4 ($CH_3$, $^i$Pr), 24.1 (pentane), 23.4 ($CH_3$, $^i$Pr), 21.1 ($CH_3$, Me) ppm

**$^7$Li NMR (155.5 MHz, $C_6D_6$, 298K) δ** 2.21 (degradation), 1.38 (**11a**) ppm

**IR spectrum:** ν 1660.9 (s, C=N) $cm^{-1}$

**Melting point analysis**: 146-148 °C

## Figure S11a: ORTEP diagram of [{(MeCN-Dipp)$_2$CH(4-MeC$_6$H$_4$NHCS)}Li·THF] (**11a**)

Molecular structure of **11a**, with selected hydrogen atom displayed and organic groups shown as wire frame for clarity. Thermal ellipsoids are displayed at 40 % probability level.

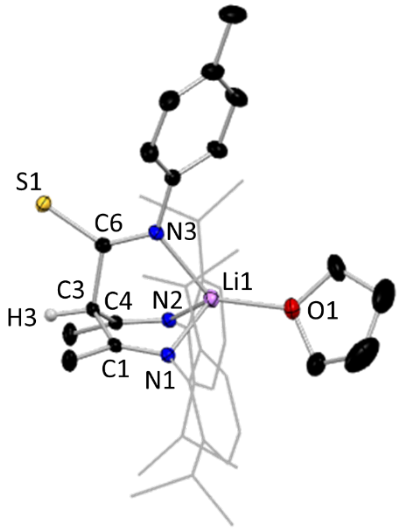


Table S15: Selected bond lengths (Å) and bond angles (°) of **11a**

| Atoms | Bond Lengths (Å) |
|---|---|
| O1-Li1 | 1.926(4) |
| Li1-N1 | 2.067(4) |
| Li1-N2 | 2.046(4) |
| Li1-N3 | 2.071(4) |
| N1-C1 | 1.276(2) |
| C1-C3 | 1.527(2) |
| C3-C4 | 1.523(2) |
| C4-N2 | 1.278(2) |
| C3-C6 | 1.569(2) |
| C6-N3 | 1.298(2) |
| C6-S1 | 1.7081(18) |
| **Atoms** | **Bond Angles (°)** |
| O1-Li1-N1 | 109.91(17) |
| O1-Li1-N2 | 123.28(18) |
| O1-Li1-N3 | 133.34(18) |
| Li1-N3-C6 | 113.79(15) |
| N3-C6-C3 | 113.59(15) |
| N3-C6-S1 | 131.04(14) |
| S1-C6-C3 | 115.35(13) |
| C6-C3-C1 | 108.18(14) |
| C6-C3-C4 | 109.82(14) |
| C3-C4-N2 | 120.57(15) |
| C3-C1-N1 | 120.57(16) |

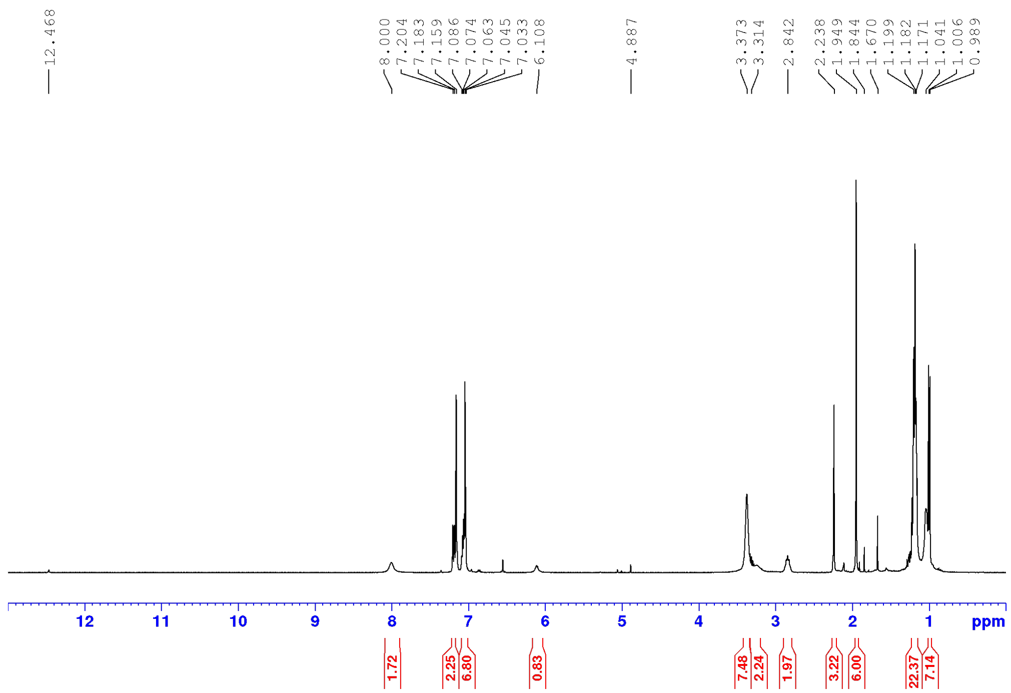

**Figure S11a.1**: $^{1}H$ NMR spectrum in $C_6D_6$ of **11a** [{(MeCN-Dipp)$_2$CH(4-MeC$_6$H$_4$NHCS)}Li·THF]

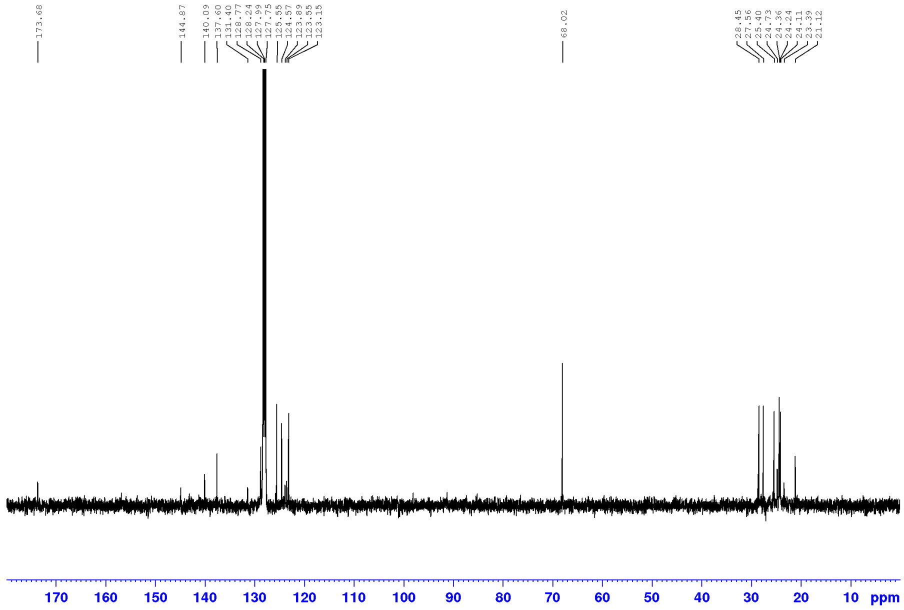

**Figure S11a.2**: $^{13}C\{^{1}H\}$ NMR spectrum in $C_6D_6$ of **11a** [{(MeCN-Dipp)$_2$CH(4-MeC$_6$H$_4$NHCS)}Li·THF]

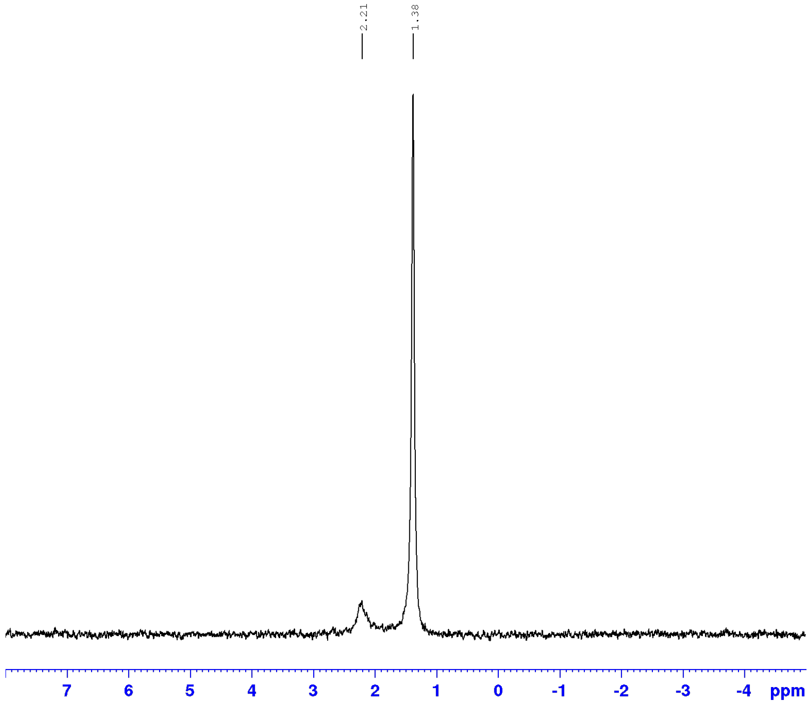


**Figure S11a.3**: $^{7}Li$ NMR spectrum in $C_6D_6$ of **11a** [{(MeCN-Dipp)$_2$CH(4-MeC$_6$H$_4$NHCS)}Li·THF]

## Synthesis of [(MeCNH-Dipp)(MeCN-Dipp)C($^t$BuNHCS)] (**12**)

NacNacLi (0.85 g, 2.0 mmol) dissolved in hexane (10 ml) to produce a yellow solution before $^t$BuNCS (0.25 ml, 2.0 mmol) was added giving an off-white suspension. Suspension stirred (3 hrs) before deionised water (5 ml) was added and mixture exposed to air and stirred overnight. Separation performed using DCM (15 ml), organic layer dried using $MgSO_4$ before product was filtered, dried, and crystallised from methanol. Product was collected as yellow crystals (0.89 g, 1.66 mmol, 83.6 %).

**$^1$H NMR (400.1 MHz, $CDCl_3$, 300 K): δ** 13.01 (s, 1H, NH, NH), 10.81 (unknown impurity), 7.26 ($CDCl_3$), 7.08 (broad m, 8H, CH, $C_6H_3$), 5.24 (unknown impurity), 5.14 (unknown impurity), 3.01 (m, 4H, CH, $^i$Pr), 1.83 (s, 6H, $CH_3$, Me), 1.59 (s, 9H, $CH_3$, $^t$Bu), 1.16 (d, 14H, $CH_3$, $^i$Pr), 1.07 (d, 15H, $CH_3$, $^i$Pr) ppm

Residual NacNacH: 12.05 (NH), 7.20 (CH, $C_6H_3$), 4.82 (γ-CH), 2.92 (CH, $^i$Pr), 2.67 (CH, $^i$Pr), 1.89 ($CH_3$, Me), 1.13-1.02 ($CH_3$, $^i$Pr) ppm

**$^{13}$C{$^1$H} NMR (100.6 MHz, $CDCl_3$, 300 K): δ** 203.1 ($C_{quaternary}$, C=S), 194.2 ($C_{quaternary}$, CMe), 168.2 ($C_{quaternary}$, $C_6H_3$), 159.3 ($C_{quaternary}$, $C_6H_3$), 142.5 ($C_{quaternary}$, $C_6H_3$), 140.1 ($C_{quaternary}$, $C_6H_3$), 137.1 ($C_{quaternary}$, $C_6H_3$), 136.3 ($C_{quaternary}$, $C_6H_3$), 125.5 (CH, $C_6H_3$), 123.3 (CH, $C_6H_3$), 123.2 (CH, $C_6H_3$), 123.1 (CH, $C_6H_3$), 115.4 (unknown impurity), 77.15 ($CDCl_3$), 75.8 (unknown impurity), 55.8 ($C_{quaternary}$, $^t$Bu), 28.4 (CH, $^i$Pr), 28.3 ($CH_3$, $^t$Bu), 27.6 ($CH_3$, $^t$Bu), 24.4 ($CH_3$, $^i$Pr), 23.4 ($CH_3$, $^i$Pr), 18.3 ($CH_3$, Me) ppm

Residual NacNacH: 161.3 ($C_{quaternary}$, CMe), 144.7 ($C_{quaternary}$, $C_6H_3$), 125.2 (CH, $C_6H_3$), 124.3 (CH, $C_6H_3$), 93.4 (CH, γ-C), 23.8 ($CH_3$, $^i$Pr), 23.2 ($CH_3$, $^i$Pr), 23.0 ($CH_3$, $^i$Pr), 20.9 ($CH_3$, Me) ppm

**IR spectrum:** ν 3375.3 (s, NH stretching) $cm^{-1}$

**Melting point analysis**: 123-125 °C

Figure S12: ORTEP diagram of [(MeCNH-Dipp)(MeCN-Dipp)C($^{t}$BuNHCS)] (**12**)

Molecular structure of **12**, with selected hydrogen atoms displayed and organic groups shown as wire frame for clarity. Thermal ellipsoids are displayed at 40 % probability level.

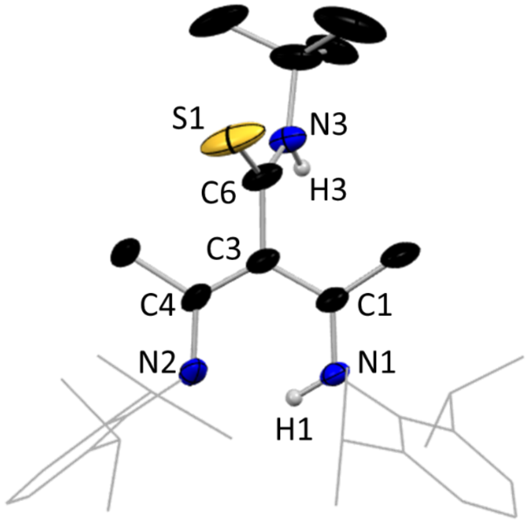


Table S16: Selected bond lengths (Å) and bond angles (°) of **12**

| Atoms | Bond Lengths (Å) |
|---|---|
| N1-C1 | 1.305(14) |
| C1-C3 | 1.384(3) |
| C3-C4 | 1.434(3) |
| C4-N2 | 1.318(3) |
| C3-C6 | 1.506(3) |
| C6-N3 | 1.329(3) |
| C6-S1 | 1.690(4) |
| **Atoms** | **Bond Angles (°)** |
| C1-C3-C6 | 118.30(19) |
| C4-C3-C6 | 117.4(2) |
| S1-C6-N3 | 121.72(19) |

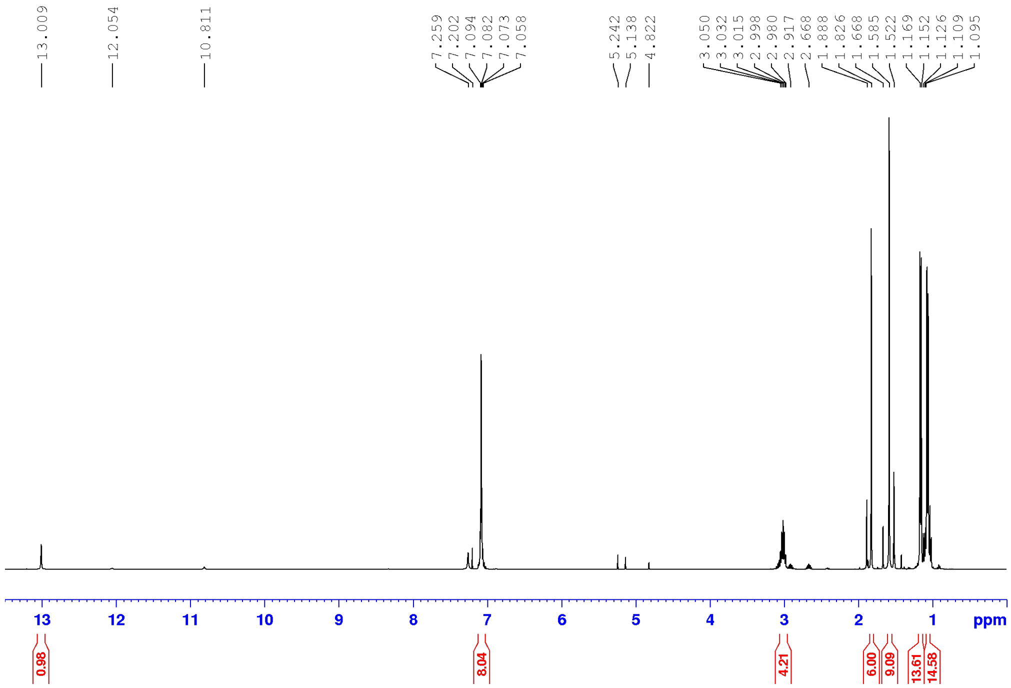


**Figure S12.1**: $^{1}H$ NMR spectrum in $CDCl_3$ of **12** [(MeCNH-Dipp)(MeCN-Dipp)C($^{t}$BuNHCS)]

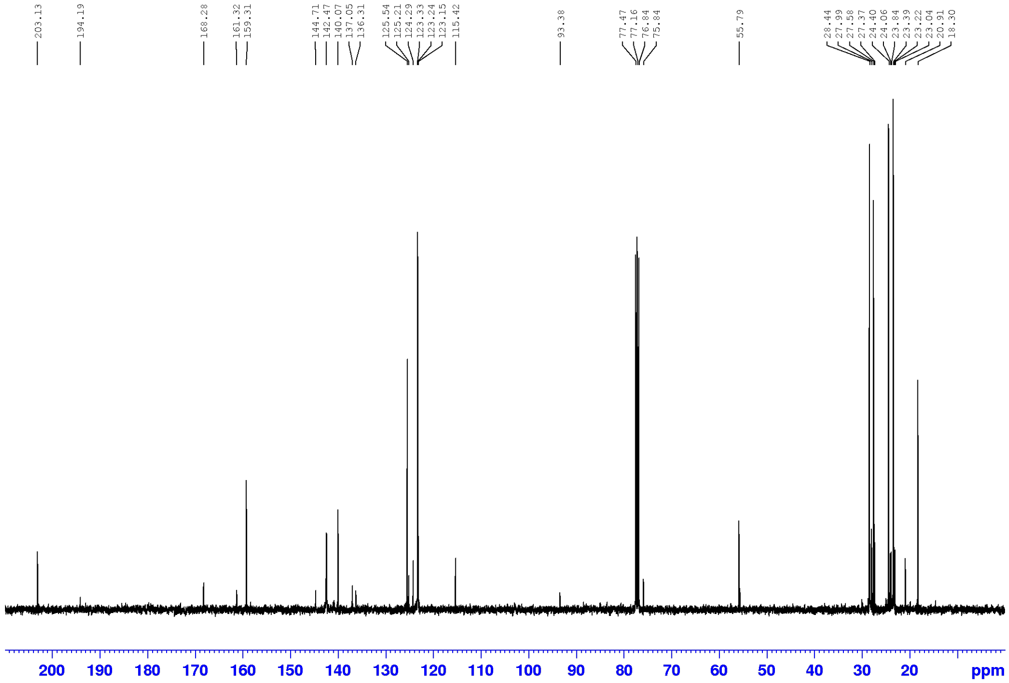


**Figure 12.2**: $^{13}C\{^{1}H\}$ NMR spectrum in $CDCl_3$ of **12** [(MeCNH-Dipp)(MeCN-Dipp)C($^{t}$BuNHCS)]

## Synthesis of [(MeCNH-Dipp)(MeCN-Dipp)C(PhNHCS)] (**13**)

NacNacLi (0.85 g, 2.0 mmol) dissolved in hexane (10 ml) to produce a yellow solution before PhNCS (0.24 ml, 2.0 mmol) was added giving an off-white suspension. Suspension stirred (3 hrs) before deionised water (5 ml) was added and reaction exposed to air and stirred overnight. Separation performed using DCM (15 ml), organic layer dried using $MgSO_4$ before product was filtered, dried, and crystallised from methanol. Product was collected as yellow crystals (1.01 g, 1.82 mmol, 91.0 %).

**$^1$H NMR (400.1 MHz, $CDCl_3$, 300 K): δ** 13.71 (s, 1H, NH, NH), 10.18 (s, 1H, NH, C(=S)NH), 9.05 (unknown impurity), 7.51 (broad m, 2H, CH, $C_6H_3$), 7.39 (broad m, 4H, CH, $C_6H_3$), 7.29 (m, 3H, CH, Ph/$C_6H_4$), 7.26 ($CDCl_3$), 7.21 (m, 2H, CH, $C_6H_3$/$C_6H_4$), 3.22 (sep, 3H, CH, $^i$Pr), 2.90 (sep, 2H, CH, $^i$Pr), 2.01 (s, 6H, $CH_3$, Me), 1.34 (d, 6H, $CH_3$, $^i$Pr), 1.25 (d, 6H, $CH_3$, $^i$Pr), 1.20 (d, 6H, $CH_3$, $^i$Pr), 1.14 (d, 6H, $CH_3$, $^i$Pr) ppm

Residual NacNacH: 13.49 (NH), 7.93 (CH, Ph), 5.53 (γ-CH), 2.12 ($CH_3$, Me), 1.38-1.36 ($CH_3$, $^i$Pr), 1.29-1.27 ($CH_3$, $^i$Pr) ppm

**$^{13}$C{$^1$H} NMR (100.6 MHz, $CDCl_3$, 300 K): δ** 205.6 ($C_{quaternary}$, C=S), 159.7 ($C_{quaternary}$, CMe), 142.6 ($C_{quaternary}$, $C_6H_3$), 141.8 ($C_{quaternary}$, $C_6H_3$), 139.5 ($C_{quaternary}$, $C_6H_3$/$C_6H_4$), 139.3 ($C_{quaternary}$, $C_6H_3$/$C_6H_4$), 129.3 (CH, $C_6H_3$), 126.2 (CH, $C_6H_3$), 125.7 (CH, $C_6H_3$), 123.5 (CH, $C_6H_3$/$C_6H_4$), 123.2 (CH, $C_6H_3$/$C_6H_4$), 122.3 (CH, $C_6H_3$/$C_6H_4$), 107.6 ($C_{quaternary}$, γ-C), 77.16 ($CDCl_3$), 28.4 (CH, $^i$Pr), 28.2 (CH, $^i$Pr), 24.2 ($CH_3$, $^i$Pr), 24.4 ($CH_3$, $^i$Pr), 23.6 ($CH_3$, $^i$Pr), 23.4 ($CH_3$, $^i$Pr), 19.2 ($CH_3$, Me) ppm

**IR spectrum:** ν 3175.2 (s, NH stretching), 3122.2 (s, NH stretching) $cm^{-1}$

**Melting point analysis**: 122-124 °C

The crystals of compound **13** proved of insufficient quality for X-ray crystallographic determination.

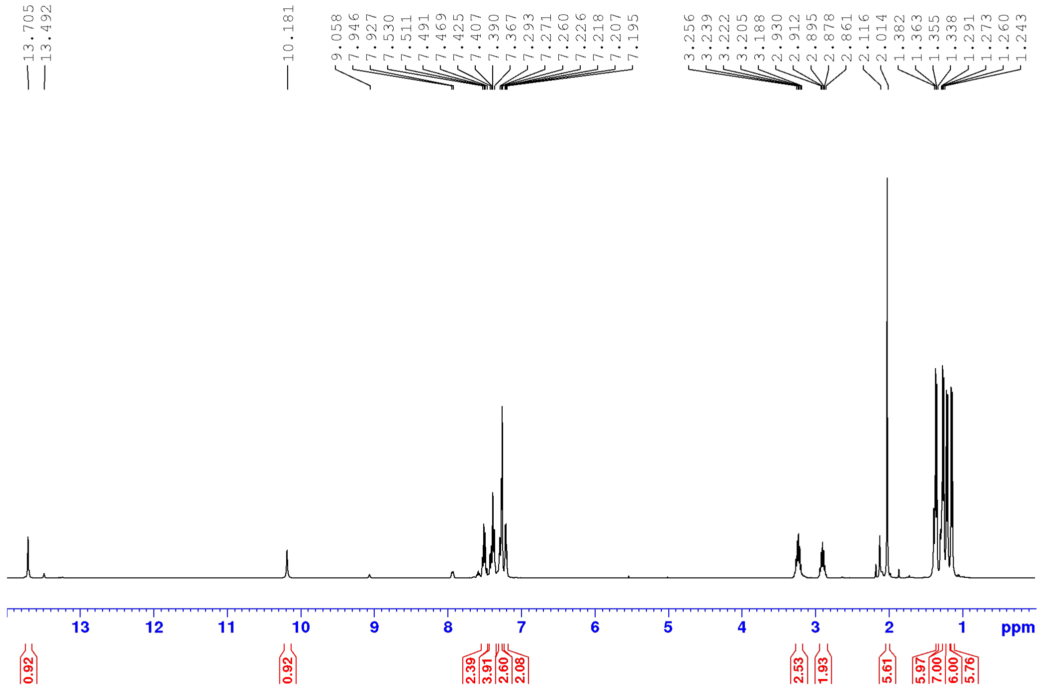


**Figure S13.1**: $^{1}$H NMR spectrum in $CDCl_3$ of **13** [(MeCNH-Dipp)(MeCN-Dipp)C(PhNHCS)]

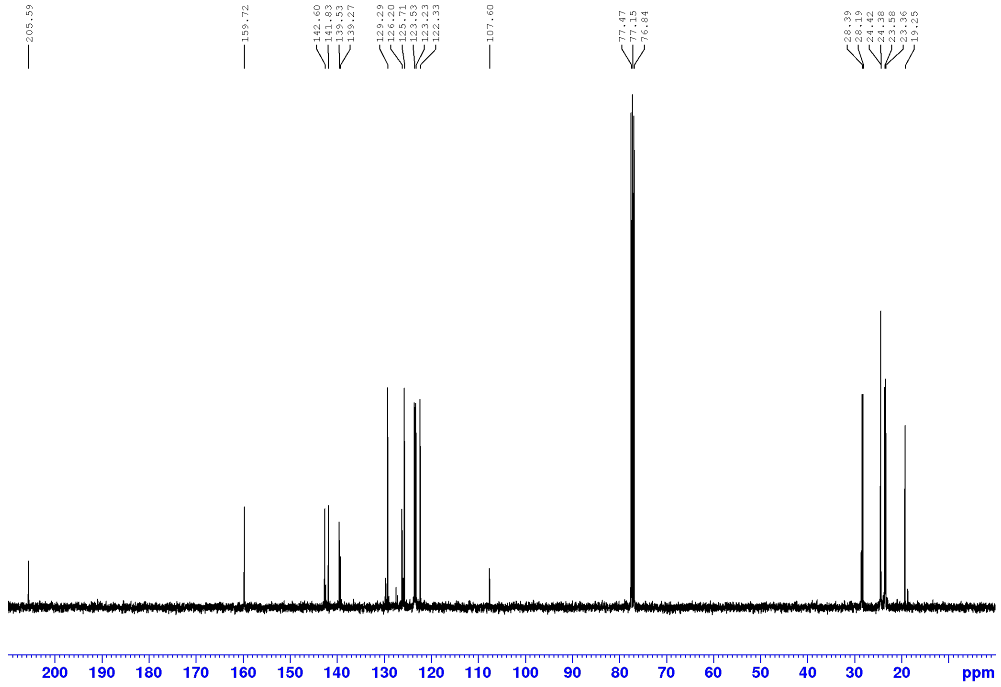


**Figure S13.2**: $^{13}$C{$^{1}$H} NMR spectrum in $CDCl_3$ of **13** [(MeCNH-Dipp)(MeCN-Dipp)C(PhNHCS)]

## Synthesis of [{(MeCN-Dipp)$_2$CH(PhNHCS)}Li·THF] (**13a**)

NacNacLi (0.213 g, 0.5 mmol) dissolved in hexane (5 ml) to produce a yellow solution before PhNCS (60 µl, 0.5 mmol) was added giving a white suspension. Suspension stirred overnight before THF added to give a yellow solution which was placed in -20°C freezer. Product was collected as pale-yellow crystals (0.282 g, 0.45 mmol, 89.2 %).

**$^1$H NMR (400.1 MHz, $C_6D_6$, 300 K): δ** 8.06 (broad s, 2H, CH, Ph), 7.38 (m, 2H, CH, Ph), 7.16 ($C_6D_6$), 7.04 (broad m, 7H, CH, $C_6H_3$), 6.07 (broad s, 1H, CH, γ-CH), 3.33 (broad m, 8H, $CH_2$, THF), 2.83 (m, 2H, CH, $^i$Pr), 1.94 (s, 6H, $CH_3$, Me), 1.19 (broad m, 7H, $CH_2$, THF), 1.16 (broad m, 12H, $CH_3$, $^i$Pr), 1.00 (broad d, 12H, $CH_3$, $^i$Pr), 0.89 (hexane) ppm

**$^{13}$C{$^1$H} NMR (100.6 MHz, $C_6D_6$, 300 K): δ** 173.7 ($C_{quaternary}$, CMe), 144.9 ($C_{quaternary}$, $C_6H_3$), 140.1 ($C_{quaternary}$, $C_6H_3$), 137.7 ($C_{quaternary}$, Ph), 128.1 ($C_6D_6$), 125.6 (CH, $C_6H_3$), 124.6 (CH, $C_6H_3$), 124.0 (CH, $C_6H_3$), 123.2 (CH, Ph), 122.6 (CH, Ph), 68.1 (THF), 28.5 (CH, $^i$Pr), 27.6 (CH, $^i$Pr), 25.4 ($CH_3$, Me), 24.7 (THF), 24.4 ($CH_3$, $^i$Pr), 24.3 ($CH_3$, $^i$Pr), 24.2 ($CH_3$, $^i$Pr) ppm

**$^7$Li NMR (155.5 MHz, $C_6D_6$, 298K) δ** 2.21 (degradation),1.37 (**13a**) ppm

**IR spectrum:** ν 1661.9 (s, C=N stretching) $cm^{-1}$

**Melting point analysis**: 210-212 °C

Figure S13a: ORTEP diagram of [{(MeCN-Dipp)$_2$CH(PhNHCS)}Li·THF] (**13a**)

Molecular structure of **13a**, with selected hydrogen atom displayed and organic groups shown as wire frame for clarity. Thermal ellipsoids are displayed at 40 % probability level. Solvent molecules omitted.

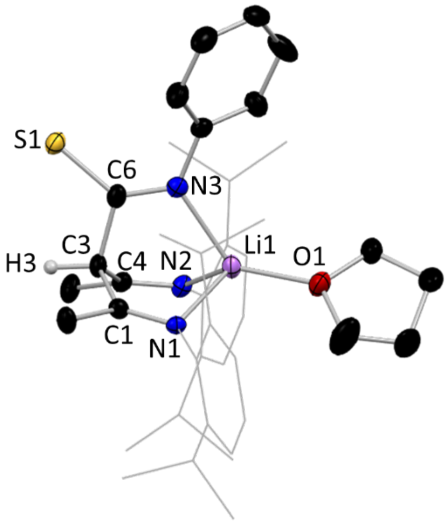


Table S17: Selected bond lengths (Å) and bond angles (°) of **13a**

| **Atoms** | **Bond Lengths (Å)** |
|---|---|
| O1-Li1 | 1.913(2) |
| Li1-N1 | 2.065(2) |
| Li1-N2 | 2.054(2) |
| Li1-N3 | 2.069(2) |
| N1-C1 | 1.2781(16) |
| C1-C3 | 1.5249(16) |
| C3-C4 | 1.5233(16) |
| C4-N2 | 1.2752(16) |
| C3-C6 | 1.5654(17) |
| C6-N3 | 1.3050(16) |
| C6-S1 | 1.7087(13) |
| **Atoms** | **Bond Angles (°)** |
| O1-Li1-N1 | 112.64(11) |
| O1-Li1-N2 | 121.96(12) |
| O1-Li1-N3 | 132.34(13) |
| Li1-N3-C6 | 113.56(11) |
| N3-C6-C3 | 113.70(11) |
| N3-C6-S1 | 130.85(10) |
| S1-C6-C3 | 115.43(9) |
| C6-C3-C1 | 108.87(9) |
| C6-C3-C4 | 109.06(9) |
| C3-C4-N2 | 120.48(11) |
| C3-C1-N1 | 120.70(10) |

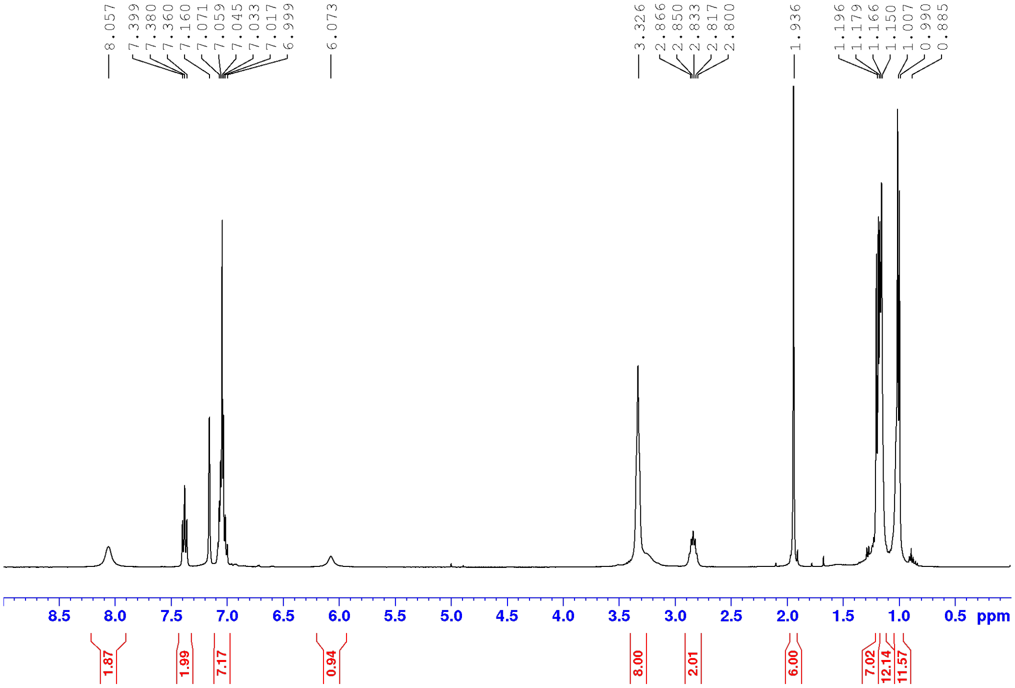


**Figure S13a.1**: $^{1}H$ NMR spectrum in $C_6D_6$ of **13a** [{(MeCN-Dipp)$_2$CH(PhNHCS)}Li·THF]

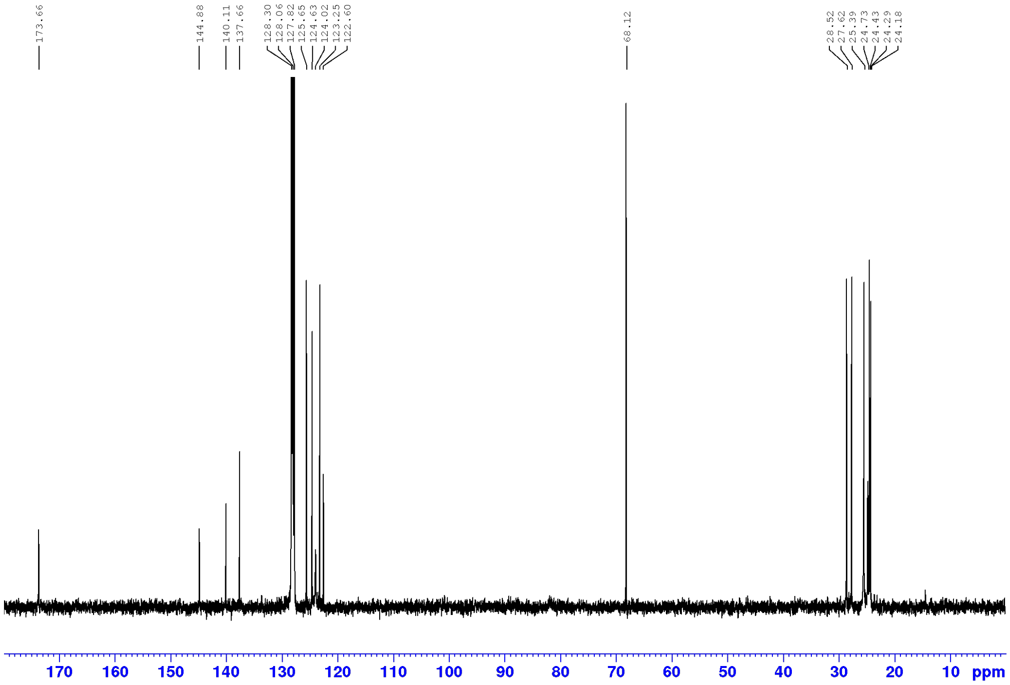


**Figure S13a.2**: $^{13}C\{^{1}H\}$ NMR spectrum in $C_6D_6$ of **13a** [{(MeCN-Dipp)$_2$CH(PhNHCS)}Li·THF]

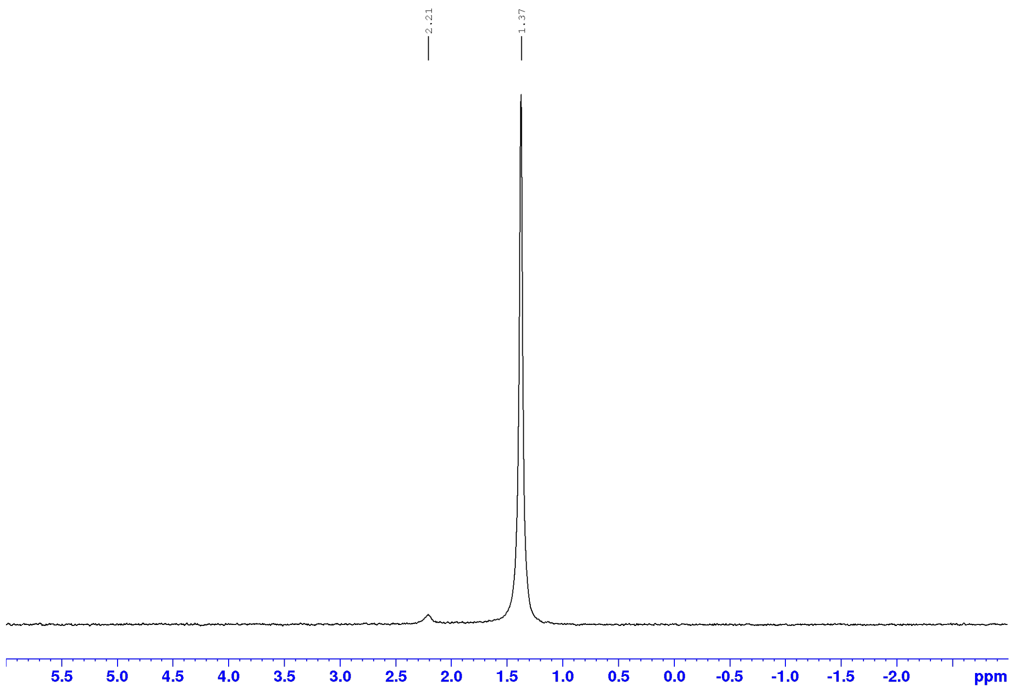


**Figure S13a.3**: $^{7}Li$ NMR spectrum in $C_6D_6$ of **13a** [{(MeCN-Dipp)$_2$CH(PhNHCS)}Li·THF]

## Synthesis of [(MeCNH-Dipp)(MeCN-Dipp)C(EtNHCS)] (**14**)

NacNacLi (0.85 g, 2.0 mmol) dissolved in hexane (10 ml) to produce a yellow solution before EtNCS (0.18 ml, 2.0 mmol) was added giving an off-white suspension. Suspension stirred (3 hrs) before deionised water (5 ml) was added and mixture exposed to air and stirred overnight. Separation performed using DCM (15 ml), organic layer dried using $MgSO_4$ before product was filtered, dried, and crystallised from methanol. Product was collected as yellow crystals (0.81 g, 1.60 mmol, 79.8 %).

**$^1$H NMR (400.1 MHz, $C_6D_6$, 300 K): δ** 13.60 (s, 1H, NH, NH), 7.20-7.17 (broad m, 2H, CH, $C_6H_3$), 7.16 ($C_6D_6$), 6.63 (m, 1H, NH, C(=S)NH), 3.47-3.31 (broad m, 6H, CH, $^i$Pr), 1.99 (s, 6H, $CH_3$, Me), 1.28 (d, 13H, $CH_3$, $^i$Pr), 1.18 (d, 12H, $CH_3$, $^i$Pr), 0.69 (m, 3H, $CH_3$, Et) ppm

Residual NacNacH: 1.95 ($CH_3$, Me), 1.23-1.11 ($CH_3$, $^i$Pr) ppm

**$^{13}$C{$^1$H} NMR (100.6 MHz, $C_6D_6$, 300 K): δ** 203.2 ($C_{quaternary}$, C=S), 159.8 ($C_{quaternary}$, CMe), 142.7 ($C_{quaternary}$, $^i$Pr), 140.5 ($C_{quaternary}$, $C_6H_3$), 128.0 ($C_6D_6$), 126.1 (CH, $C_6H_3$), 123.8 (CH, $C_6H_3$), 113.7 ($C_{quaternary}$, γ-C), 40.7 (DCM), 28.8 (CH, $^i$Pr), 28.3 (CH, $^i$Pr), 24.5 ($CH_3$, $^i$Pr), 23.5 ($CH_3$, $^i$Pr), 18.5 ($CH_3$, Me), 12.3 ($CH_3$, Et) ppm

**IR spectrum:** ν 3198.8 (broad s, NH stretching) $cm^{-1}$

**Melting point analysis**: 144-146 °C

## Figure S14: ORTEP diagram of [(MeCNH-Dipp)(MeCN-Dipp)C(EtNHCS)] (**14**)

Molecular structure of **14**, with selected hydrogen atoms displayed and organic groups shown as wire frame for clarity. Thermal ellipsoids are displayed at 40 % probability level, second molecule not shown.

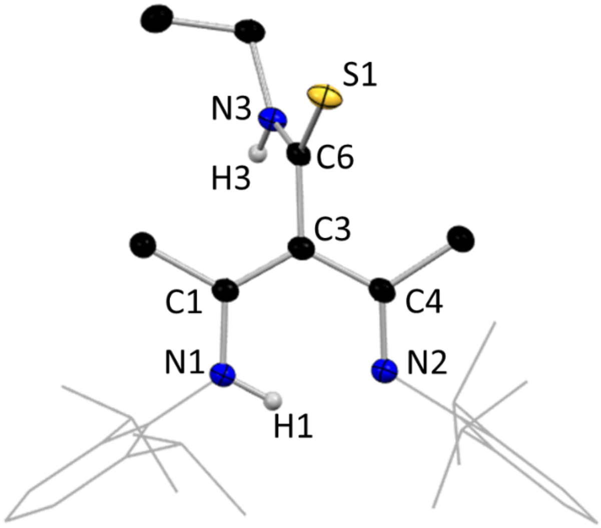


Table S18: Selected bond lengths (Å) and bond angles (°) of **14**

| **Atoms** | **Bond Lengths (Å)** |
|---|---|
| N1-C1 | 1.3538(18) |
| C1-C3 | 1.3954(19) |
| C3-C4 | 1.456(2) |
| C4-N2 | 1.3013(19) |
| C3-C6 | 1.3252(19) |
| C6-N3 | 1.3252(19) |
| C6-S1 | 1.6859(14) |
| **Atoms** | **Bond Angles (°)** |
| C1-C3-C6 | 117.42(12) |
| C4-C3-C6 | 119.38(12) |
| S1-C6-N3 | 122.02(11) |

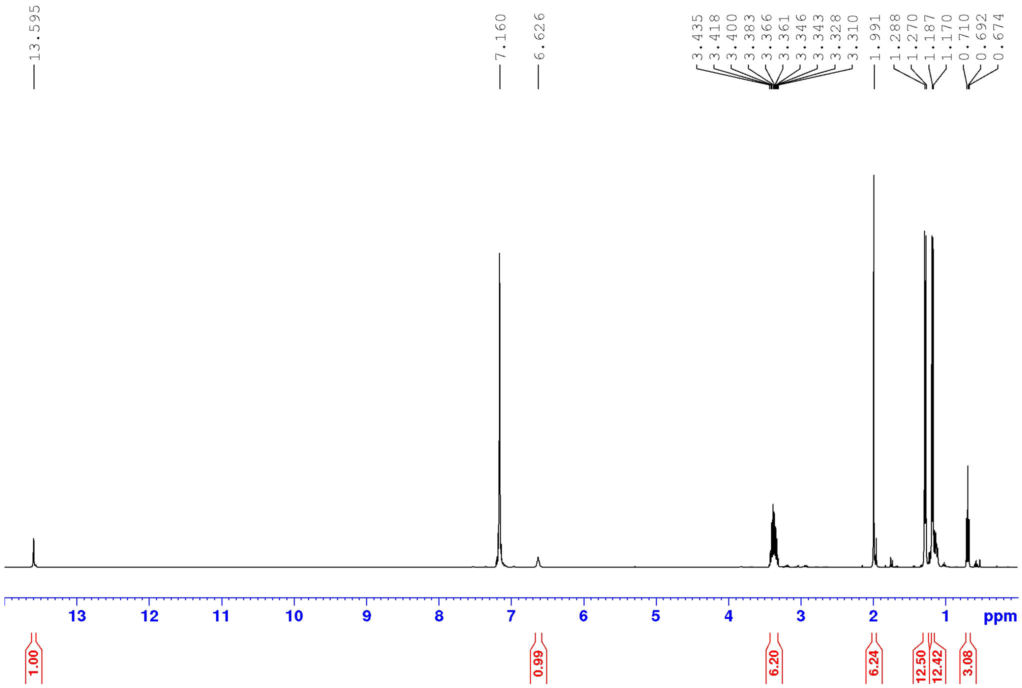


**Figure S14.1**: $^{1}H$ NMR spectrum in $C_6D_6$ of **14** [(MeCNH-Dipp)(MeCN-Dipp)C(EtNHCS)]

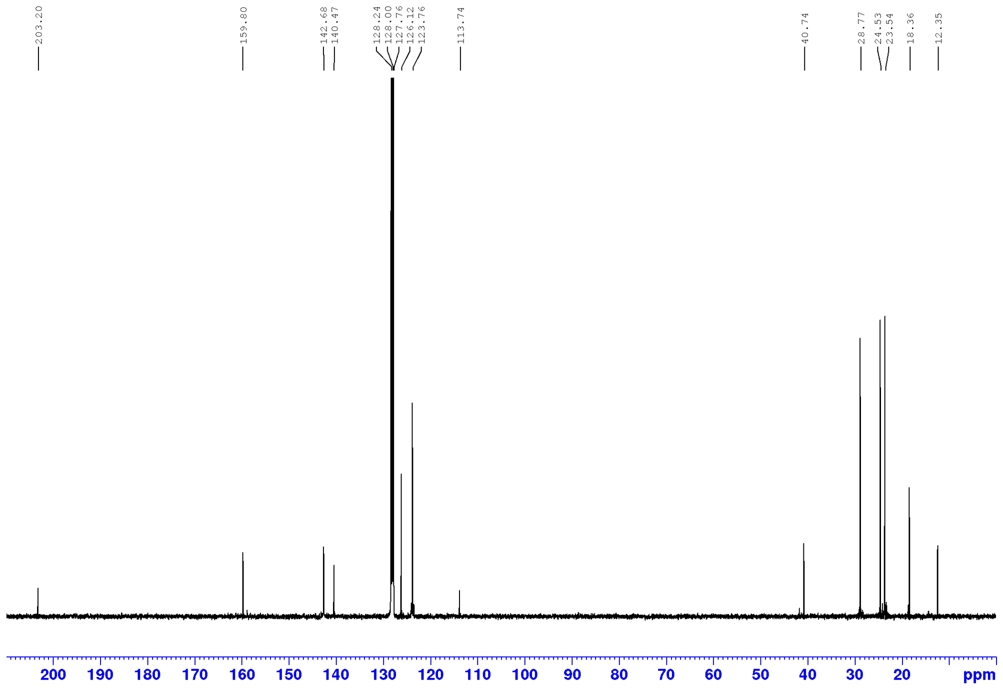


**Figure 14.2**: $^{13}C\{^{1}H\}$ NMR spectrum in $C_6D_6$ of **14** [(MeCNH-Dipp)(MeCN-Dipp)C(EtNHCS)]

## Synthesis of [{$(MeCN\text{-}Dipp)_2CH(EtNCS)$}Li] (**14a**)

NacNacLi (0.213 g, 0.5 mmol) dissolved in hexane (3 ml) to produce a yellow solution before EtNCS (44 µl, 0.5 mmol) was added giving an off-white suspension which was stirred overnight. Product dried in vacuo (0.192 g, 0.41 mmol, 81.6 %). Crystals of **14a** were obtained from a suspension of hexane layered with THF, after several days a small number of single crystals of **14a** were found to have crystallised above the suspension mixture.

NMR spectroscopic data listed are of THF-coordinated **14a** due to the poor solubility of the unsolvated polymeric parent compound.

**$^1$H NMR (400.1 MHz, $C_6D_6$, 300 K): δ** 7.28 (s, 3H, CH, $C_6H_3$), 7.22 (s, 3H, CH, $C_6H_3$), 7.17 (s, 2H, CH, $C_6H_3$), 7.16 ($C_6D_6$), 6.14 (broad s, 1H, CH, γ-CH), 5.12 (unknown impurity), 4.10 (broad m, 2H, CH, $^i$Pr),3.56 (THF), 3.21 (m, 2H, CH, $^i$Pr), 2.92 (m, 2H, CH, $^i$Pr), 2.03 (s, 6H, $CH_3$, Me), 1.55 (broad m, 4H, $CH_2$, Et), 1.42 (THF), 1.33-1.27 (broad m, 27H, $CH_3$, $^i$Pr), 1.11 (m, 7H, $CH_3$, Et) ppm

Residual NacNacH: 12.58 (NH), 5.01 (γ-CH), 3.43 (CH, $^i$Pr), 1.79 ($CH_3$, Me), 1.13-1.02 ($CH_3$, $^i$Pr) ppm

**$^{13}$C{$^1$H} NMR (100.6 MHz, $C_6D_6$, 300 K): δ** 172.9 ($C_{quaternary}$, C=S), 160.7 ($C_{quaternary}$, CMe), 144.2 ($C_{quaternary}$, $C_6H_3$), 141.9 ($C_{quaternary}$, $C_6H_3$), 140.4 ($C_{quaternary}$, $C_6H_3$), 139.1 ($C_{quaternary}$, $C_6H_3$), 136.8 ($C_{quaternary}$, $C_6H_3$), 128.0 ($C_6D_6$), 125.0 (CH, $C_6H_3$), 124.6 (CH, $C_6H_3$), 123.6 (CH, $C_6H_3$), 122.7 (CH, $C_6H_3$), 122.4 (CH, $C_6H_3$), 93.4 ($C_{quaternary}$, γ-C), 67.0 (THF), 27.8 ($CH_2$, Et), 27.5 (CH, $^i$Pr), 27.2 (CH, $^i$Pr), 26.7 (CH, $^i$Pr), 24.9 (THF), 23.6 ($CH_3$, $^i$Pr), 23.5 ($CH_3$, $^i$Pr), 23.3 ($CH_3$, $^i$Pr), 22.6 ($CH_3$, Et), 19.9 ($CH_3$, Me), 15.5 ($CH_3$, Et) ppm

**$^7$Li NMR (155.5 MHz, $C_6D_6$, 298K) δ** 2.19 (degradation),1.16 (**14a·THF**) ppm

**IR spectrum:** ν 1672.5 (s, C=N stretching) $cm^{-1}$

**Melting point analysis**: 173-175 °C

Figure S14a: ORTEP diagram of [{(MeCN-Dipp)$_2$CH(EtNCS)}Li] (**14a**)

Molecular structure of **14a**, with selected hydrogen atom displayed and organic groups shown as wire frame for clarity. Thermal ellipsoids are displayed at 40 % probability level.

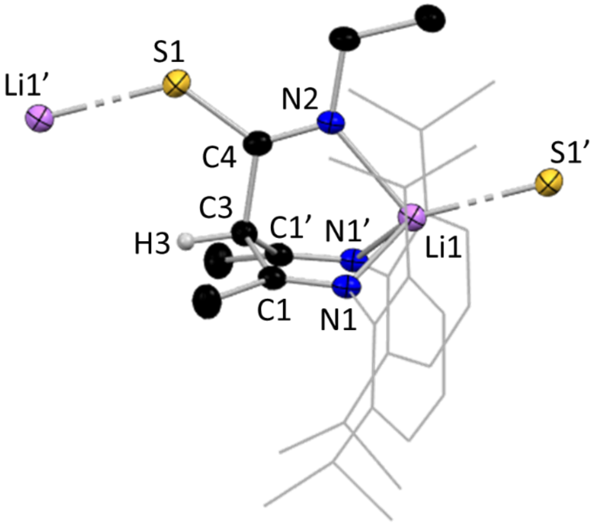


Table S19: Selected bond lengths (Å) and bond angles (°) of **14a**

| Atoms | Bond Lengths (Å) |
|---|---|
| Li1-N1 | 2.064(5) |
| Li1-N1' | 2.064(5) |
| Li1-N2 | 2.126(7) |
| Li1-S1' | 2.399(6) |
| N1-C1 | 1.280(4) |
| C1-C3 | 1.524(3) |
| C3-C4 | 1.559(5) |
| C4-N2 | 1.294(5) |
| C4-S1 | 1.714(4) |
| S1-Li1' | 2.399(7) |
| **Atoms** | **Bond Angles (°)** |
| S1'-Li1-N2 | 115.5(3) |
| S1'-Li1-N1 | 127.66(19) |
| S1'-Li1-N1' | 127.66(19) |
| Li1-N2-C4 | 113.7(3) |
| N2-C4-S1 | 126.8(3) |
| N2-C4-C3 | 115.1(3) |
| C4-C3-C1 | 108.29(18) |
| C3-C1-N1 | 120.0(2) |
| C1-N1-Li1 | 110.9(2) |
| C4-S1-Li1' | 128.6(2) |

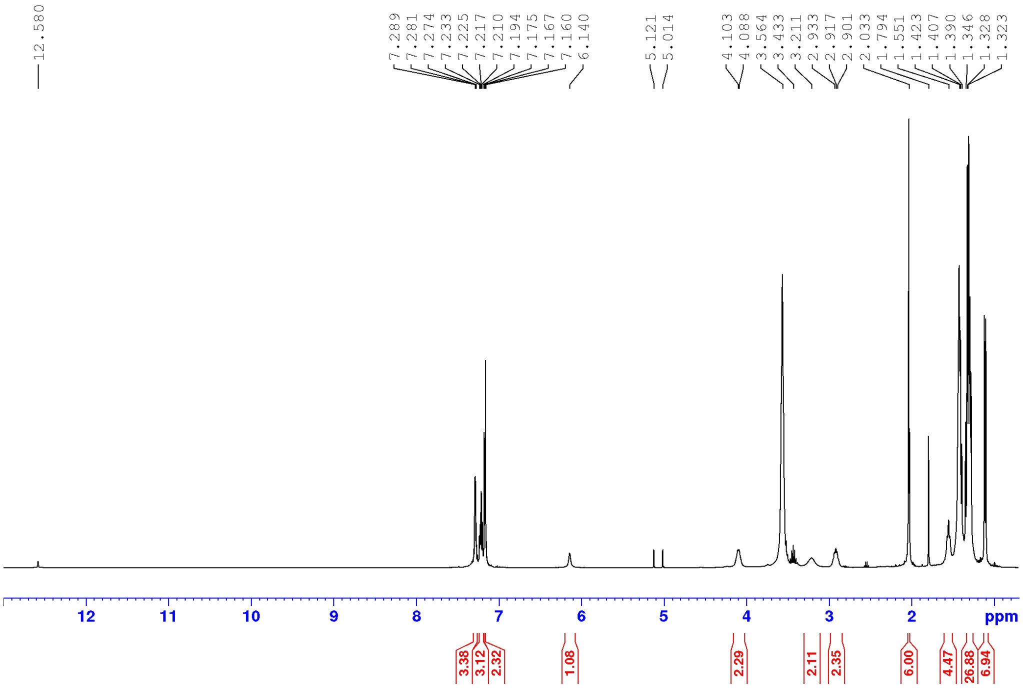


**Figure S14a.1**: $^{1}$H NMR spectrum in $C_6D_6$ of **14a·THF** [{(MeCN-Dipp)$_2$CH(EtNCS)}Li·THF]

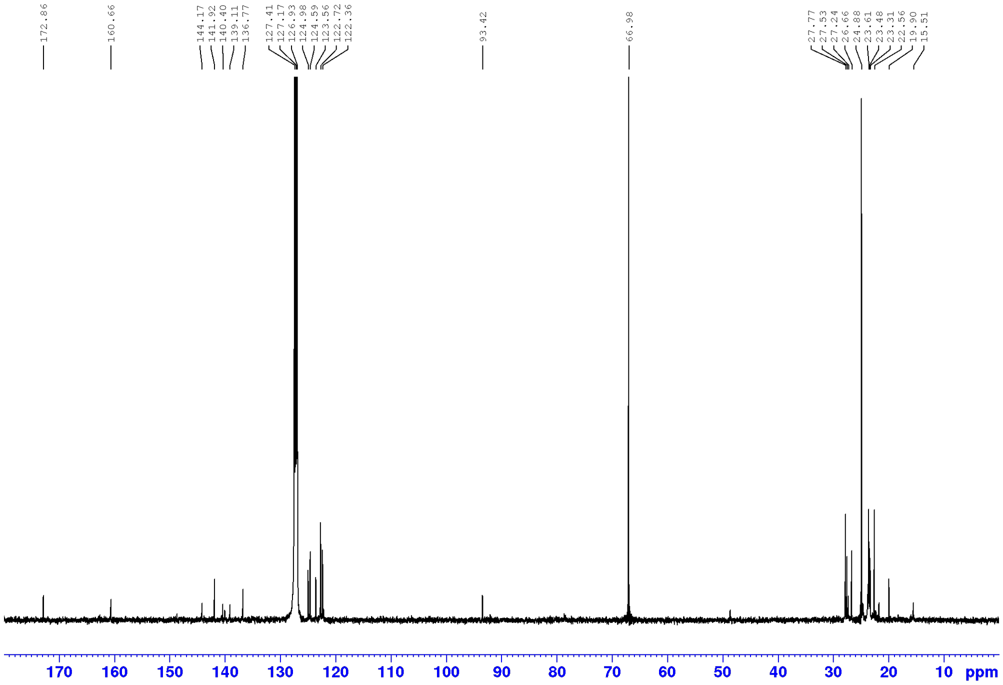


**Figure 14a.2**: $^{13}$C{$^{1}$H} NMR spectrum in $C_6D_6$ of **14a·THF** [{(MeCN-Dipp)$_2$CH(EtNCS)}Li·THF]

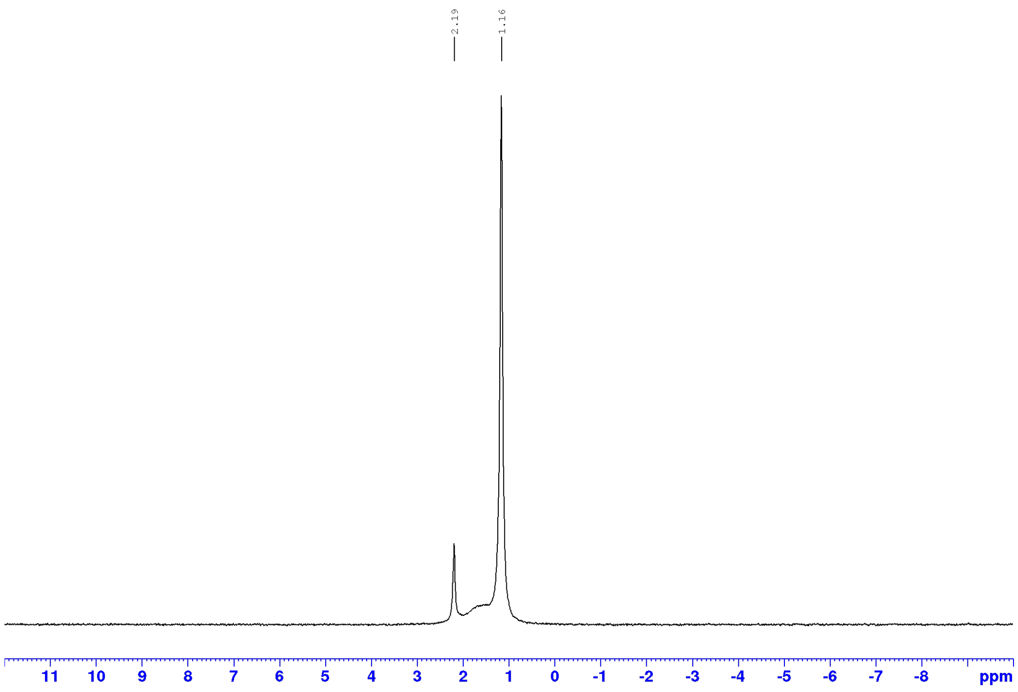


**Figure S14a.3**: $^{7}Li$ NMR spectrum in $C_6D_6$ of **14a·THF** [{(MeCN-Dipp)$_2$CH(EtNCS)}Li·THF]

## Synthesis of [(N-Dipp)C($CH_3$)C(C(O)$CH_3$)C(NPh)S] (**15**)

Only a single crystal of **15** was isolated from a crystalline sample of compound **13** and thus there was insufficient material available for spectroscopic characterisation.

## Figure S15: ORTEP diagram of [(N-Dipp)C($CH_3$)C(C(O)$CH_3$)C(NPh)S] (**15**)

Molecular structure of **15**, with the NacNac Dipp group shown as wire frame for clarity. Thermal ellipsoids are displayed at 40 % probability level.

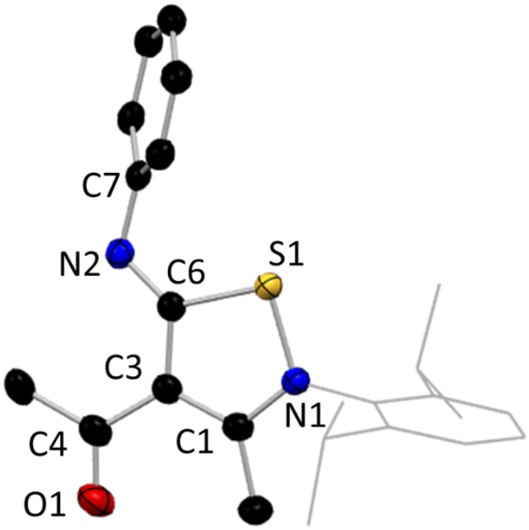


Table S20: Selected bond lengths (Å) and bond angles (°) of **15**

| Atoms | Bond Lengths (Å) |
|---|---|
| S1-N1 | 1.7171(16) |
| S1-C6 | 1.7988(18) |
| C6-N2 | 1.281(2) |
| N2-C7 | 1.417(3) |
| C6-C3 | 1.446(3) |
| C3-C4 | 1.470(3) |
| C4-O1 | 1.221(3) |
| C3-C1 | 1.392(3) |
| C1-N1 | 1.344(2) |
| **Atoms** | **Bond Angles (°)** |
| C1-C3-C6 | 113.15(16) |
| C1-C3-C4 | 122.00(18) |
| C4-C3-C6 | 124.79(18) |
| N2-C6-C3 | 129.52(17) |
| C3-C6-S1 | 107.52(13) |
| N2-C6-S1 | 122.88(15) |
| C3-C1-N1 | 114.05(17) |

## Synthesis of [(MeCNH-Dipp)(MeCN-Dipp)C(NH-$^{i}$PrCN-$^{i}$Pr)] (**16**)

NacNacH (0.410 g, 1.0 mmol) placed in a Schlenk tube, dissolved in hexane (5 ml) and $^{n}$BuLi (0.71 ml, 1.2 M, 1.0 mmol) added to give a yellow solution. PMDETA (0.21 ml, 1.0 mmol) and DIC (0.16 ml, 1.0 mmol) added before reaction refluxed (3 hrs). A colour change from a yellow to orange solution occurred, reaction placed in -18 °C freezer throughout the Covid-19 pandemic lockdown. After 18 months a small crop of large colourless crystals formed, no yield was recorded.

**$^{1}$H NMR (400.1 MHz, $CDCl_3$, 300 K): δ** 13.11 (s, 1H, NH, NH), 7.26 ($CDCl_3$), 7.13 (m, 6H, CH, $C_6H_3$), 4.11 (s, 1H, CH, $^{i}$Pr), 3.69 (s, 1H, CH, $^{i}$Pr), 3.09 (sep, 4H, CH, $^{i}$Pr), 1.74 (s, 6H, $CH_3$, Me), 1.20 (d, 14H, $CH_3$, DIC), 1.14 (d, 23H, $CH_3$, $^{i}$Pr) ppm

**$^{13}$C{$^{1}$H} NMR (100.6 MHz, $CDCl_3$, 300 K): δ** 160.6 ($C_{quaternary}$, CMe), 142.4 ($C_{quaternary}$, $^{i}$Pr), 140.3 ($C_{quaternary}$, $C_6H_3$), 125.5 (CH, $C_6H_3$), 123.3 (CH, $C_6H_3$), 77.2 ($CDCl_3$), 28.5 (CH, $^{i}$Pr), 24.4 ($CH_3$, $^{i}$Pr), 23.4 ($CH_3$, DIC), 18.8 ($CH_3$, Me) ppm

**IR spectrum:** ν 3449.1 (s, NH stretching), 3357.9 (broad s, NH stretching) $cm^{-1}$

**Melting point analysis**: 112-114 °C

## Figure S16: ORTEP diagram of [(MeCNH-Dipp)(MeCN-Dipp)C(NH-$^{i}$PrCN-$^{i}$Pr)] (**16**)

Molecular structure of **16**, with selected hydrogen atoms displayed and the NacNac Dipp groups shown as wire frame for clarity. Thermal ellipsoids are displayed at 40 % probability level.

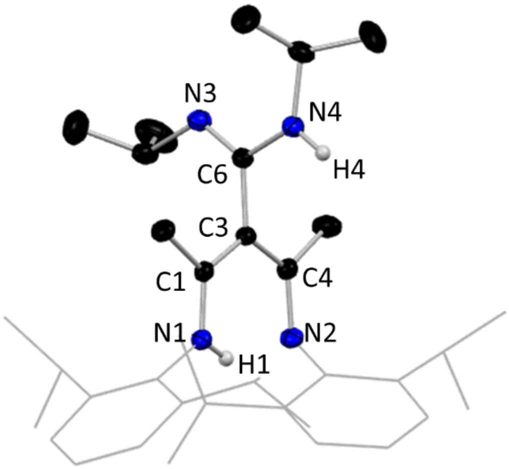


Table S21: Selected bond lengths (Å) and bond angles (°) of **16**

| Atoms | Bond Lengths (Å) |
|---|---|
| N1-C1 | 1.319(17) |
| C1-C3 | 1.420(17) |
| C3-C4 | 1.411(17) |
| C4-N2 | 1.336(17) |
| C3-C6 | 1.506(17) |
| C6-N3 | 1.372(17) |
| C6-N4 | 1.282(17) |
| N3-C34 | 1.4626(16) |
| N4-C31 | 1.4581(16) |
| **Atoms** | **Bond Angles (°)** |
| C1-C3-C6 | 118.06(10) |
| C4-C3-C6 | 117.90(10) |
| N3-C6-N4 | 119.45(11) |

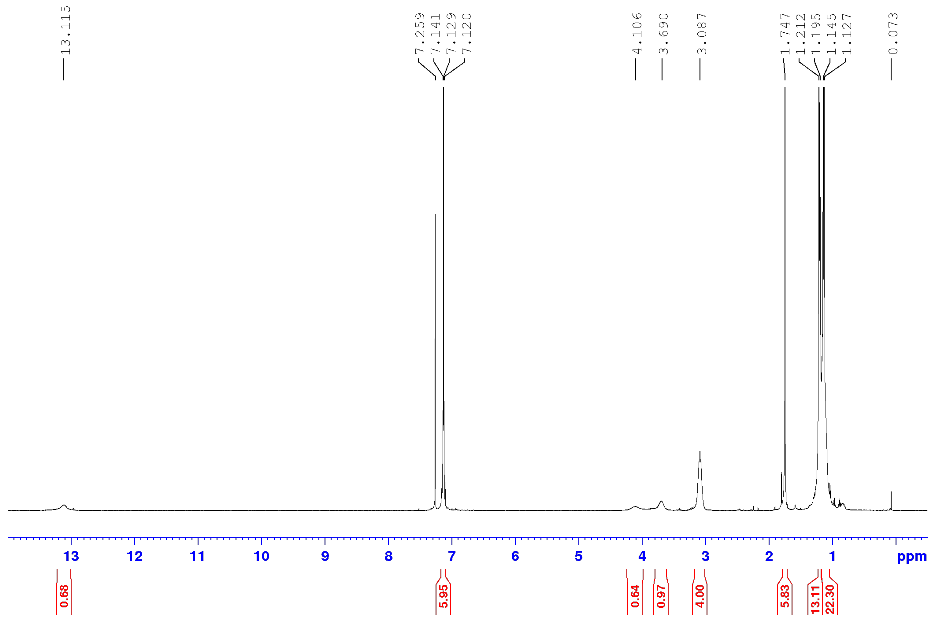


**Figure S16.1**: $^{1}$H NMR spectrum in $CDCl_3$ of **16** [(MeCNH-Dipp)(MeCN-Dipp)C(NH-$^{i}$PrCN-$^{i}$Pr)]

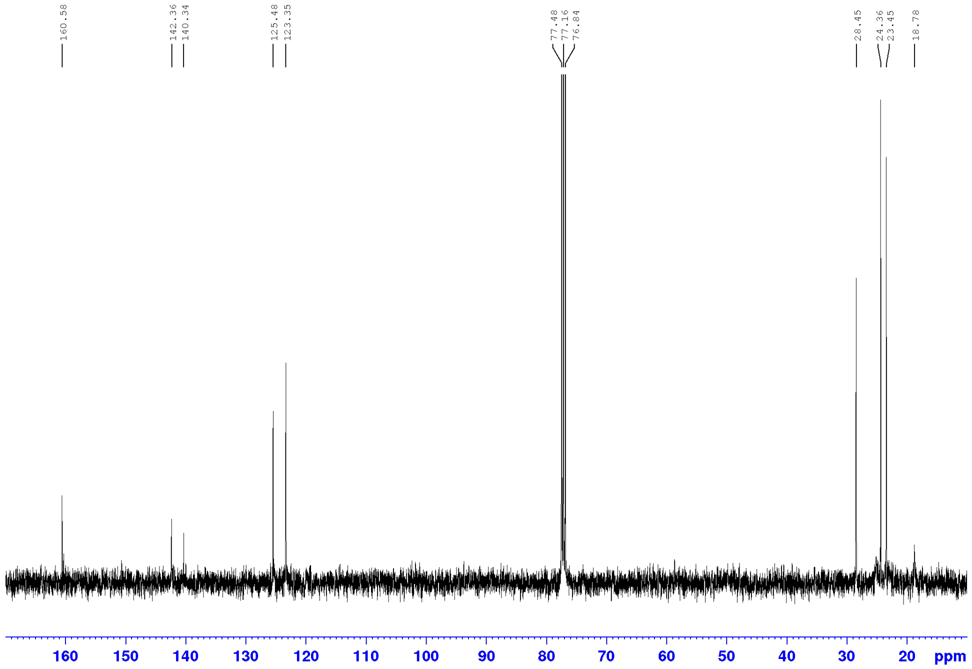


**Figure S16.2**: $^{13}C\{^{1}H\}$ NMR spectrum in $CDCl_3$ of **16** [(MeCNH-Dipp)(MeCN-Dipp)C(NH-$^{i}$PrCN-$^{i}$Pr)]

## Synthesis of [(MeCN-Dipp)$_2$CH(NH-$^i$PrCN-$^i$Pr)] (**17**)

NacNacH (0.410 g, 1.0 mmol) placed in a Schlenk tube, dissolved in hexane (5 ml) and $^n$BuLi (0.72 ml, 1.2 M, 1.0 mmol) added to give a yellow solution. PMDETA (0.21 ml, 1.0 mmol) and DIC (0.16 ml, 1.0 mmol) added. Colour change from yellow to gold/brown solution observed upon concentration, mixture was placed in -18 °C freezer where it remained throughout the UK Covid-19 pandemic lockdown. After 18 months a small crop of colourless crystals had formed.

**$^1$H NMR (400.1 MHz, $C_6D_6$, 300 K): δ** 7.20-1.18 (m, 3H, CH, $C_6H_3$), 7.16 ($C_6D_6$), 7.09 (m, 2H, CH, $C_6H_3$), 5.02 (s, 1H, CH, γ-CH), 3.41 (p, 4H, CH, $^i$Pr), 2.99 (m, 1H, CH, $^i$Pr), 2.31 (PMDETA), 2.06 (PMDETA), 1.91 (s, 6H, $CH_3$, Me), 1.28 (dd, 24H, $CH_3$, $^i$Pr), 0.91 (dd, 9H, $CH_3$, $^i$Pr) ppm

**$^{13}$C{$^1$H} NMR (100.6 MHz, $CDCl_3$, 300 K): δ** 164.0 ($C_{quaternary}$, C(N$^i$Pr)$_2$), 150.1 ($C_{quaternary}$, CMe), 140.9 ($C_{quaternary}$, $C_6H_3$), 128.0 ($C_6D_6$), 123.4 (CH, $C_6H_3$), 123.1 (CH, $C_6H_3$), 122.7 (CH, $C_6H_3$), 93.1 (CH, γ-CH), 57.8 ($CH_2$, PMDETA), 55.7 ($CH_2$, PMDETA), 49.0 ($CH_3$, PMDETA), 45.5 ($CH_3$, PMDETA), 28.2 (CH, $^i$Pr), 24.3 ($CH_3$, $^i$Pr), 24.1 ($CH_3$, $^i$Pr), 23.6 ($CH_3$, Me) ppm

**IR spectrum:** Insufficient material.

**Melting point analysis**: Insufficient material.

Figure S17: ORTEP diagram of [(MeCN-Dipp)$_2$CH(NH-$^i$PrCN-$^i$Pr)] (**17**)

Molecular structure of **17**, with selected hydrogen atom displayed and organic groups shown as wire frame for clarity. Thermal ellipsoids are displayed at 40 % probability level.

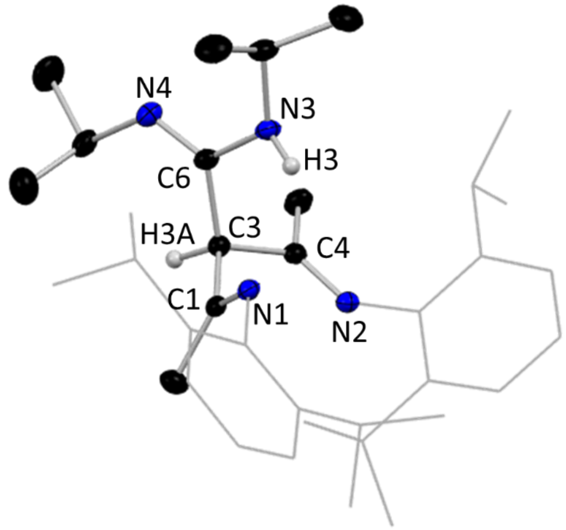


Table S22: Selected bond lengths (Å) and bond angles (°) of **17**

| Atoms | Bond Lengths (Å) |
|---|---|
| N1-C1 | 1.273(19) |
| C1-C3 | 1.524(2) |
| C3-C4 | 1.521(2) |
| C4-N2 | 1.270(19) |
| C3-C6 | 1.534(19) |
| C6-N3 | 1.379(19) |
| C6-N4 | 1.277(19) |
| N3-C7 | 1.465(19) |
| N4-C10 | 1.462(2) |
| **Atoms** | **Bond Angles (°)** |
| C1-C3-C6 | 115.01(12) |
| C4-C3-C6 | 110.29(12) |
| N3-C6-N4 | 120.48(13) |

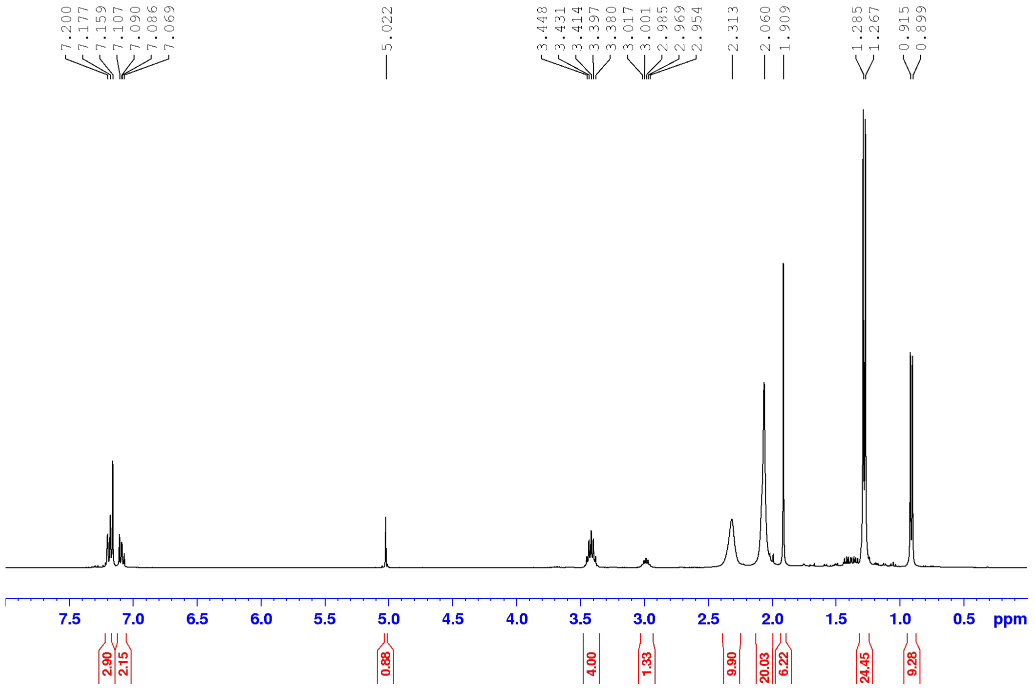


**Figure S17.1**: $^{1}H$ NMR spectrum in $C_6D_6$ of **17** [(MeCN-Dipp)$_2$CH(NH-$^{i}$PrCN-$^{i}$Pr)]

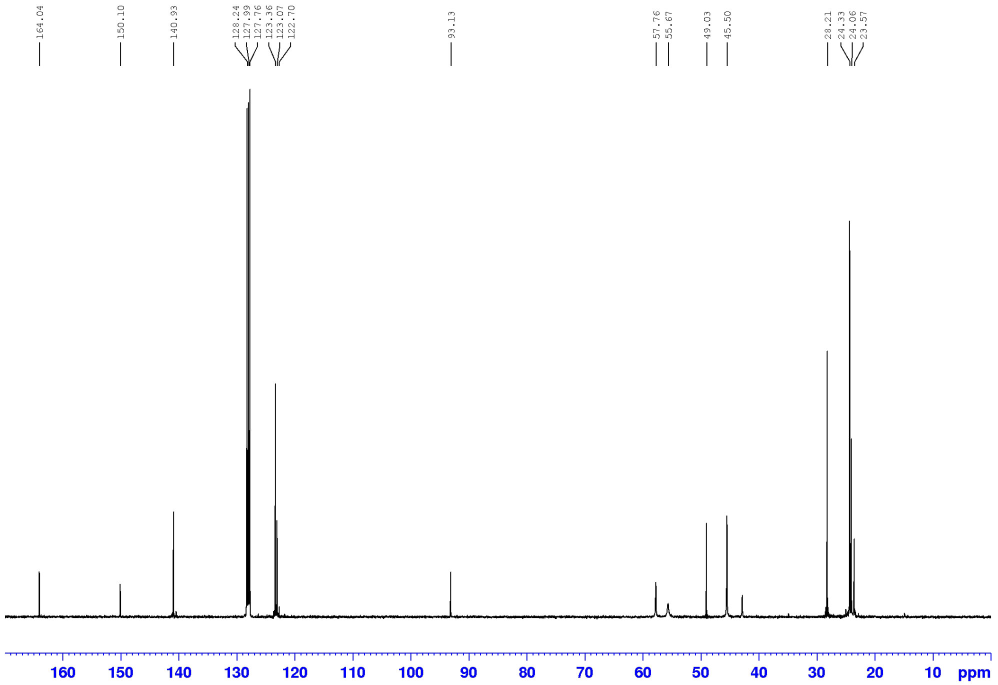


**Figure S17.2**: $^{13}C\{^{1}H\}$ NMR spectrum in $C_6D_6$ of **17** [(MeCN-Dipp)$_2$CH(NH-$^{i}$PrCN-$^{i}$Pr)]

Table S23: Selected X-ray crystal structural data and refinement details for compounds **1-17**

| **Compound** | **1** [(MeCNH-Dipp)(MeCN-Dipp)C($^t$BuNHCO)] | **1a** [{(MeCN-Dipp)$_2$C($^t$BuNHCO)}Li·2THF] | **2a** [{(MeCN-Dipp)$_2$C(4-OMeC$_6$H$_4$NHCO)}Li·2THF] | **3** [(MeCNH-Dipp)(MeCN-Dipp)C(4-MeC$_6$H$_4$NHCO)] | **3a** [{(MeCN-Dipp)$_2$C(4-MeC$_6$H$_4$NHCO)}Li·2THF] | **4** [(MeCNH-Dipp)(MeCN-Dipp)C(2-MeC$_6$H$_4$NHCO)] | **5** [(MeCNH-Dipp)(MeCN-Dipp)C(2,6-Me$_2$C$_6$H$_3$NHCO)].EtOH |
|---|---|---|---|---|---|---|---|
| **CCDC** | 2298370 | 2298371 | 2298372 | 2298373 | 2298374 | 2298375 | 2298376 |
| **Empirical Formula** | $C_{34}H_{51}N_3O$ | $C_{42}H_{66}LiN_3O_3$ | $C_{45}H_{64}LiN_3O_4$ | $C_{37}H_{49}N_3O$ | $C_{45}H_{64}LiN_3O_3$ | $C_{37}H_{49}N_3O$ | $C_{39}H_{55}N_3O_2$ |
| **Molecular Mass** | 517.77 | 667.91 | 717.93 | 551.79 | 701.93 | 551.79 | 597.86 |
| **X-ray Wavelength (Å)** | Mo Kα (λ = 0.71073) | Cu Kα (λ = 1.54184) | Cu Kα (λ = 1.54184) | Cu Kα (λ = 1.54184) | Cu Kα (λ = 1.54184) | Cu Kα (λ = 1.54184) | Cu Kα (λ = 1.54184) |
| **Space Group** | $P2_1/n$ | P-1 | P-1 | P-1 | P-1 | $P2_1/c$ | $Pca2_1$ |
| **Crystal system** | monoclinic | Triclinic | triclinic | triclinic | triclinic | monoclinic | orthorhombic |
| **Temperature (K)** | 123(1) | 100(2) | 100(2) | 100(2) | 100(2) | 100(2) | 100(2) |
| **a/Å** | 10.6772(3) | 9.3798(1) | 8.9764(1) | 10.6807(1) | 8.8114(1) | 13.9795(1) | 23.9798(2) |
| **b/Å** | 23.2998(7) | 12.7676(2) | 11.6785(1) | 18.1547(2) | 11.7153(1) | 8.7269(1) | 12.1099(1) |
| **c/Å** | 12.8824(4) | 17.6482(2) | 20.1645(2) | 19.0465(2) | 20.2669(2) | 27.0841(3) | 24.9659(3) |
| **α/°** | 90 | 90.791(1) | 94.861(1) | 62.273(1) | 94.871(1) | 90 | 90 |
| **β/°** | 96.187(3) | 100.797(1) | 94.332(1) | 85.797(1) | 92.921(1) | 101.962(1) | 90 |
| **γ/°** | 90 | 103.777(1) | 102.153(1) | 84.539(1) | 102.488(1) | 90 | 90 |
| **Volume/Å$^3$** | 3186.17(17) | 2012.58(5) | 2049.57(4) | 3252.53(6) | 2030.07(4) | 3232.45(6) | 7249.92(12) |
| **Z** | 4 | 2 | 2 | 4 | 2 | 4 | 8 |
| **2θmax °** | 56.0 | 146.5 | 145.1 | 145.4 | 145.4 | 146.8 | 145.7 |
| **Measured Reflections** | 81702 | 41662 | 52734 | 52223 | 43985 | 65209 | 62802 |
| **Unique Reflections** | 7616 | 8047 | 8122 | 12909 | 8029 | 65209* | 13878 |
| **$R_{int}$** | 0.0834 | 0.0450 | 0.0213 | 0.0276 | 0.0347 | 0.0872 | 0.0268 |
| **Observed Reflections [I>2σI]** | 5223 | 6927 | 7791 | 11425 | 6977 | 54816 | 13529 |
| **No. Parameters** | 368 | 459 | 506 | 791 | 497 | 390 | 844 |
| **Goodness of Fit** | 1.022 | 1.064 | 1.037 | 1.091 | 1.053 | 1.100 | 1.023 |
| **R [on F, obs refs only]** | 0.0606 | 0.0467 | 0.0370 | 0.0592 | 0.0400 | 0.0860 | 0.0368 |
| **ωR [on F$^2$, all data]** | 0.1493 | 0.1371 | 0.0946 | 0.1617 | 0.1058 | 0.2767 | 0.1027 |
| **Largest diff. peak /hole/Å$^{-3}$** | 0.292/-0.255 | 0.432/-0.259 | 0.223/-0.234 | 0.516/-0.395 | 0.295/-0.297 | 0.347/-0.423 | 0.481/-0.270 |

| Compound | 6<br>[(MeCNH-Dipp)(MeCN-Dipp)C(CyNHCO)].EtOH | 7<br>[(MeCNH-Dipp)(MeCN-Dipp)C(PhNHCO)].2 EtOH | 7.1<br>[(MeCNH-2,6-$^{i}Pr_2C_6H_3)_2$C(PhNHCO)]·THF | 7a<br>[{(MeCN-Dipp)$_2$C(4-$MeC_6H_4$NHCO)}Li·2THF] | 7b<br>[{(MeCN-Dipp)(MeCNH-Dipp)C(PhNCO)}Li]$_4$·2THF.toluene | 8<br>[(MeCNH-Dipp)(MeCN-Dipp)C($^{i}$PrNHCO)] |
|---|---|---|---|---|---|---|
| **CCDC** | 2298377 | 2298378 | 2298379 | 2298380 | 2298381 | 2298382 |
| **Empirical Formula** | $C_{37}H_{57}N_3O_2$ | $C_{38}H_{55}N_3O_3$ | $C_{40}H_{55}N_3O_2$ | $C_{44}H_{62}LiN_3O_3$ | $C_{159}H_{208}Li_4N_{12}O_6$ | $C_{33}H_{49}N_3O$ |
| **Molecular Mass** | 575.85 | 601.85 | 609.87 | 687.90 | 2411.12 | 503.75 |
| **X-ray Wavelength (Å)** | Cu Kα (λ = 1.54184) | Cu Kα (λ = 1.54184) | Cu Kα (λ = 1.54184) | Cu Kα (λ = 1.54184) | Cu Kα (λ = 1.54184) | Cu Kα (λ = 1.54184) |
| **Space Group** | $P2_1$ | Pbca | Pbca | $P2_1/n$ | I2 | Pbca |
| **Crystal system** | monoclinic | orthorhombic | orthorhombic | monoclinic | monoclinic | orthorhombic |
| **Temperature (K)** | 100(2) | 100(2) | 100(2) | 100(2) | 100(2) | 100(2) |
| **a/Å** | 12.1188(3) | 18.1209(1) | 17.7856(3) | 11.9011(2) | 17.4150(2) | 12.7220(2) |
| **b/Å** | 24.4511(4) | 16.4444(1) | 16.5240(2) | 39.1542(5) | 21.3107(3) | 19.5785(3) |
| **c/Å** | 24.1015(4) | 48.5140(3) | 48.7265(6) | 17.6802(2) | 19.8706(2) | 49.9964(7) |
| **α/°** | 90 | 90 | 90 | 90 | 90 | 90 |
| **β/°** | 90.980(2) | 90 | 90 | 103.815(1) | 94.020(1) | 90 |
| **γ/°** | 90 | 90 | 90 | 90 | 90 | 90 |
| **Volume/Å$^3$** | 7140.7(2) | 14456.56(15) | 14320.2(3) | 8000.26(19) | 7356.35(15) | 12453.0(3) |
| **Z** | 8 | 16 | 16 | 8 | 2 | 16 |
| **2θmax °** | 140.0 | 146.4 | 140.0 | 145.3 | 146.5 | 145.4 |
| **Measured Reflections** | 75133 | 88456 | 67305 | 65178 | 26595 | 57165 |
| **Unique Reflections** | 75133* | 14411 | 13539 | 15766 | 12324 | 12294 |
| **$R_{int}$** | 0.0498 | 0.0220 | 0.0852 | 0.0404 | 0.0478 | 0.0382 |
| **Observed Reflections [I>2σI]** | 59408 | 12832 | 11369 | 13342 | 11771 | 10044 |
| **No. Parameters** | 1618 | 861 | 858 | 960 | 886 | 807 |
| **Goodness of Fit** | 1.027 | 1.016 | 1.054 | 1.084 | 1.051 | 1.058 |
| **R [on F, obs refs only]** | 0.0795 | 0.0423 | 0.1061 | 0.0896 | 0.0473 | 0.0591 |
| **ωR [on $F^2$, all data]** | 0.2409 | 0.1091 | 0.2779 | 0.2445 | 0.1304 | 0.1488 |
| **Largest diff. peak /hole/Å$^{-3}$** | 0.572/-0.315 | 0.358/-0.343 | 0.608/-0.423 | 0.417/-0.303 | 0.428/-0.226 | 0.552/-0.278 |

| Compound | 9<br>[(MeCNH-Dipp)(MeCN-Dipp)C(EtNHCO)] | 9a<br>[{(MeCN-Dipp)$_2$C(EtNHCO)}Li·THF]$_2$ | 10<br>[(MeCNH-Dipp)(MeCN-Dipp)C{C(O)N(C$_6$H$_4$OMe)C(O)N(H)C$_6$H$_4$OMe}] | 11<br>[(MeCNH-Dipp)(MeCN-Dipp)C(4-MeC$_6$H$_4$NHCS)] | 11a<br>[{(MeCN-Dipp)$_2$CH(4-MeC$_6$H$_4$NHCS)}Li·THF] | 12<br>[(MeCNH-Dipp)(MeCN-Dipp)C($^t$BuNHCS)] |
|---|---|---|---|---|---|---|
| CCDC | 2298383 | 2298384 | 2298385 | 2298386 | 2298387 | 2298388 |
| Empirical Formula | $C_{32}H_{47}N_3O$ | $C_{80}H_{124}Li_2N_6O_6$ | $C_{45}H_{56}N_4O_4$ | $C_{37}H_{49}N_3S$ | $C_{45}H_{64}LiN_3O_2S$ | $C_{34}H_{51}N_3S$ |
| Molecular Mass | 489.72 | 1279.72 | 716.93 | 567.85 | 717.99 | 533.83 |
| X-ray Wavelength (Å) | Cu Kα (λ = 1.54184) | Cu Kα (λ = 1.54184) | Cu Kα (λ = 1.54184) | Cu Kα (λ = 1.54184) | Cu Kα (λ = 1.54184) | Cu Kα (λ = 1.54184) |
| Space Group | P-1 | P-1 | C2/c | P-1 | C2/c | $P2_1/n$ |
| Crystal system | triclinic | triclinic | monoclinic | triclinic | monoclinic | monoclinic |
| Temperature (K) | 100(2) | 100(2) | 100(2) | 100(2) | 100(2) | 100(2) |
| a/Å | 10.3763(1) | 12.5415(4) | 36.1680(10) | 10.7157(3) | 17.3623(1) | 10.5704(1) |
| b/Å | 15.5437(2) | 12.5602(4) | 9.3438(1) | 11.1222(3) | 16.3139(1) | 24.5114(4) |
| c/Å | 20.5455(2) | 14.4976(4) | 29.3254(8) | 16.6424(6) | 29.6995(2) | 12.5262(2) |
| α/° | 106.822(1) | 106.503(2) | 90 | 83.814(3) | 90 | 90 |
| β/° | 102.634(1) | 101.638(3) | 126.404(4) | 73.808(3) | 96.708(1) | 94.375(1) |
| γ/° | 92.543(1) | 112.001(3) | 90 | 61.632(3) | 90 | 90 |
| Volume/Å$^3$ | 3074.47(6) | 1904.32(11) | 7976.4(5) | 1675.34(10) | 8354.70(9) | 3236.02(8) |
| Z | 4 | 1 | 8 | 2 | 8 | 4 |
| 2θmax ° | 145.7 | 146.4 | 146.5 | 140.0 | 145.4 | 146.8 |
| Measured Reflections | 49541 | 25860 | 68859 | 25555 | 103068 | 64783 |
| Unique Reflections | 12186 | 7605 | 7981 | 6343 | 8300 | 6473 |
| $R_{int}$ | 0.0232 | 0.0888 | 0.0322 | 0.0408 | 0.1110 | 0.0476 |
| Observed Reflections [I>2σI] | 10779 | 6401 | 7221 | 6054 | 7998 | 5861 |
| No. Parameters | 735 | 450 | 544 | 411 | 484 | 501 |
| Goodness of Fit | 1.042 | 1.040 | 1.063 | 1.133 | 1.071 | 1.079 |
| R [on F, obs refs only] | 0.0407 | 0.0621 | 0.0686 | 0.0994 | 0.0677 | 0.0719 |
| ωR [on F$^2$, all data] | 0.1102 | 0.1908 | 0.2000 | 0.2723 | 0.1915 | 0.2064 |
| Largest diff. peak /hole/Å$^{-3}$ | 0.416/-0.319 | 0.631/-0.546 | 0.382/-0.291 | 1.074 /0.434 | 0.813/-0.451 | 0.572/-0.293 |

| Compound | 13a [(MeCNH-Dipp)(MeCN-Dipp)C(PhNHCS)] | 14 [(MeCNH-Dipp)(MeCN-Dipp)C(EtNHCS)] | 14a [{(MeCN-Dipp)$_2$CH(EtNCS)}Li] | 15 [(N-Dipp)C(CH$_3$)C(C(O)CH$_3$)C(NPh)S] | 16 [(MeCNH-Dipp)(MeCN-Dipp)C(NH-$^i$PrCN-$^i$Pr)] | 17 [(MeCN-Dipp)$_2$CH(NH-$^i$PrCN-$^i$Pr)] |
|---|---|---|---|---|---|---|
| CCDC | 2298389 | 2298390 | 2298391 | 2298392 | 2298393 | 2298394 |
| Empirical Formula | $C_{44}H_{62}LiN_3O_2S$ | $C_{32}H_{47}N_3S$ | $C_{32}H_{46}LiN_3S$ | $C_{24}H_{28}N_2OS$ | $C_{36}H_{56}N_4$ | $C_{36}H_{56}N_4$ |
| Molecular Mass | 703.96 | 505.78 | 511.72 | 392.54 | 544.84 | 544.84 |
| X-ray Wavelength (Å) | Cu Kα (λ = 1.54184) | Cu Kα (λ = 1.54184) | Cu Kα (λ = 1.54184) | Cu Kα (λ = 1.54184) | Cu Kα (λ = 1.54184) | Mo Kα (λ = 0.71073) |
| Space Group | C2/c | P-1 | Pnma | C2/c | $P2_1/n$ | $P2_1/n$ |
| Crystal system | monoclinic | triclinic | orthorhombic | monoclinic | monoclinic | monoclinic |
| Temperature (K) | 100(2) | 100(2) | 100(2) | 100(2) | 100(2) | 123(2) |
| a/Å | 17.4378(1) | 10.7566(1) | 20.3250(4) | 19.1031(3) | 10.3476(1) | 8.9017(4) |
| b/Å | 16.4630(1) | 14.9826(2) | 22.5724(4) | 13.1154(2) | 26.5676(3) | 22.6000(10) |
| c/Å | 29.2206(2) | 20.4206(2) | 6.5250(1) | 17.1154(2) | 12.3488(1) | 17.5432(7) |
| α/° | 90 | 104.718(1) | 90 | 90 | 90 | 90 |
| β/° | 99.198(1) | 102.225(1) | 90 | 95.202(1) | 94.130(1) | 98.902(4) |
| γ/° | 90 | 92.800(1) | 90 | 90 | 90 | 90 |
| Volume/Å$^3$ | 8280.74(9) | 3092.46(6) | 2993.57(9) | 4270.51(11) | 3386.00(6) | 3486.8(3) |
| Z | 8 | 4 | 4 | 8 | 4 | 4 |
| 2θmax ° | 146.2 | 145.3 | 146.6 | 146.3 | 145.7 | 58.0 |
| Measured Reflections | 45137 | 51959 | 29115 | 15300 | 70473 | 74216 |
| Unique Reflections | 8262 | 12239 | 3068 | 4230 | 6726 | 8838 |
| $R_{int}$ | 0.0266 | 0.0394 | 0.0686 | 0.0537 | 0.0271 | 0.0939 |
| Observed Reflections [I>2σI] | 7715 | 11023 | 2801 | 3673 | 6320 | 6077 |
| No. Parameters | 483 | 735 | 188 | 259 | 383 | 379 |
| Goodness of Fit | 1.031 | 1.048 | 1.139 | 1.061 | 1.037 | 1.018 |
| R [on F, obs refs only] | 0.0413 | 0.0494 | 0.0770 | 0.0479 | 0.0455 | 0.0584 |
| ωR [on F$^2$, all data] | 0.1108 | 0.1469 | 0.2153 | 0.1398 | 0.1188 | 0.1337 |
| Largest diff. peak /hole/Å$^{-3}$ | 0.342/-0.239 | 0.556/-0.440 | 0.793/-0.415 | 0.398/-0.249 | 0.517/-0.224 | 0.260/-0.199 |

*Refined as a twin using a hklf 5 formatted reflection file.

# References


[1] M. Stender, R. J. Wright, B. E. Eichler, J. Prust, M. M. Olmstead, H. W. Roesky, P. P. Power, *J. Chem. Soc. Dalton Trans.* **2001**, 3465–3469.

[2] Crysalis Pro, Agilent Technologies Ltd., Yarnton, Oxfordshire, UK, **2014**.

[3] G. M. Sheldrick, *Acta Crystallogr. Sect. A Found. Crystallogr.* **2015**, *71*, 3–8.

[4] O. V. Dolomanov, L. J. Bourhis, R. J. Gildea, J. A. K. Howard, H. Puschmann, *J. Appl. Crystallogr.* **2009**, *42*, 339–341.

[5] L. J. Farrougia, *J. Appl.Crystallogr.* **2012**, *45*, 849-854.

[6] R. M. Gauld, R. McLellan, A. R. Kennedy, F. J. Carson, J. Barker, J. Reid, C. T. O'Hara, R. E. Mulvey, *Eur. J. Inorg. Chem.* **2021**, 1615–1622.
